# Korea hypertension fact sheet 2018

**DOI:** 10.1186/s40885-018-0098-0

**Published:** 2018-10-01

**Authors:** The Korean Society Hypertension (KSH), Jin-Won Jeong, Gilja Shin, Dong Soo Kim, Gheun-Ho Kim, Myeong-Chan Cho, Seok-Min Kang, Wook Bum Pyun, Hae-Young Lee, Wook-Jin Chung, Sang-Hyun Ihm, Kwang Il Kim, Eun Joo Cho, Il-Suk Sohn, Sungha Park, Jinho Shin, Ki Chul Sung, Sung Kee Ryu, Jidong Sung, Dae Jung Kim, Hyeon Chang Kim, Dong-Ryeol Ryu, Dong Heon Yang, Byung Su Yoo, Moo-Yong Rhee, Seung Hoon Lee, Eun Mi Lee, Joong Hwa Chung, Jin Ok Jeong, Jin Han, Young Mi Hong, Jin Yong Hwang, Chang Gyu Park, Se-Joong Rim, Hypertension Epidemiology Research Working Group, Hyeon Chang Kim, Song Vogue Ahn, Sun Ha Jee, Sungha Park, Hae-Young Lee, Min Ho Shin, Sang-Hyun Ihm, Jong Ku Park, Il Suh, Tae-Yong Lee, Seung Won Lee, Hyeon Chang Kim, Myeong-Chan Cho

**Affiliations:** 1 Department of Preventive Medicine, Yonsei University College of Medicine, Seoul, 03722 South Korea; 2 Department of Internal Medicine, Chungbuk National University College of Medicine, Cheongju, 28644 South Korea

**Keywords:** Hypertension, Prevalence, Awareness, Treatment, Control, Anti-hypertensive medication, Korea

## Abstract

**Background:**

The Korea Hypertension Fact Sheet 2018 aims to overview the magnitude and management status of hypertension, and their trends in Korea.

**Methods:**

The Hypertension Epidemiology Research Group analyzed the 1998–2016 Korea National Health and Nutrition Examination Survey data and the 2002–2016 Korea National Health Insurance Big Data.

**Results:**

The population average of systolic/diastolic blood pressure was 118/77 mmHg among Korean adults (age 30+) in 2016, showing little change in recent 10 years. However, the number of people with hypertension increased steadily, exceeding 11 million. The number of people diagnosed with hypertension increased from 3 million in 2002 to 8.9 million in 2016. The number of people using antihypertensive medication increased from 2.5 million in 2002 to 8.2 million in 2016. However, only 5.7 million people are being treated constantly. Hypertension awareness, treatment, and control rates increased fast until 2007, but showed a plateau thereafter. More than half of the young hypertensive patients (30–49 years) did not know about and treat for their hypertension. Among patients prescribed antihypertensive medications, 45% was elderly people over the age of 65 years, 57% used anti-diabetic or cholesterol-lowering medications, and 60% were prescribed two or more class of antihypertensive medications simultaneously.

**Conclusions:**

In Korea, the level of hypertension management has considerably improved over the last 20 years. In order to achieve further improvement in hypertension management status, we need to find the vulnerable subgroups and develop subgroup-specific intervention strategies. It is also becoming more important to manage hypertensive patients at older age and those with concurrent chronic diseases.

**Electronic supplementary material:**

The online version of this article (10.1186/s40885-018-0098-0) contains supplementary material, which is available to authorized users.

## Background

Hypertension is not only the most important modifiable risk factor for cardiovascular and cerebrovascular diseases, but also the most contributable factor of the disease burden in the world [1, 2]. In the twenty-first century, mortality from cardiovascular and cerebrovascular diseases account for nearly one half of all deaths in the developed region, and for one quarter in the developing region [3]. In Korea, cerebrovascular and cardiovascular diseases rank first and second for mortality as single-organ diseases [4]. Total medical cost for hypertension was estimated to 2850 billion Korean won which accounts for 13.4% of medical cost due to all chronic disorders [5, 6]. This means that controlling the hypertension is crucial to reducing the overall burden of disease in society and improving quality of life. To reduce and control hypertension, continuous monitoring of the magnitude and management status of hypertension should be the first step. Thus the Korean Society Hypertension (KSH) – Hypertension Epidemiology Research Working Group analyzed national representative datasets to overview of the magnitude and management status of hypertension and their trends between 1998 and 2016 in Korea.

## Methods

The Korea Hypertension Fact Sheet 2018 analyzed two national representative datasets. The first one is the Korea National Health and Nutrition Examination Survey (KNHANES) from 1998 to 2016. The KNHANES is a national surveillance system in Korea that assesses the health and nutritional status of Koreans since 1998 [7]. In the KNHANES from 1998 to 2016, adults over 30 years of age were included in the current analysis. The second one is the National Health Insurance (NHI) Big Data from 2002 to 2016. The NHI Big Data is a public database on health care utilization, health screening, socio-demographic variables, and mortality for the whole population of South Korea, formed by the National Health Insurance Service [8]. In the NHI Big Data from 2002 to 2016, people of all ages were included in the current analysis.

### Analysis of the KNHANES from 1998 to 2016

Hypertension is defined as systolic blood pressure (SBP) ≥ 140 mmHg, diastolic blood pressure (DBP) ≥ 90 mmHg or self-reported use of antihypertensive medication. Awareness rate was defined as the proportion of people who reported to be diagnosed with hypertension from physicians among all people with hypertension. The treatment rate was defined as the proportion of people using antihypertensive drugs for 20 days or more per month among all people with hypertension. The control rate was defined as the proportion of people who having SBP < 140 mmHg and DBP < 90 mmHg with antihypertensive medication, among the all people with hypertension as well as among the treated people with hypertension. To evaluate the magnitude and management status of hypertension excluding the effects of population aging, an indirect standardization method was used. The indirect age standardization is based on the demographics of the Korean population in 2005 from the Population and Housing Census performed by Statistics Korea.

### Analysis of the NHI big data from 2002 to 2016

The medical service use was defined as any case of person seeking medical service with diagnosis of essential hypertension at least once a year, and the cases were identified from the NHI claim database. Treatment of hypertension was defined as the case of person who receives prescription of any antihypertensive medications at least once per every year. The sustained treatment that reflects persistence was defined as the case of person who receives antihypertensive prescriptions for at least 290 days (80%) per year. If the prescription of hypertension treatment had changed several times, the prescription for the longest duration every year was used as the representative prescription of the patient for the year, and the use of the medication was classified accordingly. Antihypertensive medications were classified into diuretics, beta-blockers, calcium channel blockers, angiotensin receptor blockers, angiotensin-converting enzyme inhibitors, aldosterone antagonists, alpha-blockers, vasodilators, and others.

## Results

### Magnitude of hypertension

Among the adult Korean population aged 30 years or older, age-standardized mean SBP/DBP was 129/82 mmHg for men and 126/78 mmHg for women in 1998, and 121/79 mmHg for men and 114.8 for men and 115/74 mmHg for women in 2016 (Additional files 1 and 2 Page 11). Over the last 10 years, the age-standardized mean blood pressure levels remained almost unchanged. The age-standardized prevalence of hypertension also show little change from 29.8% (men 32.4%, women 26.8%) in 1998 to 29.1% (men 35.0%, women 22.9%) in 2016 (Additional files 1 and 2, page 12). However, due to the rapid population aging, the number of people with hypertension has steadily increased from 7.6 million in 1998 to over 11 million recently (Additional files 1 and 2, page 14). Hypertension prevalence increased with age in both sexes (Additional files 1 and 2, page 13). But the sex-specific prevalence was higher in men until the age of 60s, but higher in women from the age of 70s.

### Hypertension management status

Awareness, treatment, and control rates of hypertension, which are frequently used indicators of hypertension management, increased all rapidly from 1998 to 2007, but thereafter remained almost unchanged (Additional files 1 and 2, page 17). The awareness rate increased from 25% in 1998 to 65% in 2007, and still remained at 65% in 2016. The treatment rate increased from 22% in 1998 to 59% in 2007 and 61% in 2016. The control rate increased from 5% in 1998 to 41% in 2007 and to 44% in 2016.

In stratifying by sex, hypertension awareness rate in 2016 was 64.9% for men and 66.8% for women (Additional files 1 and 2, page 18). The treatment rate of hypertension in 2016 was 60.1% for men and 64.3% for women (Additional files 1 and 2, page 19). Control rate among all people with hypertension was 43.4% for men and 47.4% in women, suggesting that 56% of hypertensive patients are still unable to control their blood pressure (Additional files 1 and 2, page 20). Control rate among treated people with hypertension was 71.0% for men and 70.6% in women (Additional files 1 and 2, page 21). Overall, women showed better management status of hypertension compared to men. In age-sex specific analysis, the younger people with hypertension (30–49 years) in both sexes still have awareness, treatment, and control rates below 50% (Additional files 1 and 2, pages 22–24).

### Medical service uses for hypertension

The number of people diagnosed with hypertension in medical institution has increased by about three times from 3 million in 2002 to 8.9 million in 2016 (Additional files 1 and 2, page 27). People who receiving antihypertensive prescription also increased more than three times from 2.5 million in 2002 to 8.2 million in 2016. However, only 5.7 million people are constantly being treated in 2016, which is approximately 64% of all hypertensive patients.

Among people receiving antihypertensive prescription, the proportion of people aged 65 or older increased from 34% in 2002 to 46% in 2016 (Additional files 1 and 2, pages 28, 29). The proportion of hypertensive people who are receiving diabetes or dyslipidemia treatment as well as people receiving antihypertensive prescription also rapidly increased from 25% in 2002 to 57% in 2016 (Additional files 1 and 2, pages 30, 31).

In 2002, 57% of the treated patients were prescribed single class of antihypertensive medications (Additional files 1 and 2, pages 32, 33). However, in 2016, only 40% of the treated patients were prescribed one class of antihypertensive medication, 42% were prescribed two classes, and 18% were prescribed three or more classes. Calcium channel blockers have been the most widely used as single class of antihypertensive medications for a long time, but the use of angiotensin receptor blockers has increased rapidly and has been prescribed more than calcium channel blockers for the first time in 2016 (Additional files 1 and 2, pages 36, 37). The most common antihypertensive regimen is a combination of calcium channel blockers and angiotensin blockers (Additional files 1 and 2, pages 40–43).

## Discussion

The Korea Hypertension Fact Sheet 2018 provides an overview on the magnitude and management status of hypertension in Korea. Although the population average of blood pressure has been showing little change in recent 10 years, the absolute number of people with hypertension increased steadily, exceeding 11 million due to the population aging. People who receiving antihypertensive prescription also increased, but people are constantly being treated is approximately 64% of all hypertensive patients in 2016. Hypertension management status in Korea had been greatly improved until 2007, but thereafter there has been little further improvement. Overall, women had a better management status of hypertension than men. However, in younger age groups, both men and women showed lower levels of hypertension management than other age groups. To achieve further improvement in hypertension management, it is important to increase awareness and treatment rates for younger people with hypertension. It is another challenge to manage hypertensive people with old age and those with concurrent chronic diseases such as diabetes and dyslipidemia. We also need to develop customized prevention and management strategies which fit various subgroup characteristics. Emphasizing the importance of persistent treatment and blood pressure control to prevent complications and death from hypertension is still a top priority.

## Conclusions

In Korea, the level of hypertension management has substantially improved over the last 20 years. In order to achieve further improvement in hypertension management status and quality of life, we need to find the vulnerable subgroups and develop subgroup-specific intervention strategies, suggesting that more carefully managing hypertensive patients at older age and those with concurrent chronic diseases.

## Additional files

Additional file 1: Korea hypertension fact sheet 2018 - Extended English version. (PDF 1648 kb)

Additional file 2: Korea hypertension fact sheet 2018 - Extended Korean version. (PDF 1208 kb)
