# Supplementary material for: Korea hypertension fact sheet 2018
Source: Clin Hypertens. 2018 Oct 1;24:13. doi: 10.1186/s40885-018-0098-0 (PMC6166277; doi:10.1186/s40885-018-0098-0)
Supplement: Supplementary file 1 — Korea hypertension fact sheet 2018 - Extended English version. (PDF 1648 kb) [file 40885_2018_98_MOESM1_ESM.pdf]

KOREA HYPERTENSION FACT SHEET 2018

The Korean Society of Hypertension

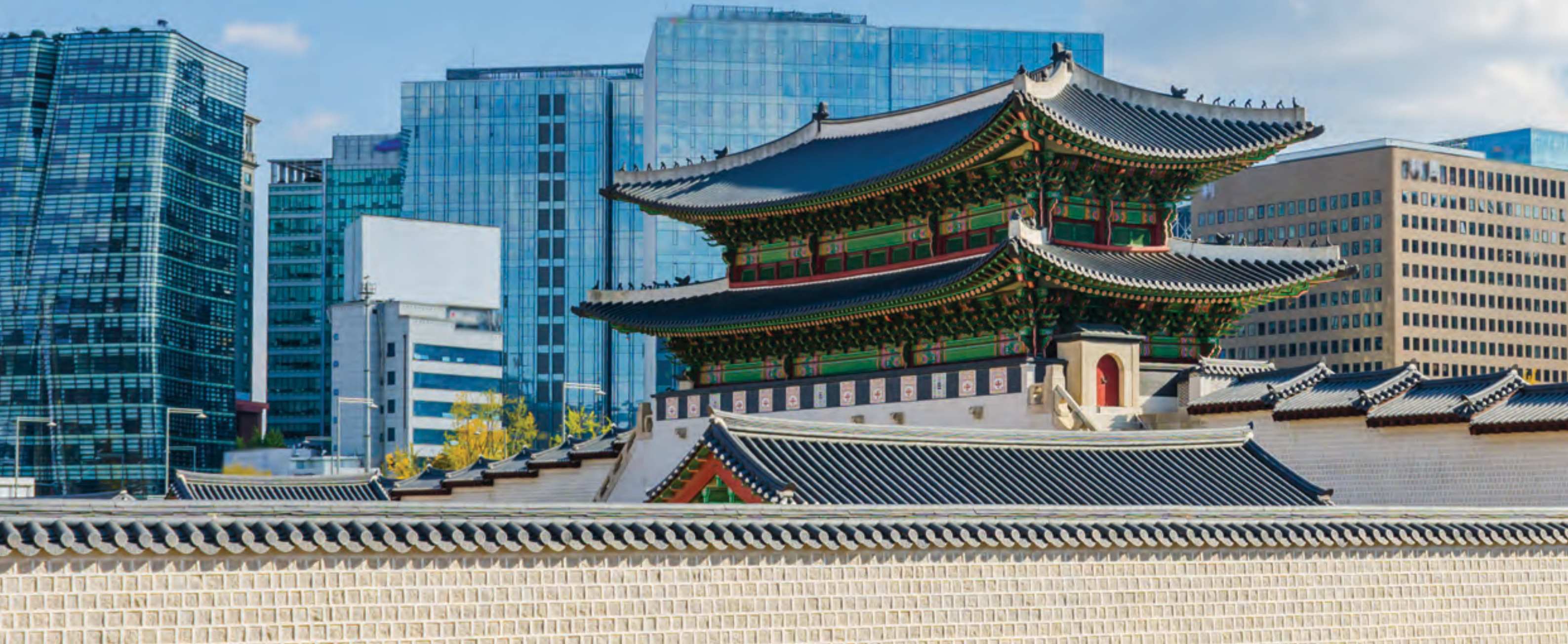

KOREA HYPERTENSION FACT SHEET 2018

Publisher Myeong-Chan Cho
Editor-in-chief Hyeon Chang Kim
Editor The Korean Society of Hypertension - Hypertension Epidemiology Research Working Group

Executive Members of The Korean Society of Hypertension

Chairman	Jin-Won Jeong	Wonkwang University
Chiarman-Elect	Gilja Shin	Ewha Womans University
Vice-Chairman	Dong Soo Kim	Inje University
	Gheun-Ho Kim	Hanyang University
President	Myeong-Chan Cho	Chungbuk National University
Secretary General	Seok-Min Kang	Yonsei University
Director of Planning	Wook Bum Pyun	Ewha Womans University
Director of Scientific Affairs	Hae-Young Lee	Seoul National University
Director of Publication	Wook-Jin Chung	Gachon University
Director of Treasurer	Sang-Hyun Ihm	The Catholic University of Korea
Director of Research	Kwang Il Kim	Seoul National University
Director of Education	Eun Joo Cho	The Catholic University of Korea
Director of Public Relation	Il-Suk Sohn	The Kyunghee University
Director of International Affairs	Sungha Park	Yonsei University
Director of Health Insurance	Jinho Shin	Hanyang University
Director of Public Policy	Ki Chul Sung	Sungkyunkwan University
Director of Informatics	Sung Kee Ryu	Eulji University
Director of Ethics	Jidong Sung	Sungkyunkwan University

Directors	Dae Jung Kim	Ajou University
	Hyeon Chang Kim	Yonsei University
	Dong-Ryeol Ryu	Ewha Womans University
	Dong Heon Yang	Kyungpook National University
	Byung Su Yoo	Yonsei University Wonju
	Moo-Yong Rhee	Dongguk University
	Seung Hoon Lee	Seoul National University
	Eun Mi Lee	Wonkwang University
	Joong Hwa Chung	Chosun University
	Jin Ok Jeong	Chungnam National University
	Jin Han	Inje University
	Young Mi Hong	Ewha Womans University
	Jin Yong Hwang	Gyeong-sang National University
Auditors	Chang Gyu Park	Korea University
	Se-Joong Rim	Yonsei University

Executive Members of The Hypertension Epidemiology Research Working Group

Chairman	Hyeon Chang Kim	Yonsei University
Vice-Chairman	Song Vogue Ahn	Ewha Womans University
Executive Committee Members	Sun Ha Jee	Yonsei University
	Sungha Park	Yonsei University
	Hae-Young Lee	Seoul National University
	Min Ho Shin	Seoul National University
	Sang-Hyun Ihm	The Catholic University of Korea

Advisors	Jong Ku Park	Yonsei University
	Il Suh	Yonsei University
	Tae-Yong Lee	Chungnam National University
Coordinator	Seung Won Lee	Yonsei University

Greetings

Hypertension is the most common chronic disorder in Korea. Globally, it stands as the top risk factor for cardiovascular diseases and the number one cause of death. However, we can effectively decrease the burden of hypertension through prevention and management of the condition. Since its establishment in 1994, the Korean Society of Hypertension is confident in that it has made significant attributions in raising our country's hypertension awareness, treatment, and control rates through consistent academic achievements and successful healthcare programs. We have envisioned to improve people's health by better blood pressure control and to become the field's global leader by providing scientific evidence. With emphasis on responsibility, participation, leadership, and professionalism, we collaborate with governmental and non-governmental organizations to establish practical hypertension management goals and strive to achieve them. We have concluded that it is crucial to provide a well-rounded and nationally-representative data on hypertension. This hypertension fact sheet will provide an objective insight into the magnitude and management status of hypertension in Korea, and will be the basis for academic research and policy making on hypertension. We deeply acknowledge the "Korea Centers for Disease Control & Prevention (KCDC)" and the "National Health Insurance Service (NHIS)" for providing valuable dataset, and the "Hypertension Epidemiology Research Working Group" for analyzing and interpreting such vast dataset.

The Korean Society of Hypertension

Chairman
Jin-Won Jeong

President
Myeong-Chan Cho

대한고혈압학회
THE KOREAN SOCIETY OF HYPERTENSION

Data source and analysis methods - Korea National Health and Nutrition Examination Survey (KNHANES)

**Subjects**		30 years of age and older / From 1998 to 2016
**Classification of blood pressure**	Hypertension	① Systolic blood pressure (SBP) greater than 140mmHg, or<br>② Diastolic blood pressure (DBP) greater than 90mmHg, or<br>③ Currently taking antihypertensive medication
	Prehypertension	① Not qualified for hypertension and<br>② SBP between 120-139mmHg, or<br>③ DBP between 80-89mmHg
	Normal blood pressure	None of the above
**Definition of indicators**	Awareness rate	Proportion of people clinically diagnosed with hypertension over people with hypertension
	Treatment rate	Proportion of people taking antihypertensive medications for 20 days or more per month over people with hypertension
	Control rate (among people with hypertension)	Proportion of people with SBP <140mmHg and DBP <90mmHg over people with hypertension
	Control rate (among people treated for hypertension)	Proportion of people with SBP <140mmHg and DBP <90mmHg over people treated for hypertension

Data source and analysis methods - National Health Insurance Big Data (NHI-BD)

**Subjects**		All ages / From 2002 to 2016
**Definition of indicators**	Healthcare services utilization	Clinical visit with the diagnosis of hypertension at least once per year
	Treatment	Prescribed antihypertensive medication at least once per year
	Persistent treatment	Prescribed antihypertensive medication 290 days or more per year (80% of one calendar year)
**Hypertension treatment**	Classification of antihypertensive drugs	Diuretics (DU), beta-blocker (BB), calcium channel blocker (CCB), angiotensin receptor blocker (ARB), ACE inhibitor (ACE), others (aldosterone antagonists, alpha blockers, vasodilators, etc)
	Primary prescription	If alterations are made to prescription, primary prescription is defined as and classified by antihypertensive medication that has been used for the longest time in that particular year.

Contents

06 Summary
07 Summary of population statistics
08 Summary of treatment statistics

1 Changes in population mean blood pressure and magnitude of hypertension

11 Changes in population mean blood pressure
12 Changes in prevalence
13 Changes in prevalence by sex and age
14 Changes in the estimated number of people with hypertension

2 Changes in indicators of hypertension management

16 Prevalence and management indicators by sex
17 Changes in management indicators
18 Changes in awareness rate by sex
19 Changes in treatment rate by sex
20 Changes in control rate by sex
22 Changes in awareness rate by sex and age
23 Changes in treatment rate by sex and age
24 Changes in control rate by sex and age

3 Healthcare services utilization for hypertension

27 Changes in healthcare services utilization
28 Age distribution of people treated for hypertension
30 Co-treatment for hypertension, dyslipidemia, or diabetes
32 Changes in antihypertensive drug prescription
36 Changes in monotherapy composition
40 Changes in dual therapy composition

Summary

- Approximately 11 million Korean adults have hypertension.
- In adults aged 30 years or older, the prevalence of hypertension is 29%. Of those, 65% are aware of their diagnosis, 61% are receiving treatment, and 44% have their blood pressures under control.
- Awareness, treatment and control rates of hypertension have rapidly improved until 2007; but since then, these rates have been stagnant.
- People with hypertension aged 30-49 years have awareness, treatment, and control rates below 50%.
- Annually, 8.9 million people make clinical visit at least once to receive treatment for hypertension. Of those, 5.7 million receive persistent treatment.
- Among those treated for hypertension, the proportion of individuals aged 65 years or older has increased rapidly from 34% in 2002 to 46% in 2016.
- Among those treated for hypertension, 57% are also treated for diabetes or dyslipidemia.
- Among those treated for hypertension, 60% use a combination of two or more antihypertensive drugs.
- The most frequently used monotherapy drugs are angiotensin receptor blockers (ARBs, 43%) and calcium channel blockers (CCBs, 43%). The most frequently used dual therapy is a combination of CCBs and ARBs (54%).

Summary of population statistics

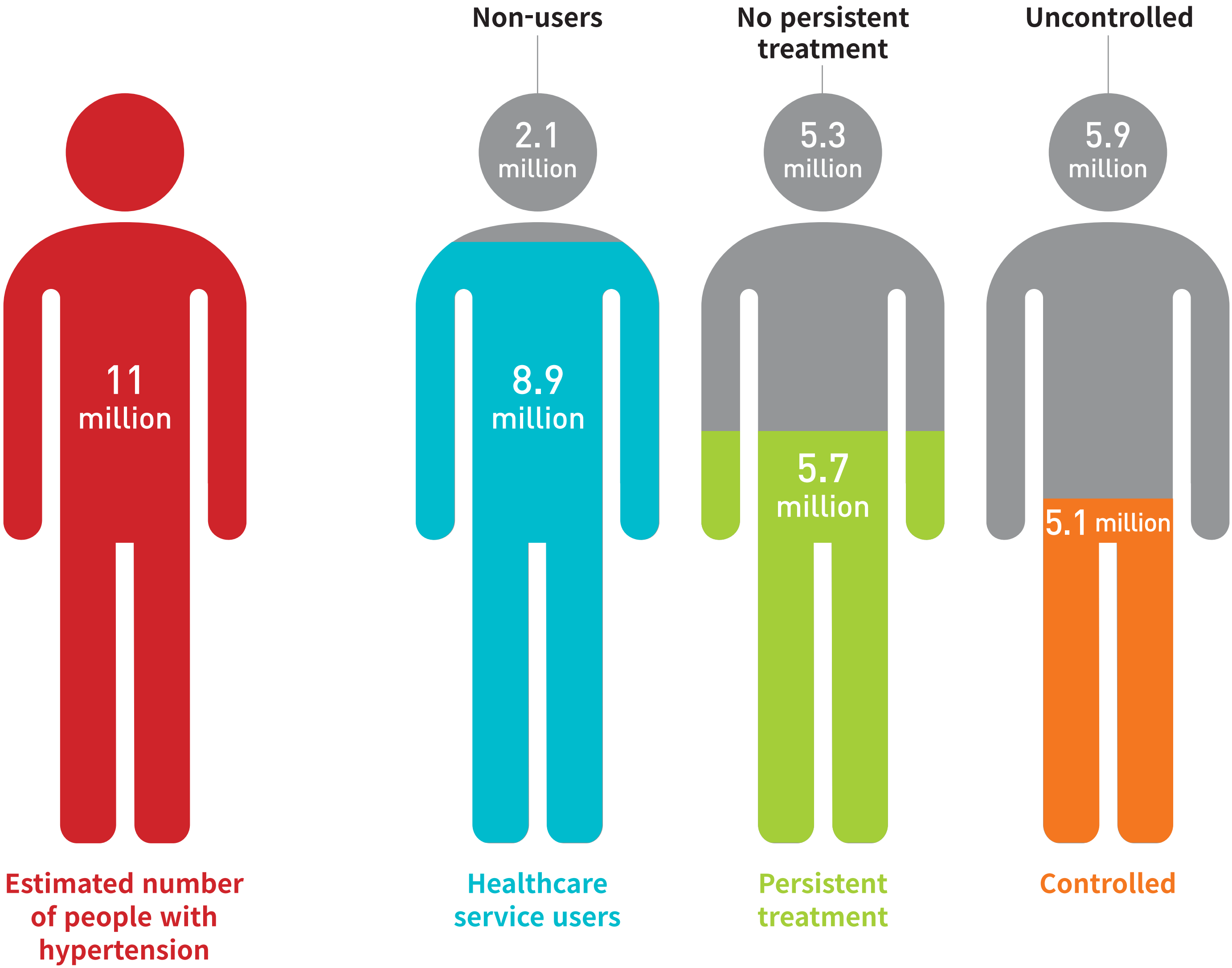

Data source: KNHANES (2016), NHIBD (2016)

Summary of treatment statistics

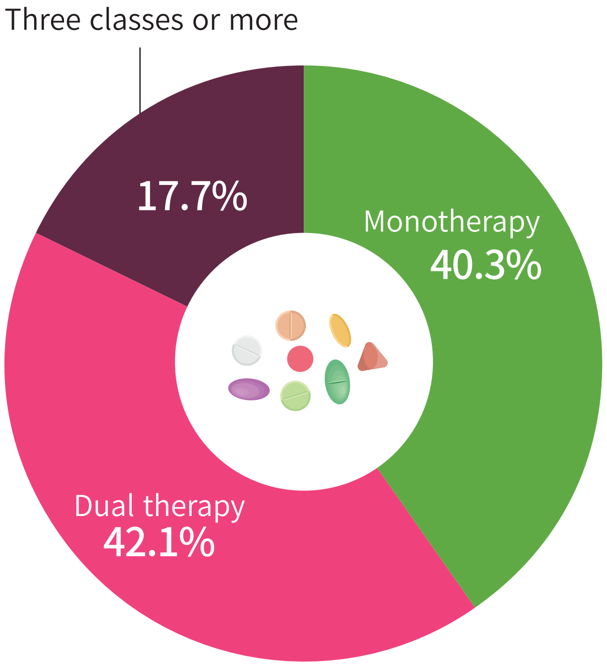

Medication prescription
(total 8,219,104)

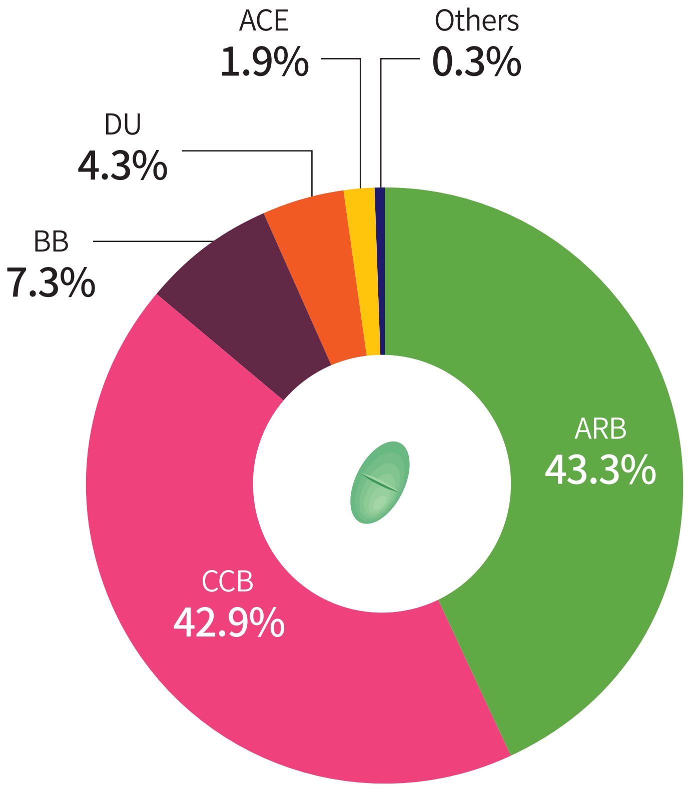

Monotherapy composition
(total 3,311,114)

Data source: NHIBD (2016)

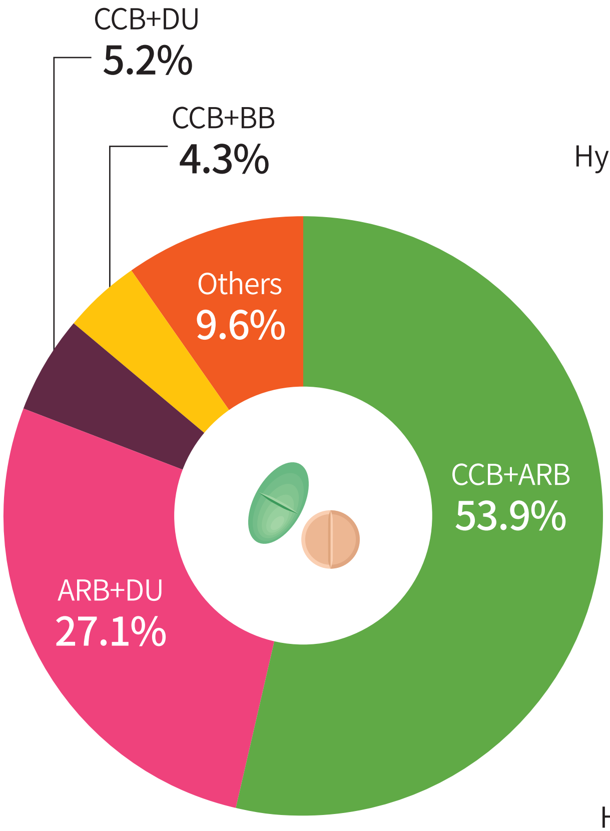

Dual therapy composition
(total 3,456,866)

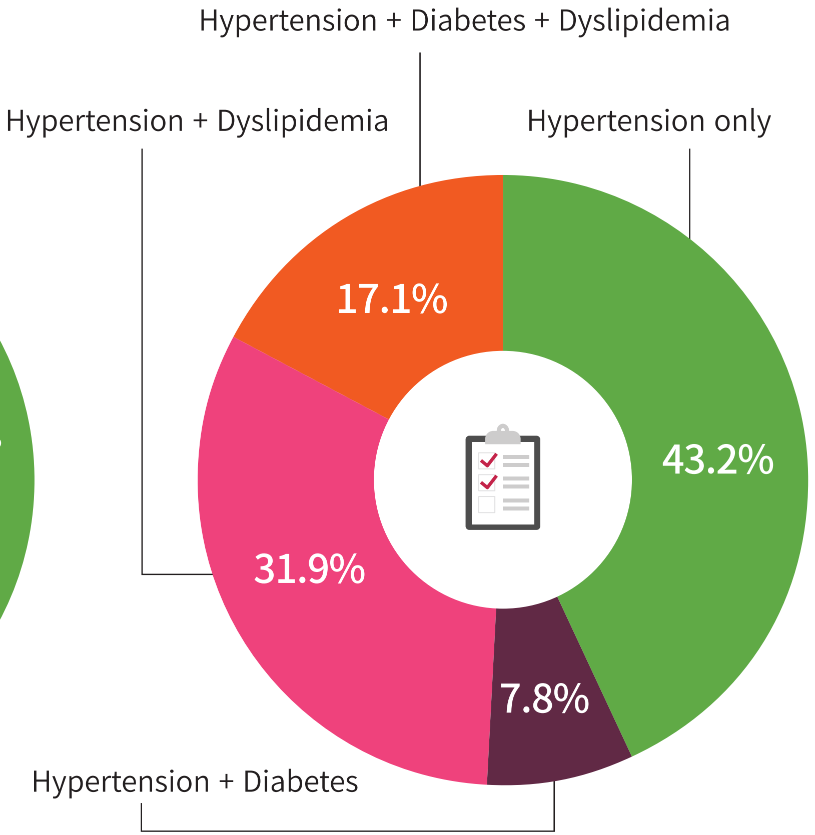

Co-treatment for hypertension, diabetes, or dyslipidemia
(total 8,219,104)

 Data source: NHIBD (2016)

1 Changes in population mean blood pressure and hypertension magnitude

Data source: KNHANES

Changes in population mean blood pressure

(Age 30+, age-standardized)

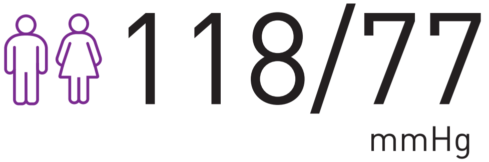

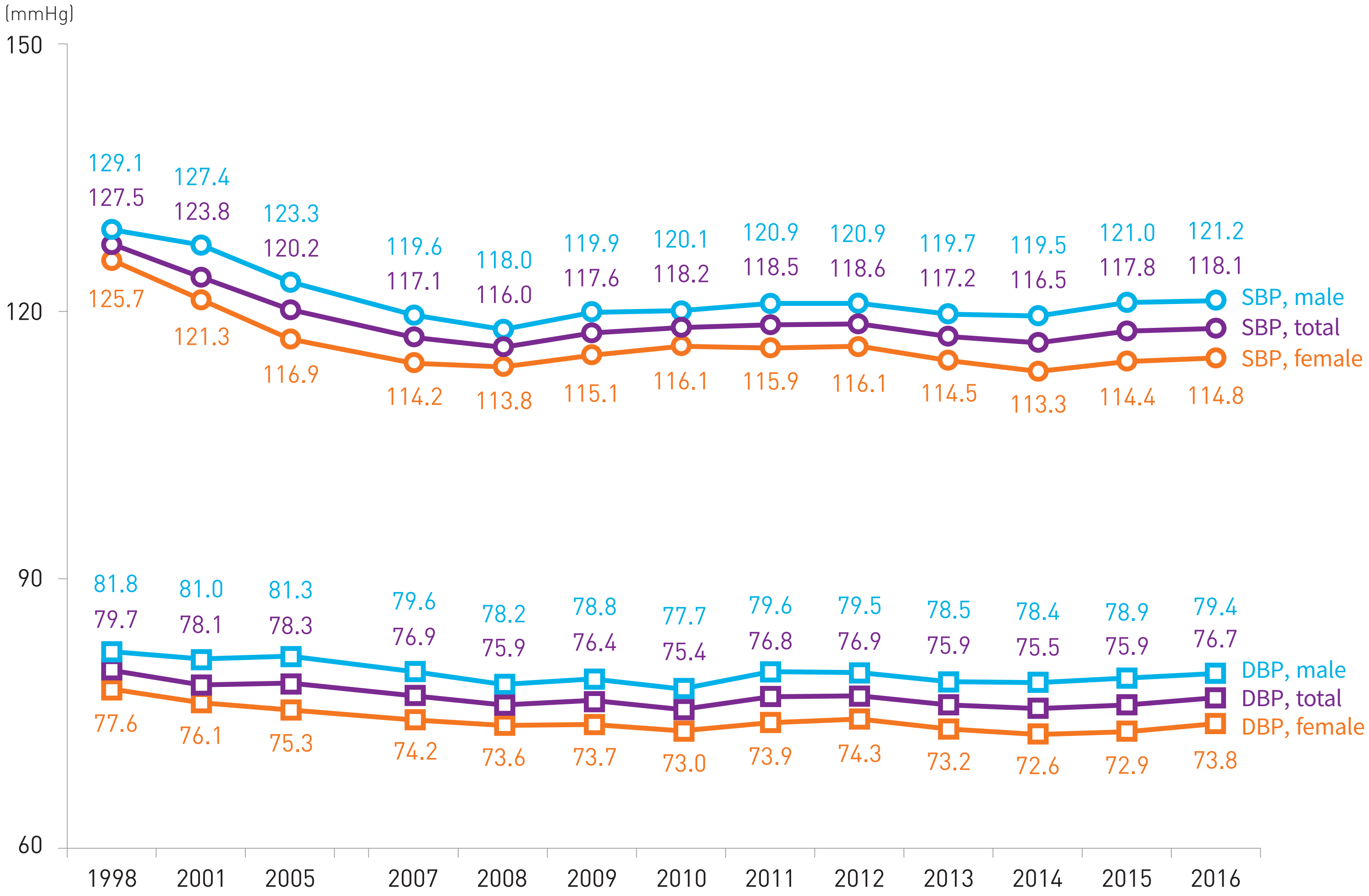

Data source: KNHANES (1998–2016)

Changes in prevalence

(Age 30+, age-standardized)

29%

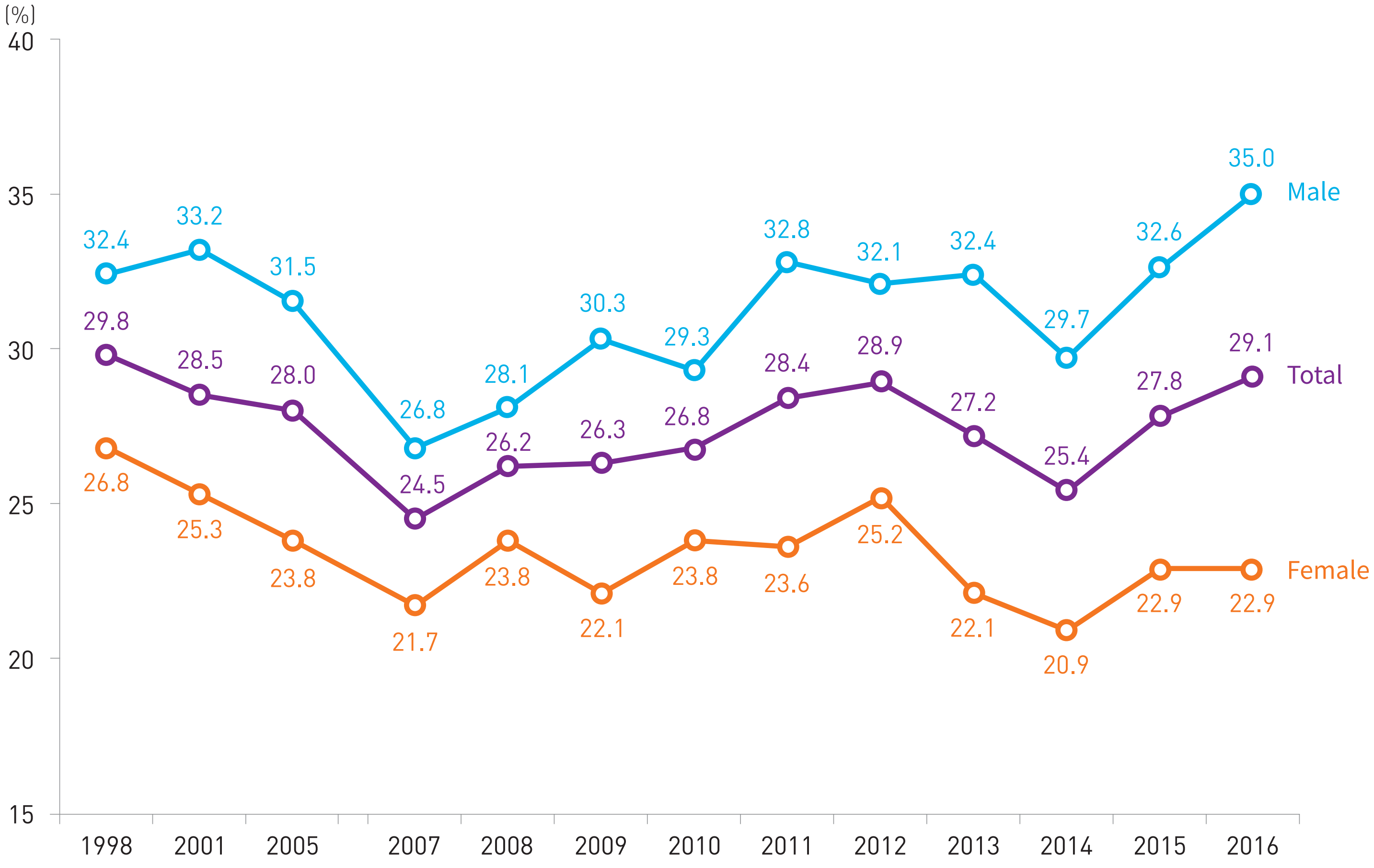

Data source: KNHANES (1998–2016)

Changes in prevalence by sex and age

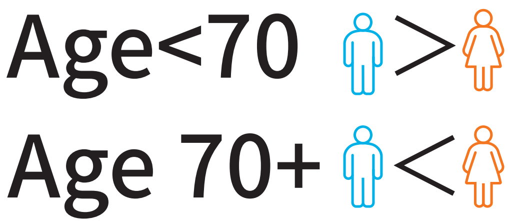

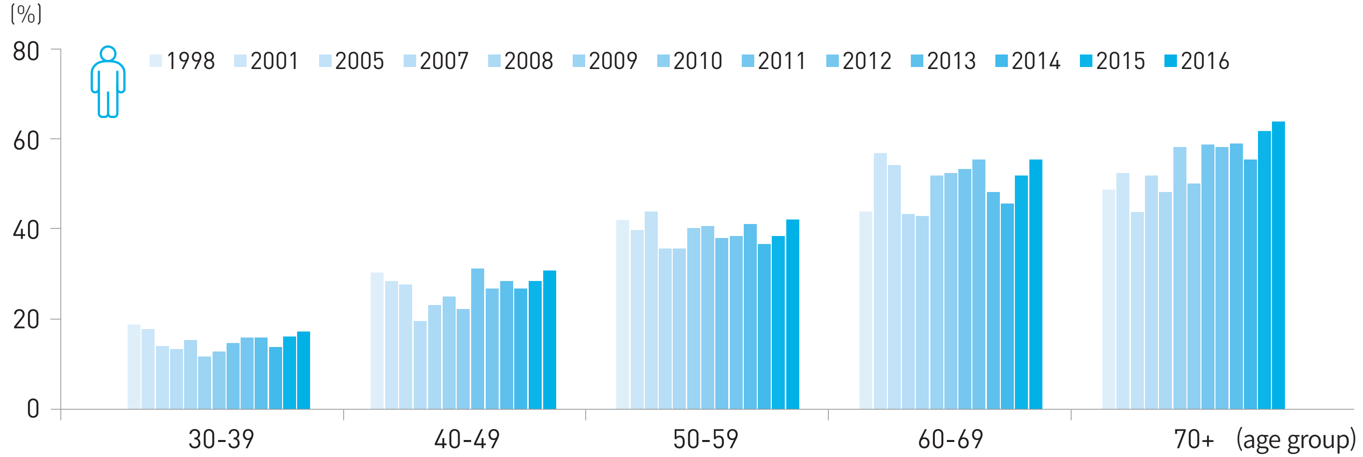

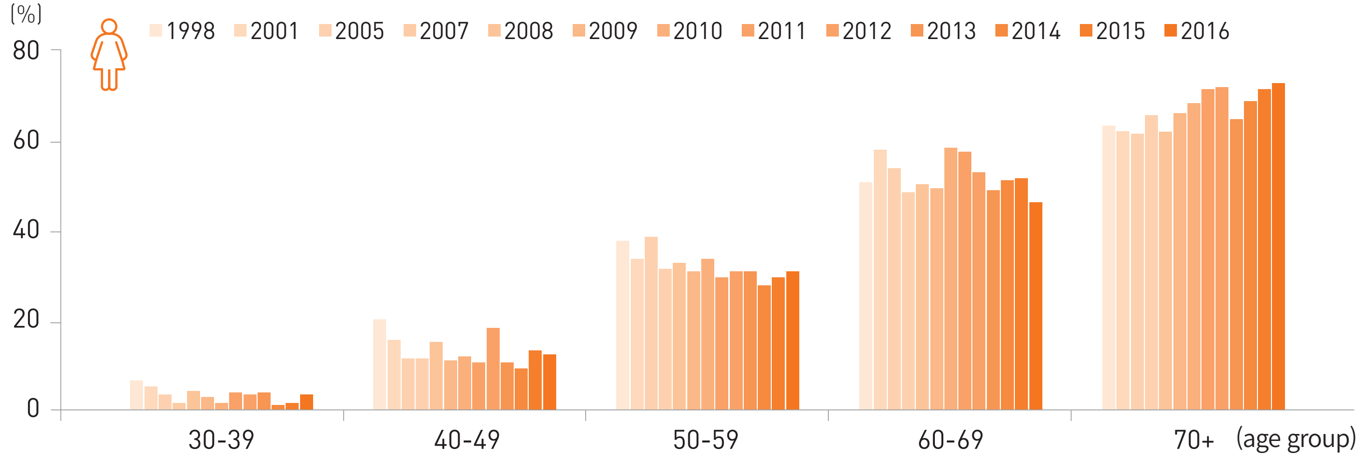

Data source: KNHANES (1998–2016)

Changes in the estimated number of people with hypertension

Now exceeding 11 million

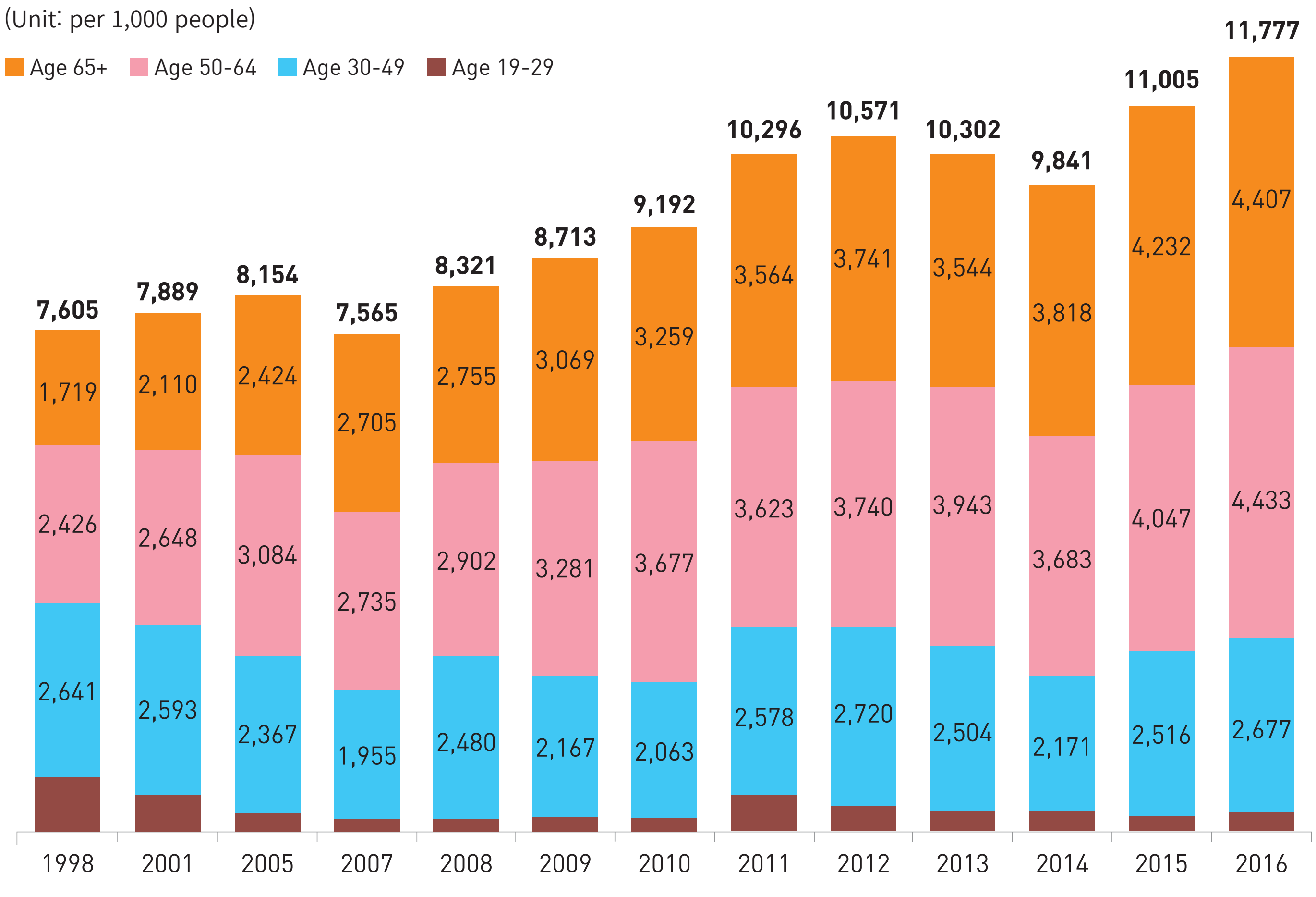

Data source: KNHANES (1998-2016)

2 Changes in indicators of hypertension management

Data source: KNHANES

Prevalence and management indicators by sex

(Age 30+)

56%

Uncontrolled

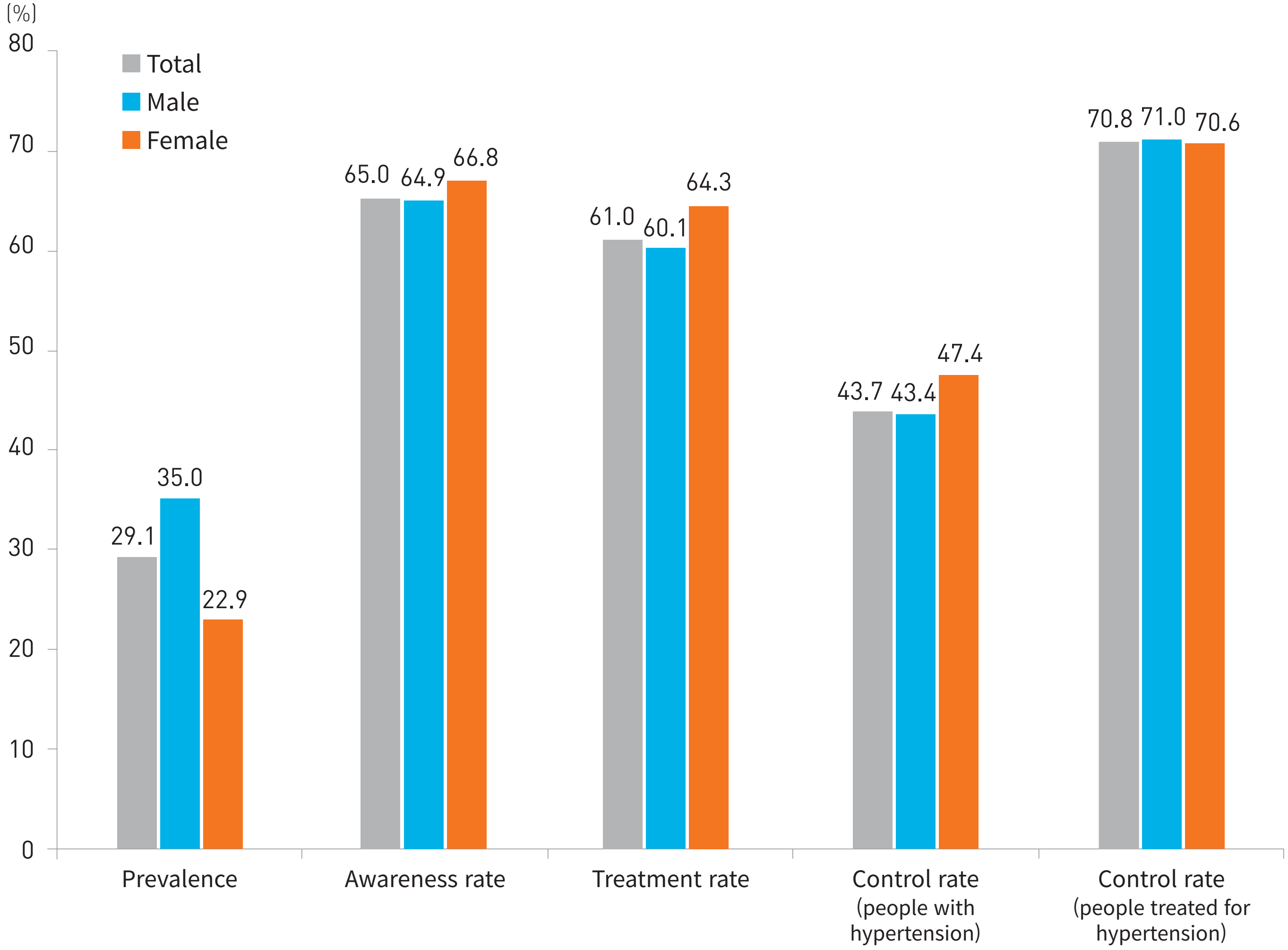

Data source: KNHANES (2016)

Changes in management indicators

(Age 30+, age-standardized)

Rapid improvement between '98-'07
Stagnant in the past 10 years

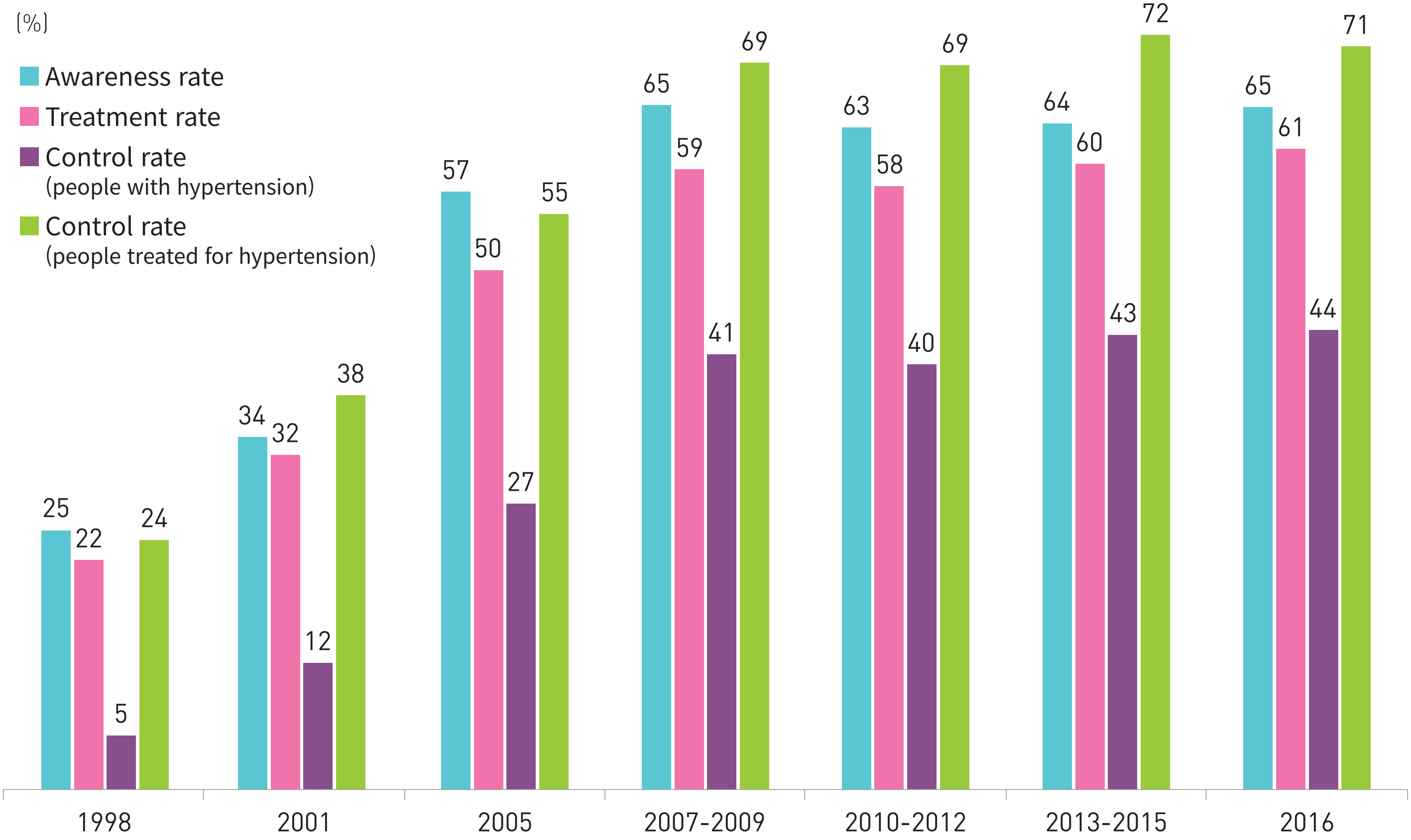

Data source: KNHANES (1998-2016)

Changes in awareness rate by sex

(Age 30+, age-standardized)

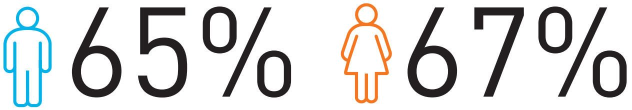

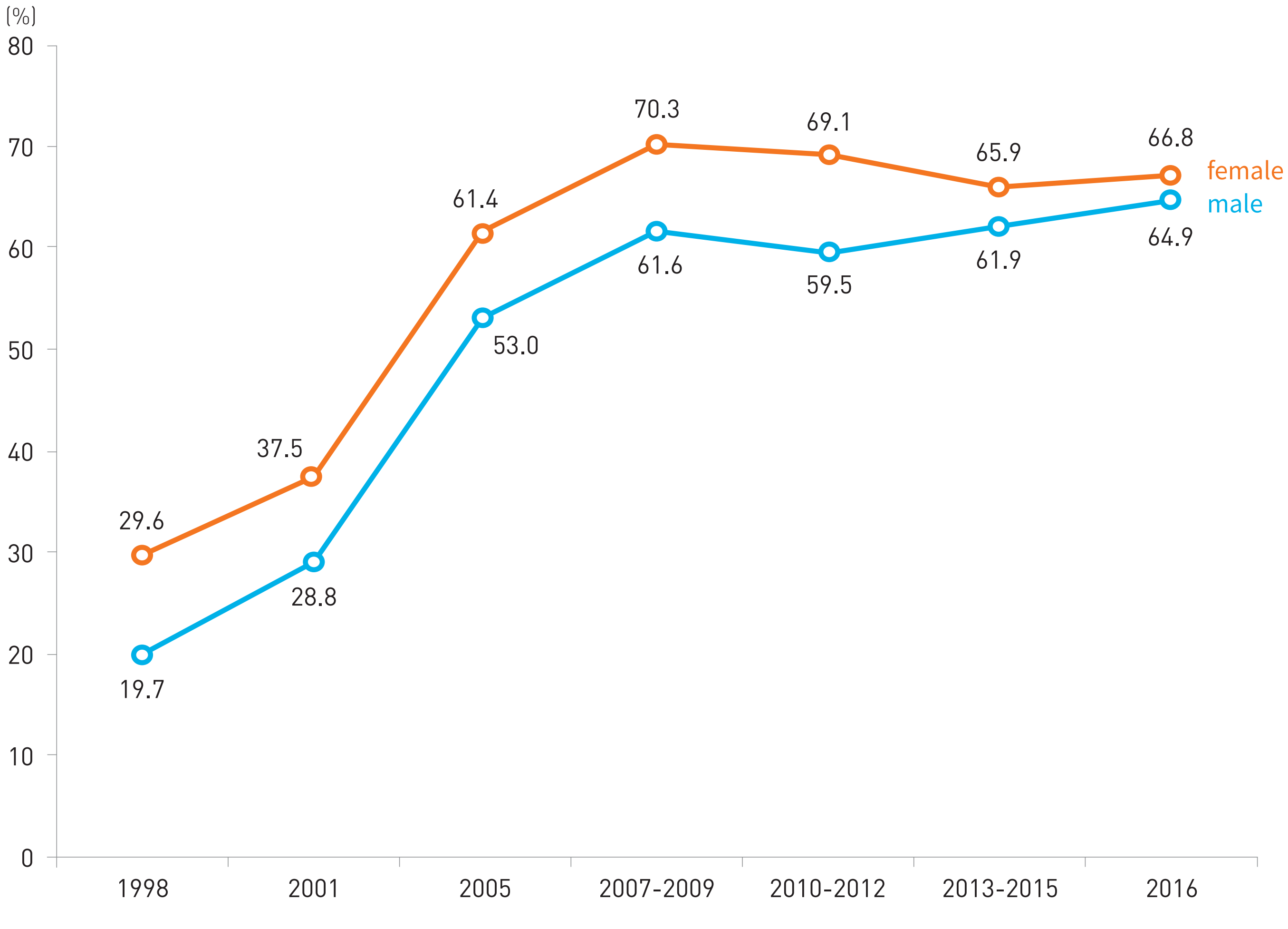

Data source: KNHANES (1998-2016)

Changes in treatment rate by sex

(Age 30+, age-standardized)

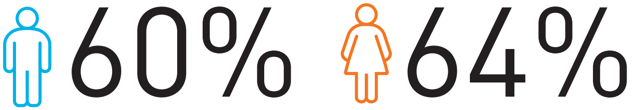

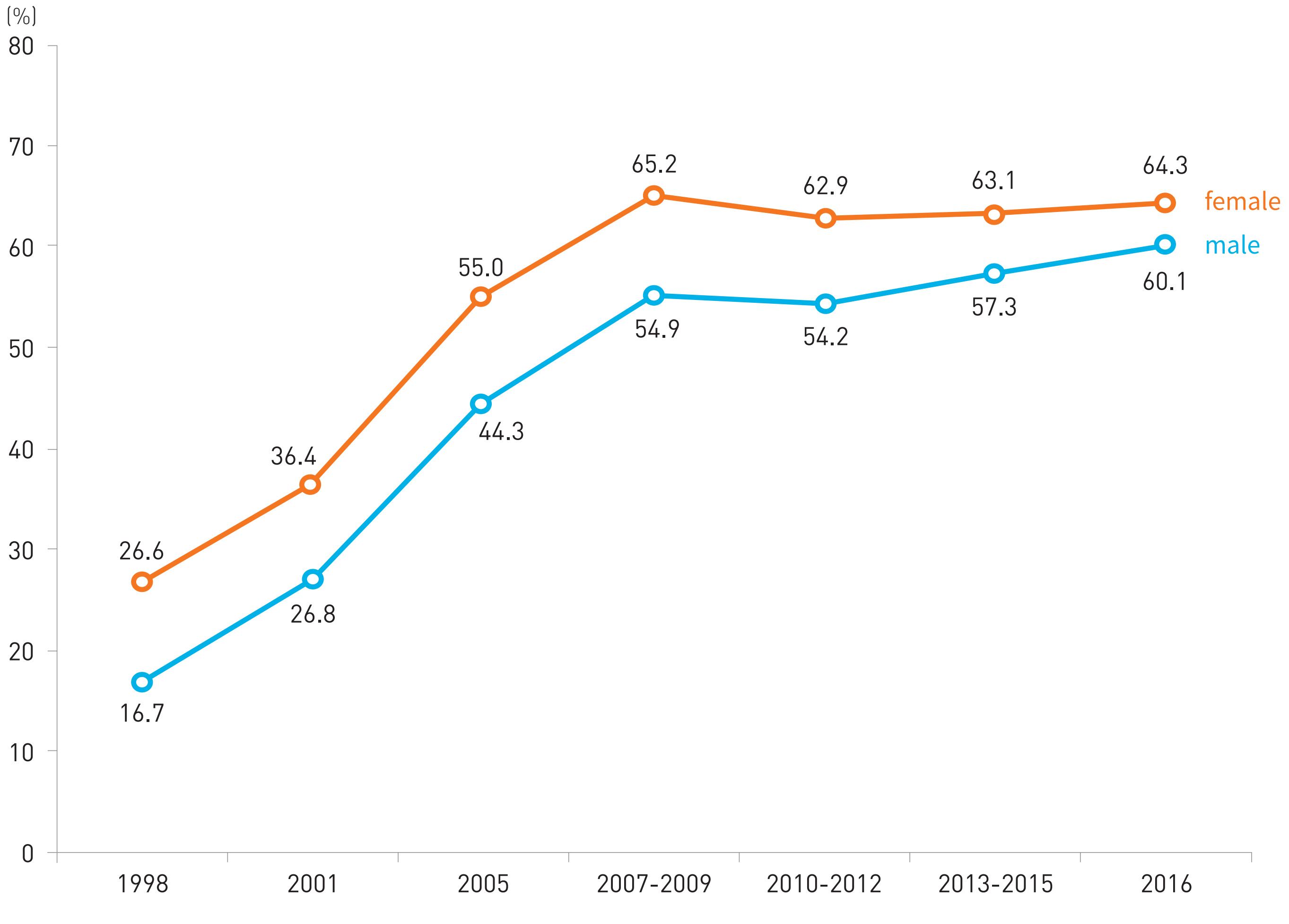

Data source: KNHANES (1998–2016)

Changes in control rate among people with hypertension by sex

(Age 30+, age-standardized)

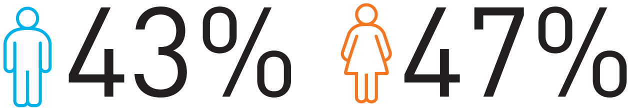

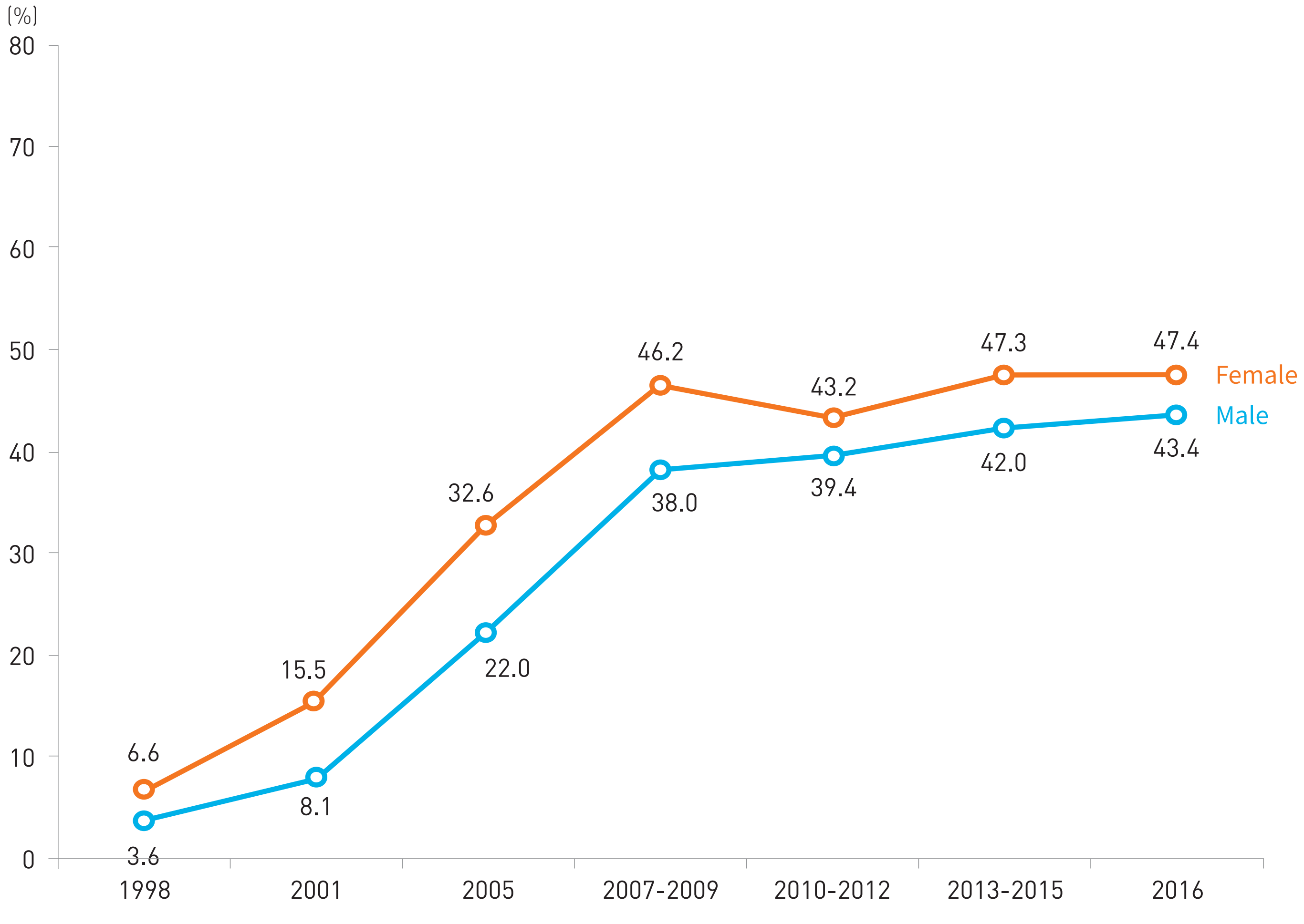

Data source: KNHANES (1998-2016)

Changes in control rate among people treated for hypertension by sex

(Age 30+, age-standardized)

71% 71%

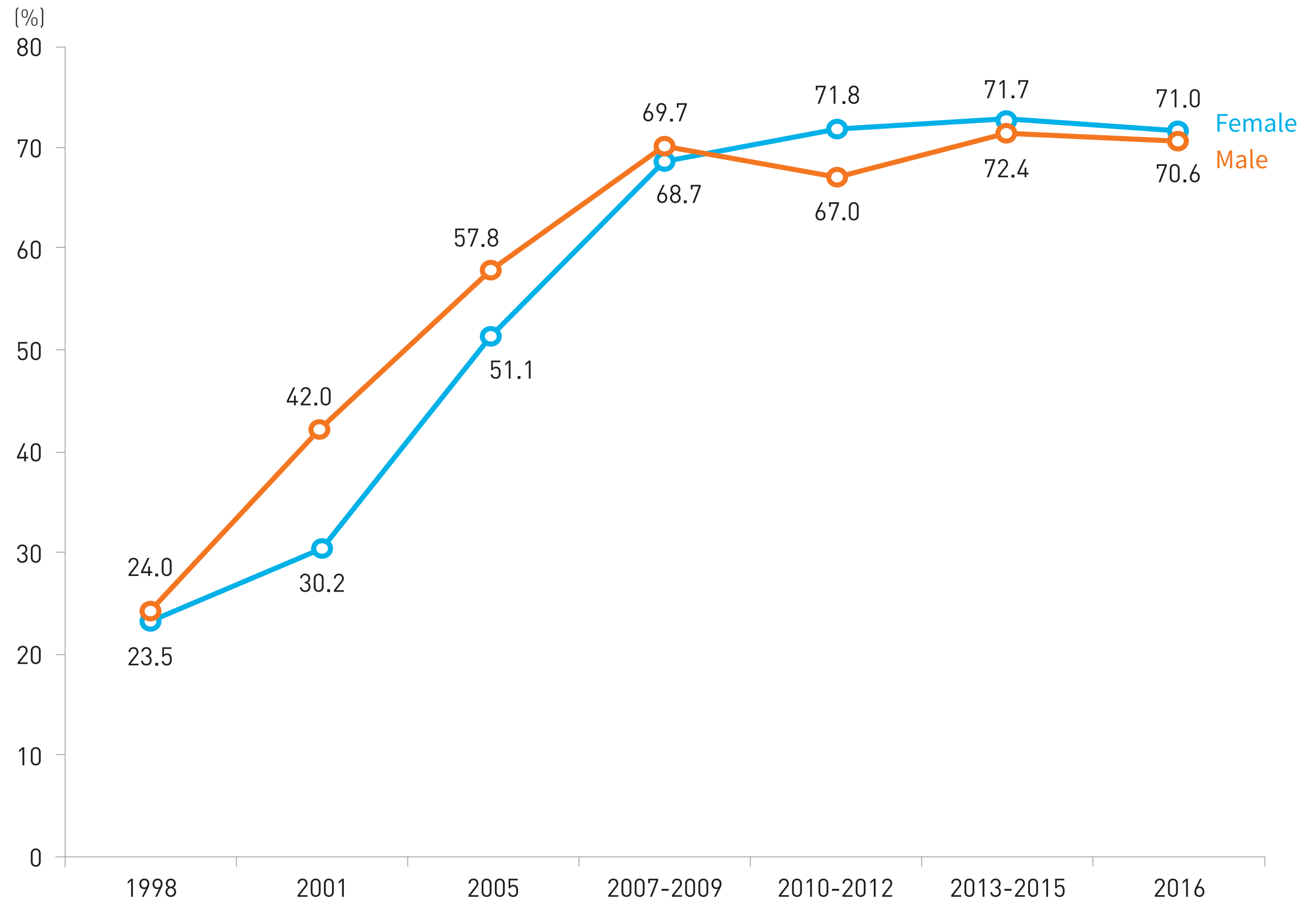

Data source: KNHANES (1998–2016)

Changes in awareness rate by sex and age

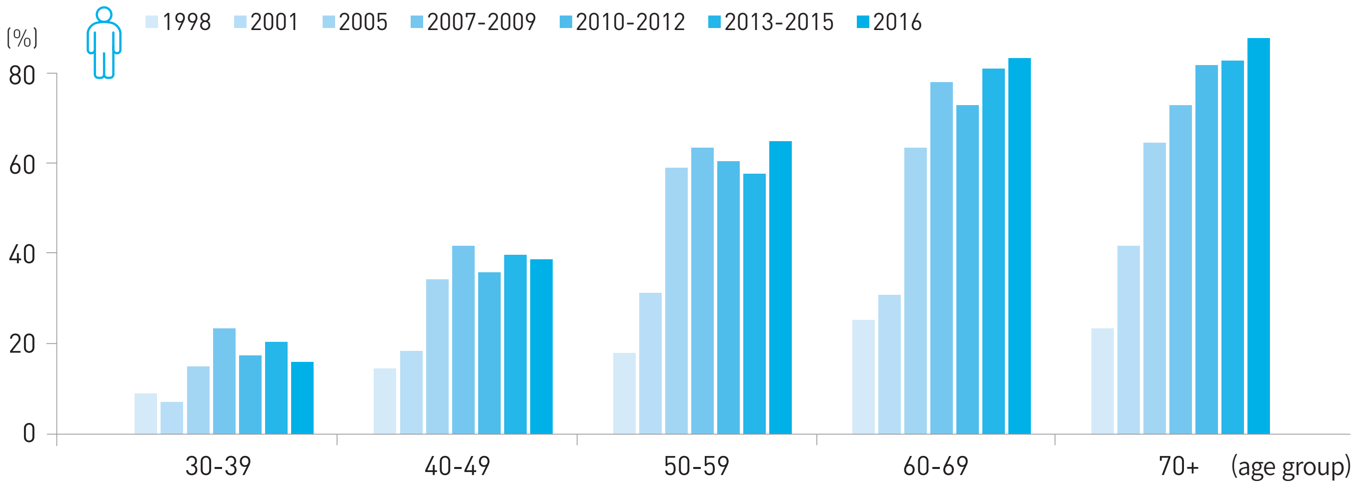

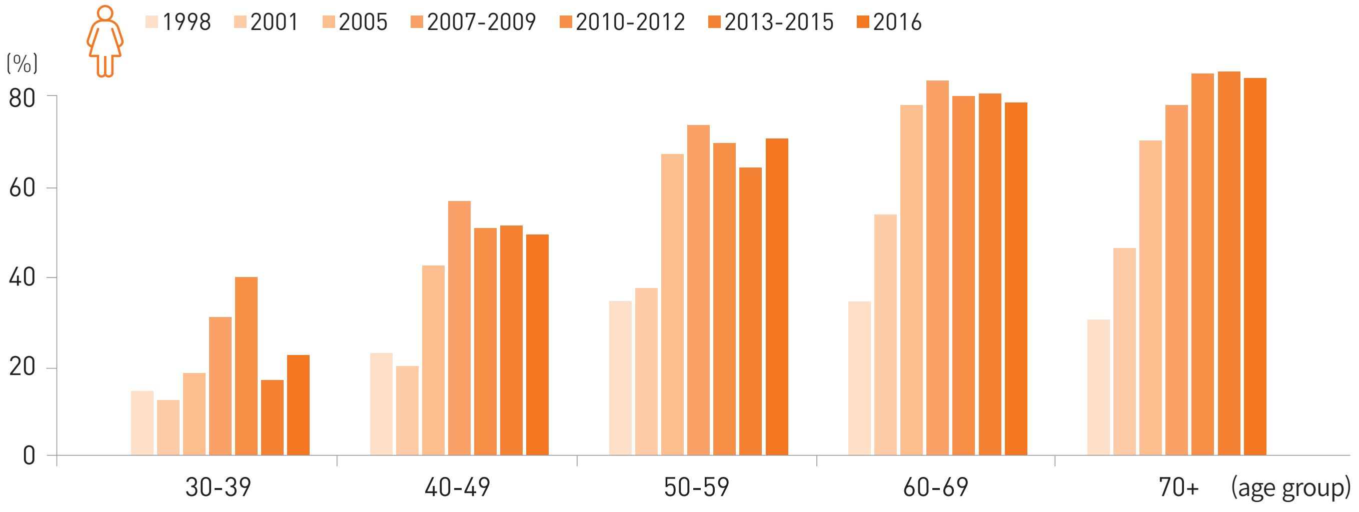

 Data source: KNHANES (1998-2016)

Changes in treatment rate by sex and age

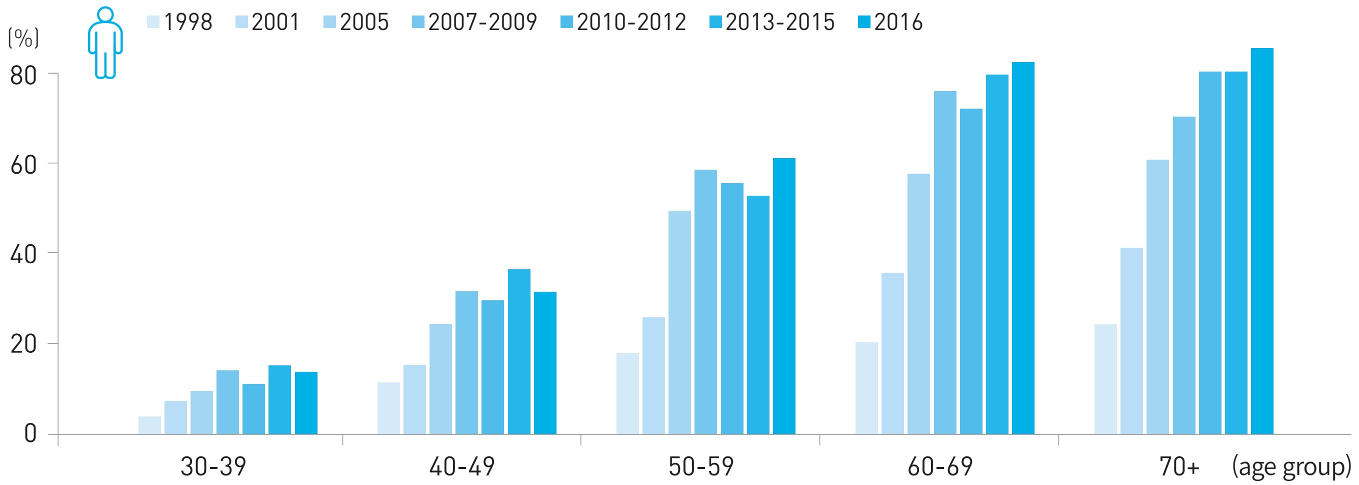

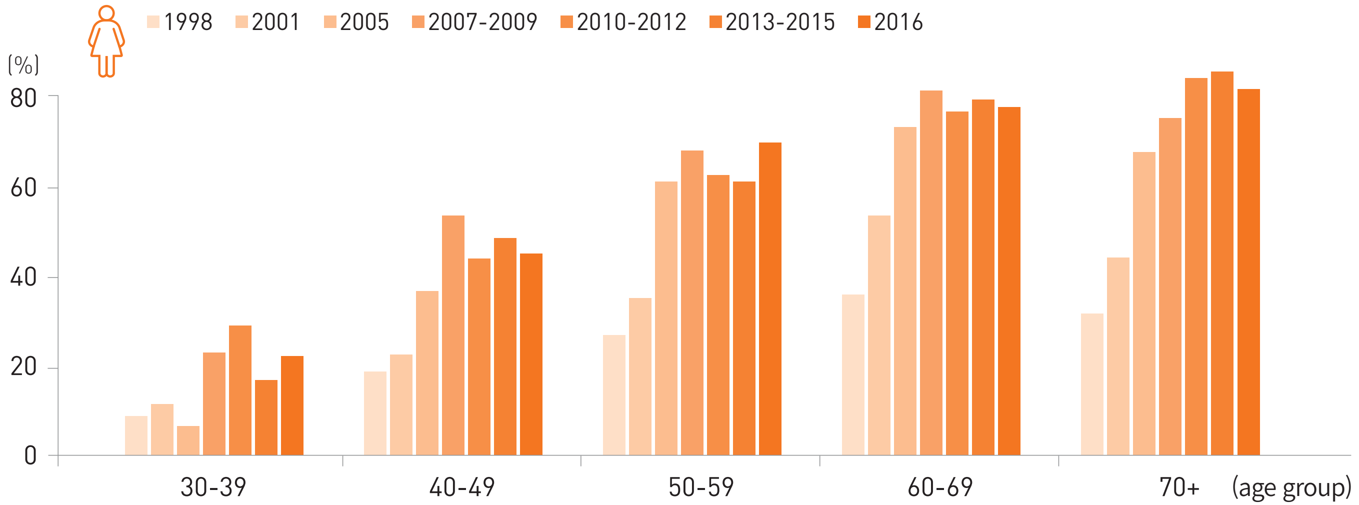

Data source: KNHANES (1998–2016)

Changes in control rate among people with hypertension by sex and age

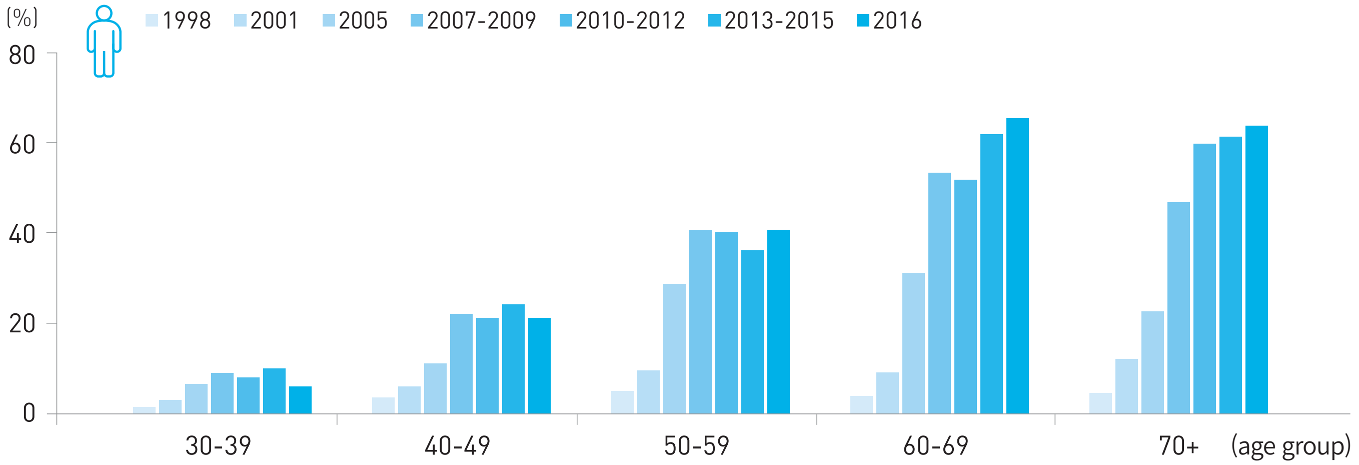

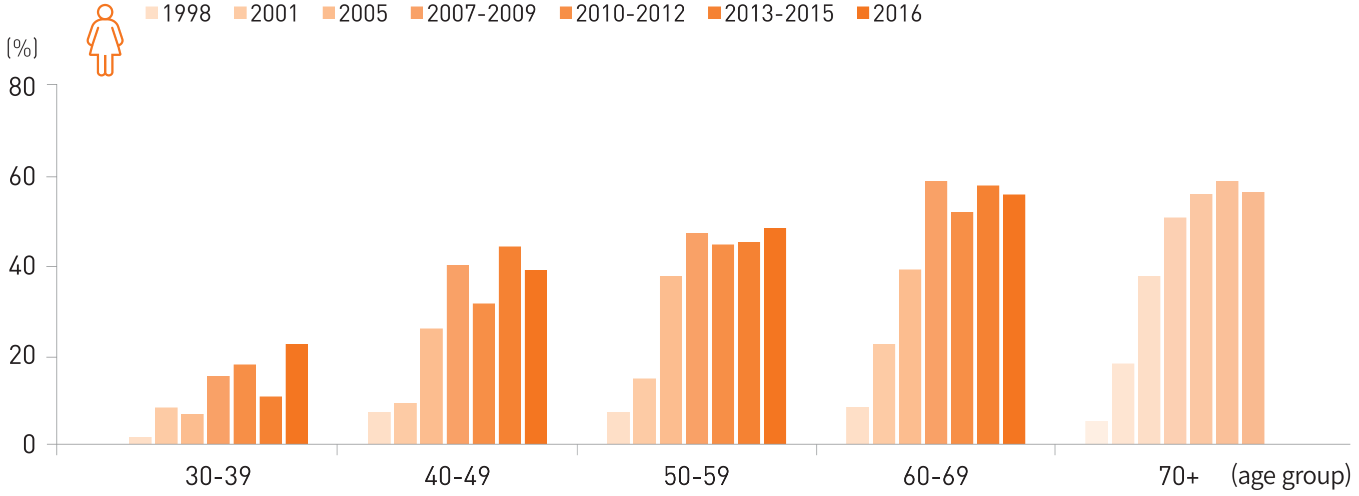

Data source: KNHANES (1998-2016)

Changes in control rate among people treated for hypertension by sex and age

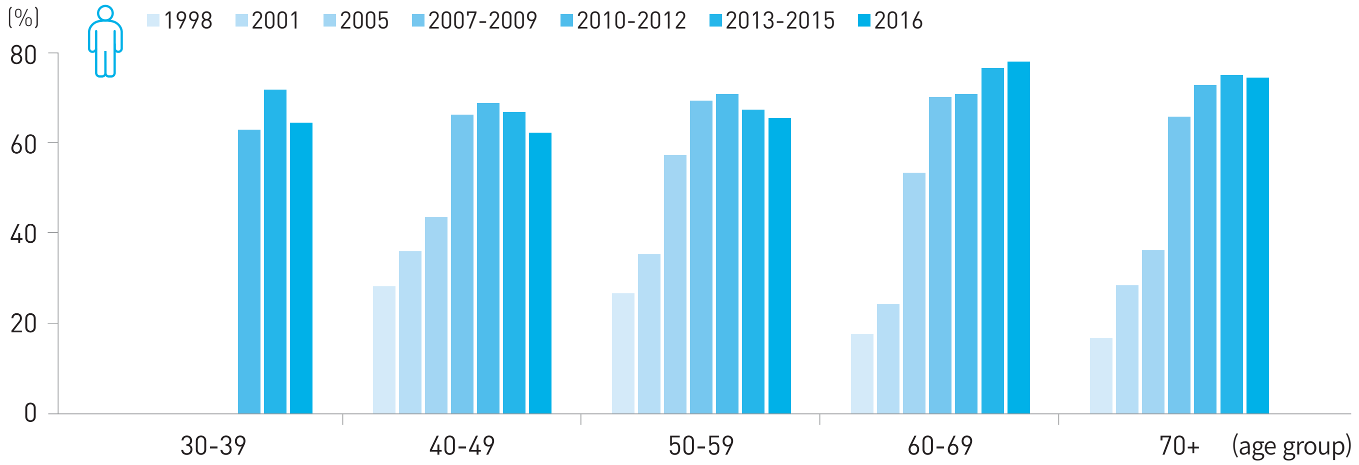

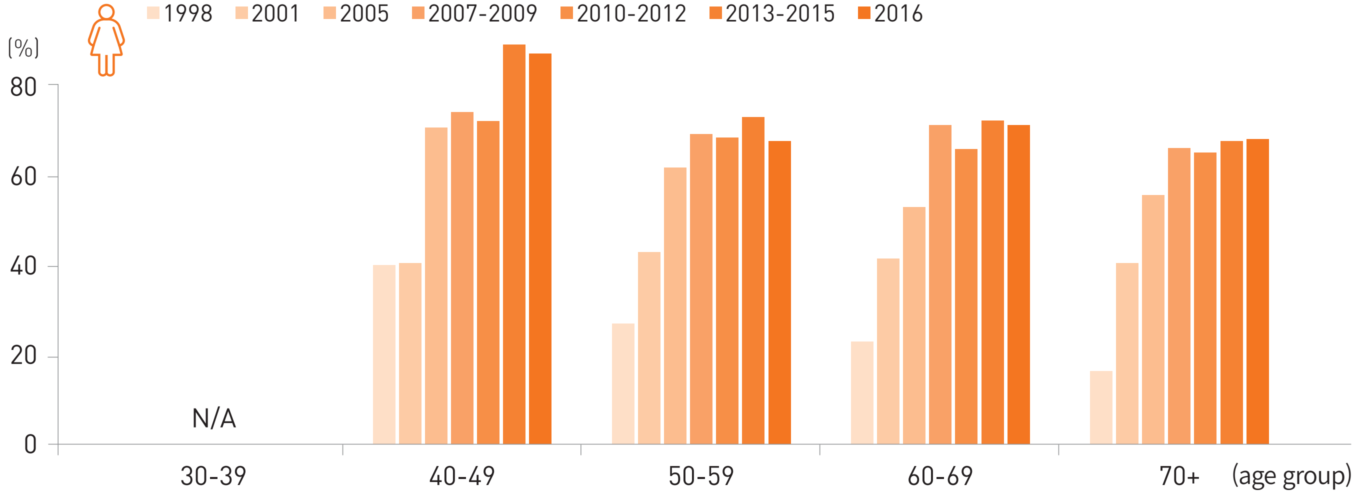

Data source: KNHANES (1998–2016)

3 Healthcare services utilization for hypertension

Data source: NHIBD

Changes in healthcare services utilization and treatment

Healthcare service users **8,921,829** people

Receiving treatment **8,219,104** people

Persistent treatment **5,734,238** people

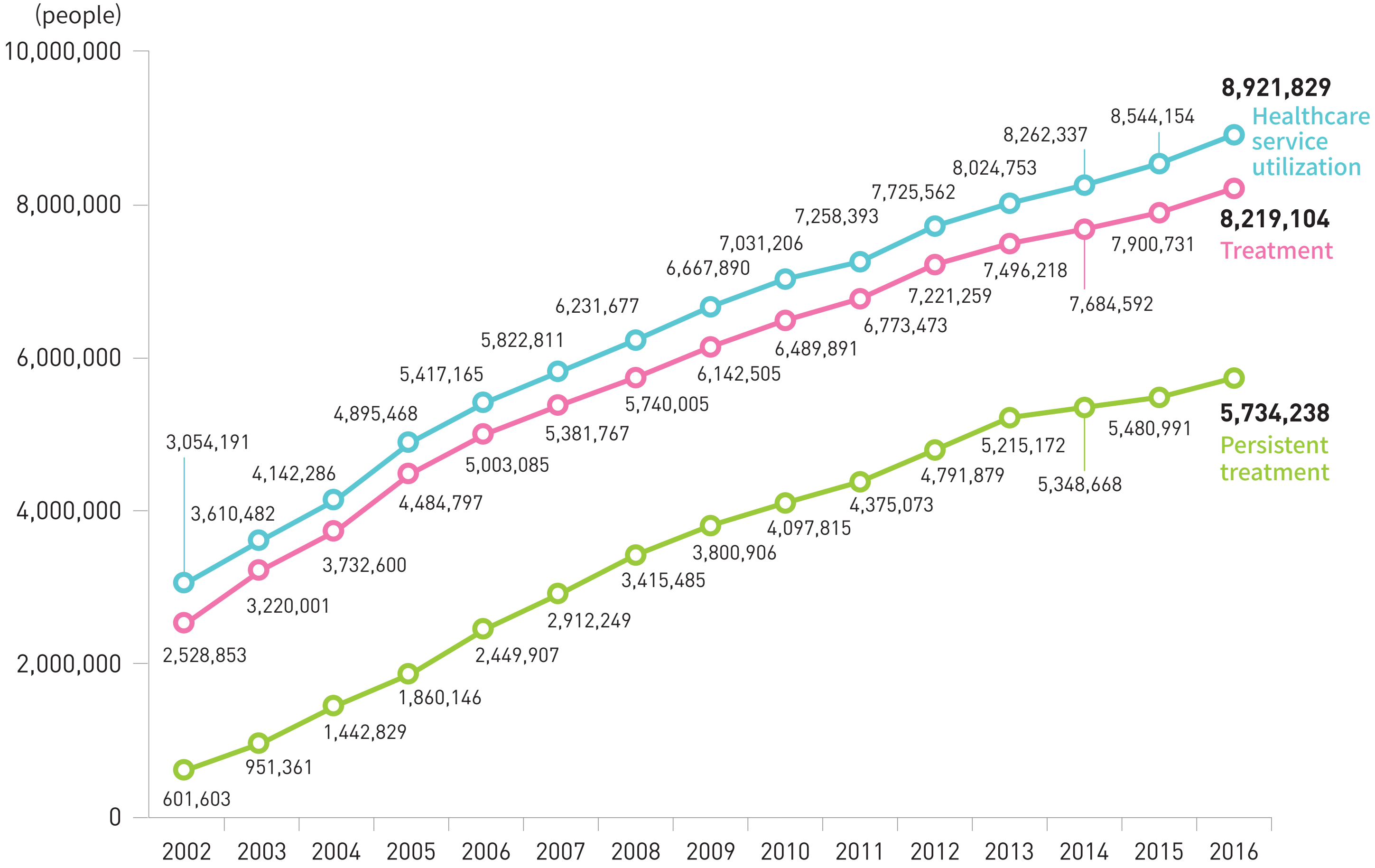

Data source: NHIBD (2002-2016)

Age distribution of people treated for hypertension

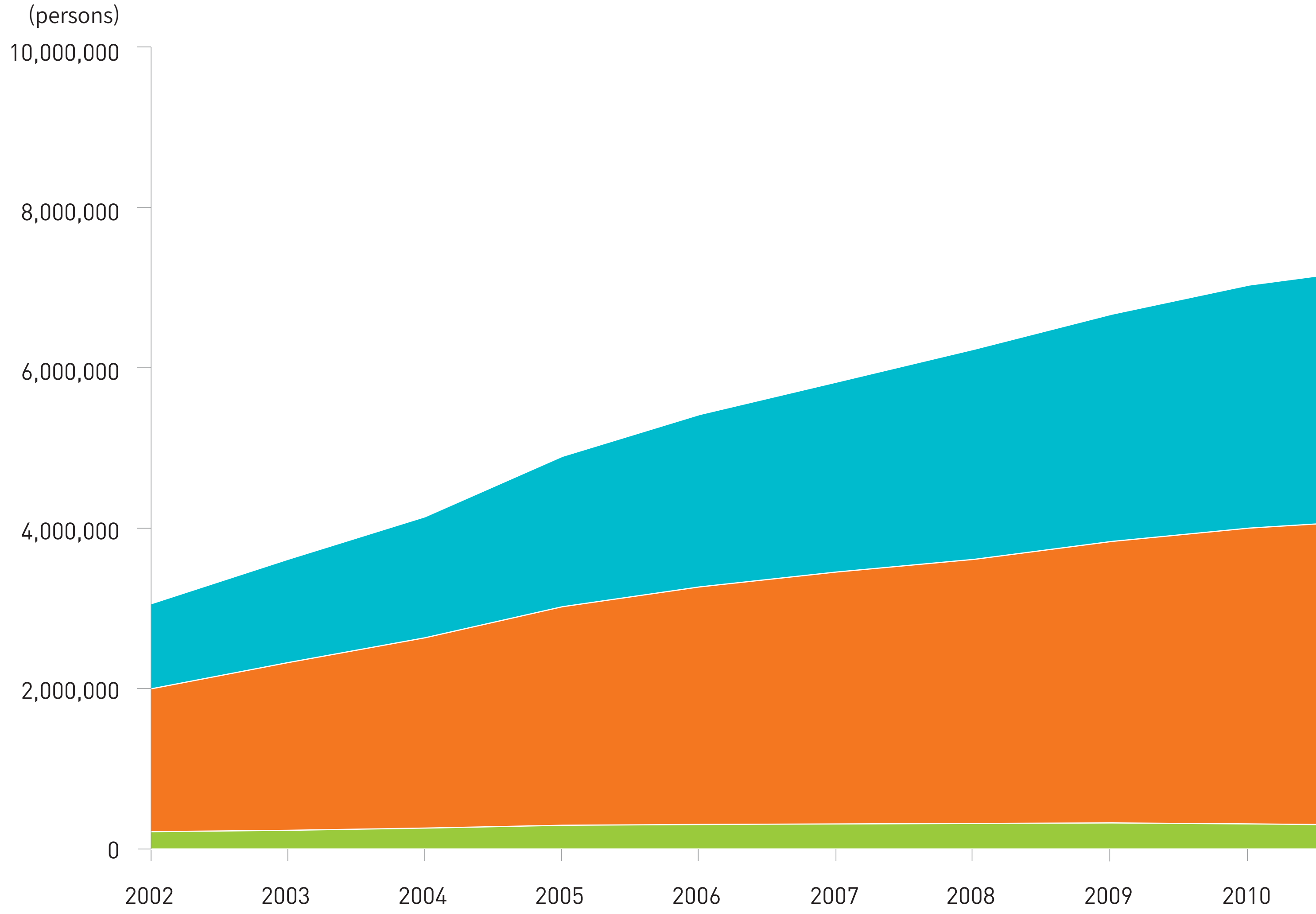

34%→46% | Elderly (age 65+) people with hypertension

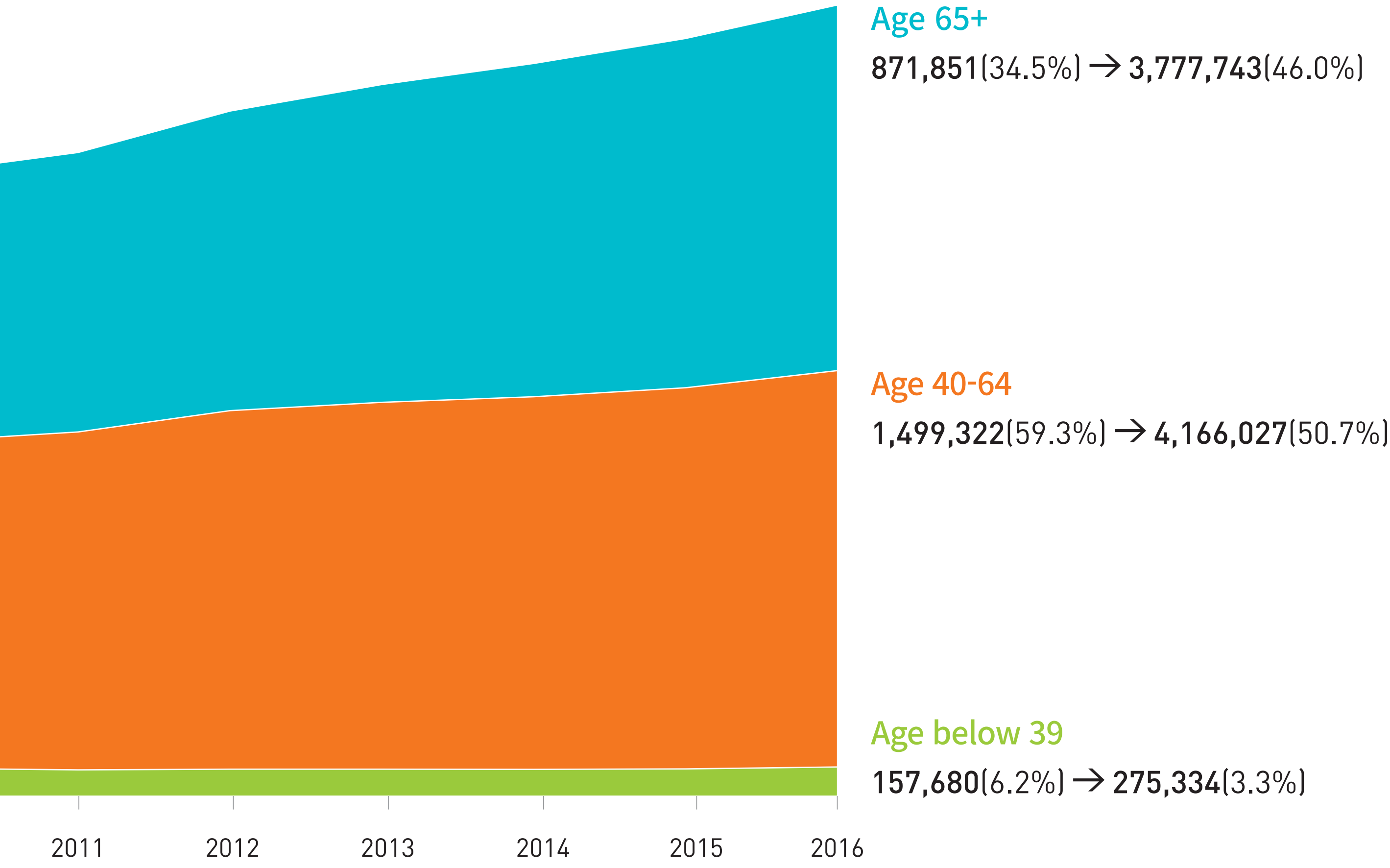

Data source: NHIBD (2002-2016)

Co-treatment for hypertension, dyslipidemia, or diabetes

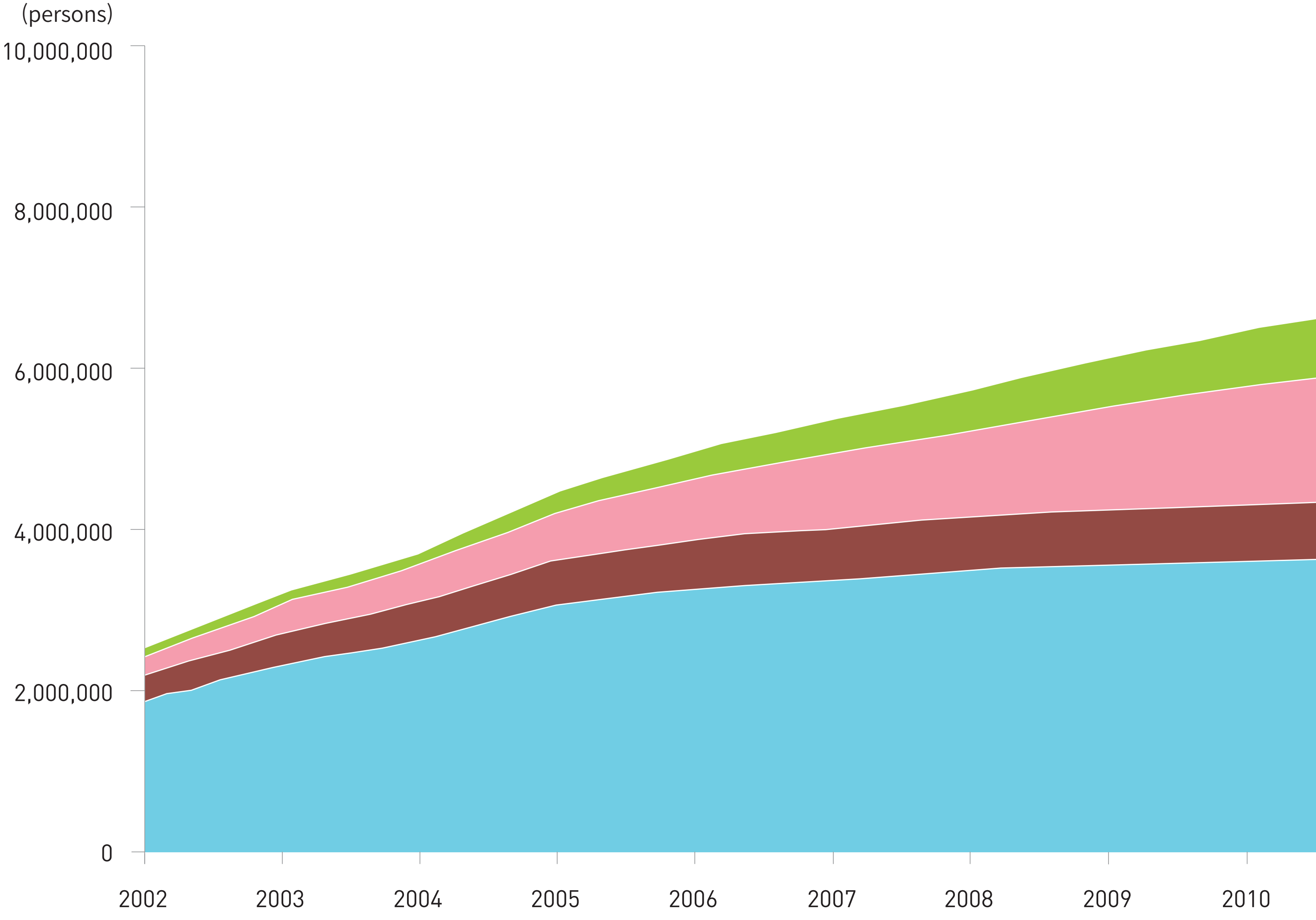

25%→57%

People who are also treated for diabetes or dyslipidemia

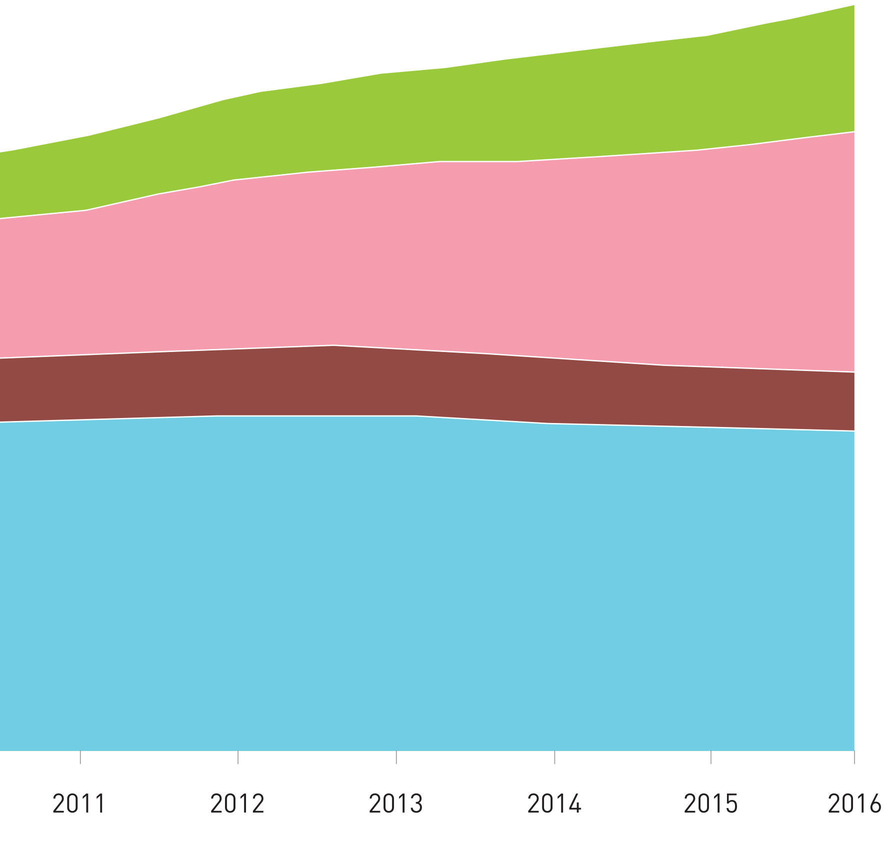

Hypertension+Dyslipidemia+Diabetes
92,771(3.7%) → **1,407,011**(17.1%)

Hypertension+Dyslipidemia
229,540(9.1%) → **2,621,509**(31.9%)

Hypertension+Diabetes
318,813(12.6%) → **638,706**(7.8%)

Hypertension only
1,887,729(74.6%) → **3,551,878**(43.2%)

Data source: NHIBD (2002-2016)

Changes in antihypertensive drug prescription

(among all people with treatment)

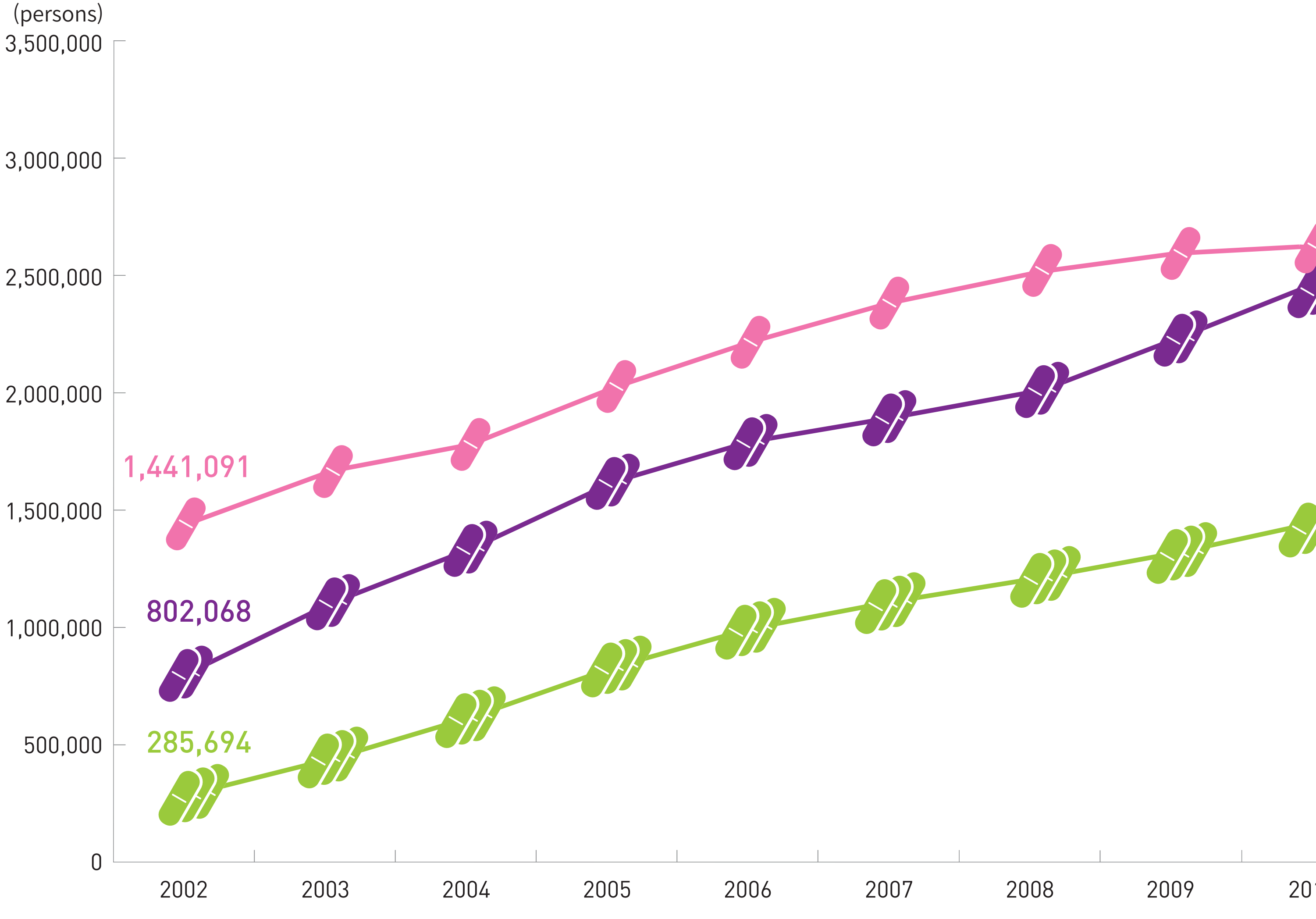

60% are receiving combination therapy

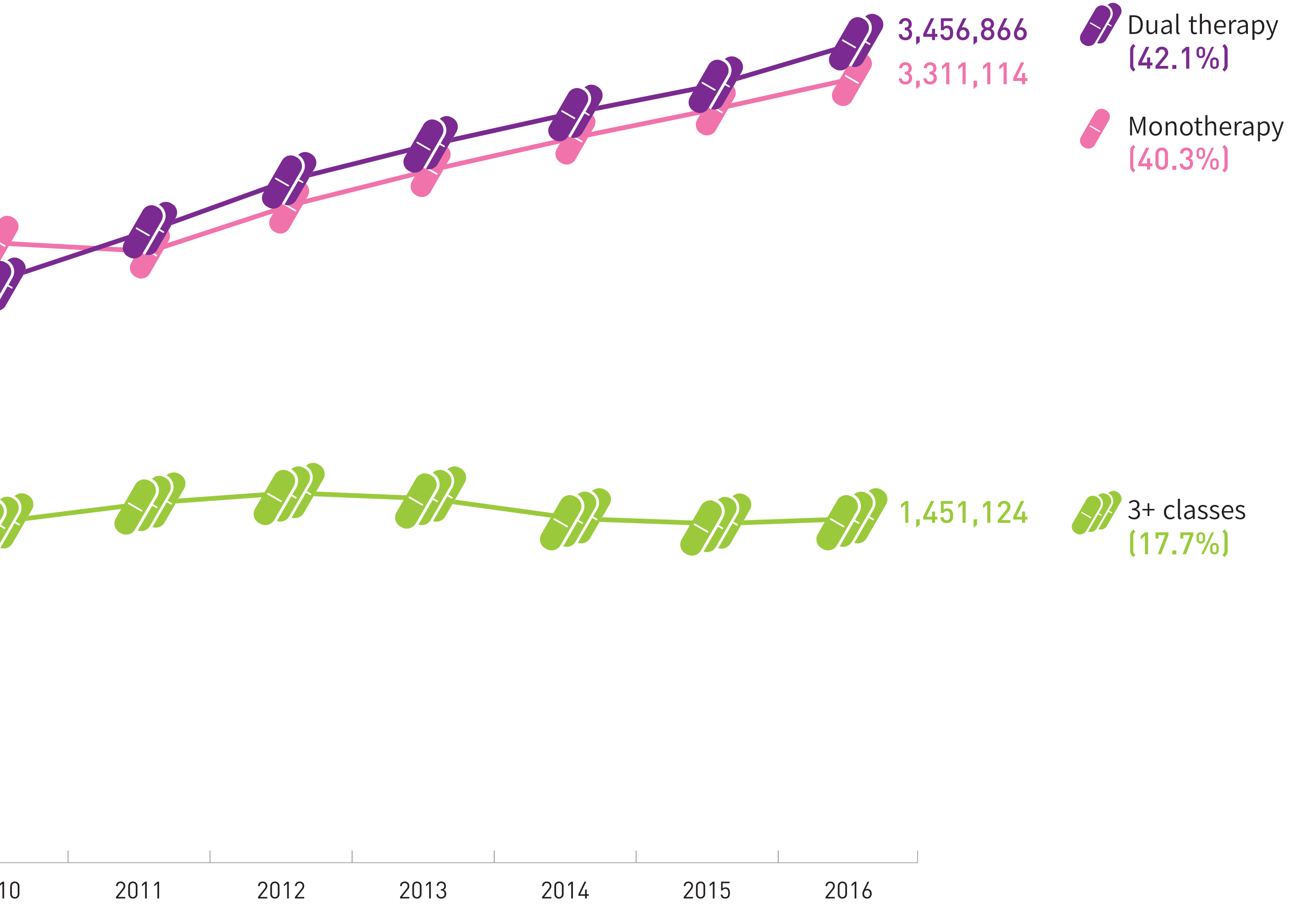

Data source: NHIBD (2002–2016)

Changes in antihypertensive drug prescription

(among people with persistent treatment)

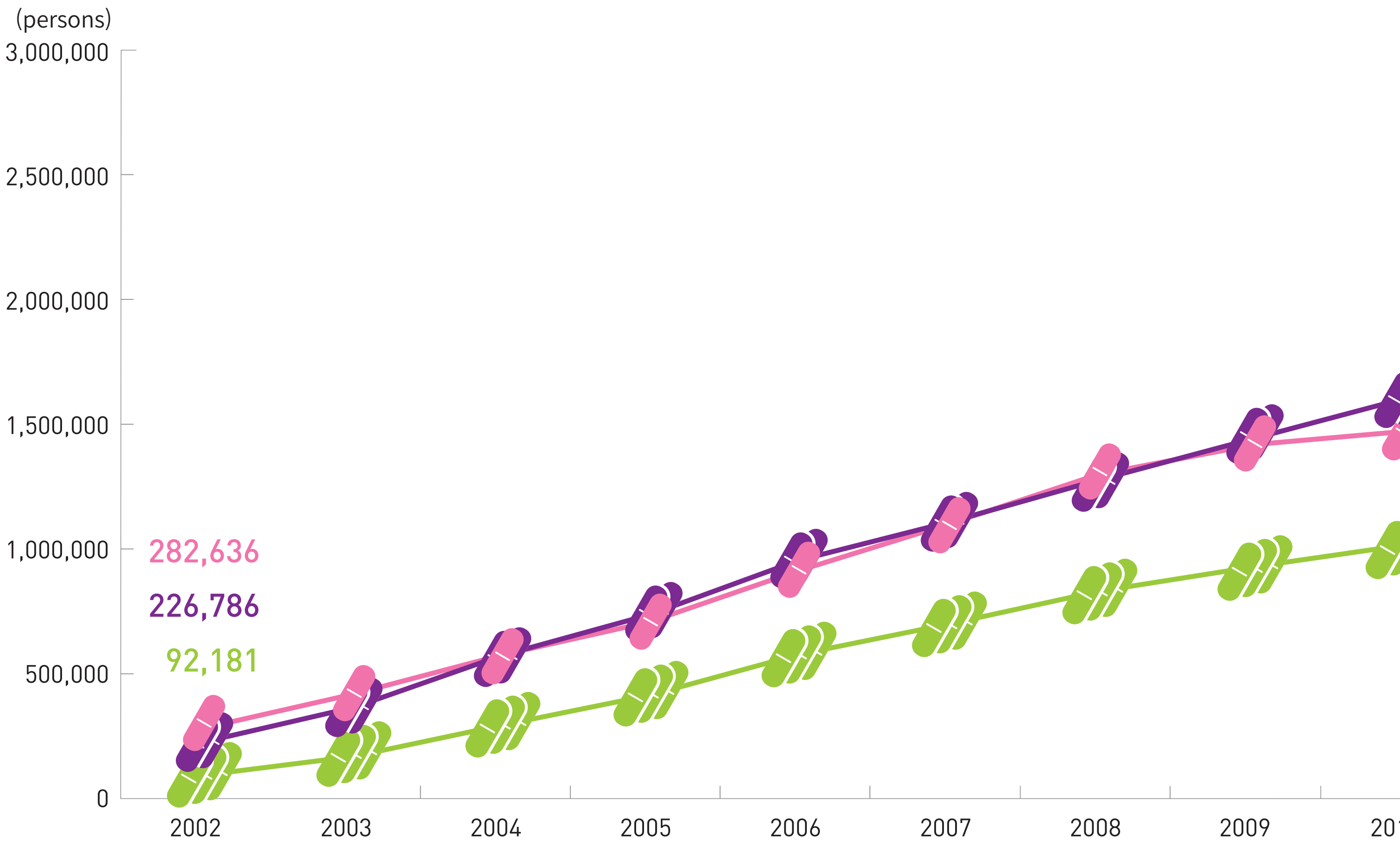

63% are receiving combination therapy

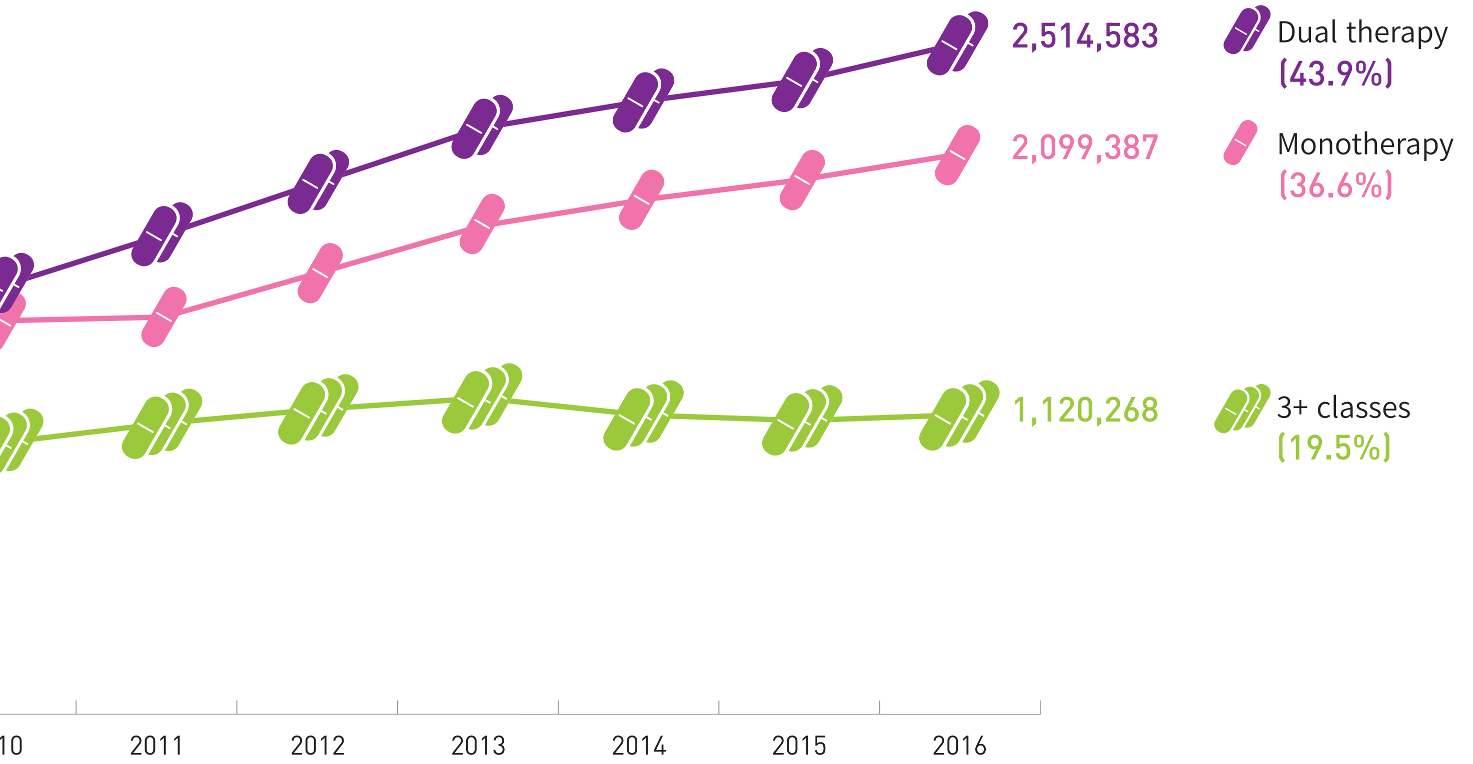

Data source: NHIBD (2002–2016)

Changes in monotherapy composition

(among all people with treatment)

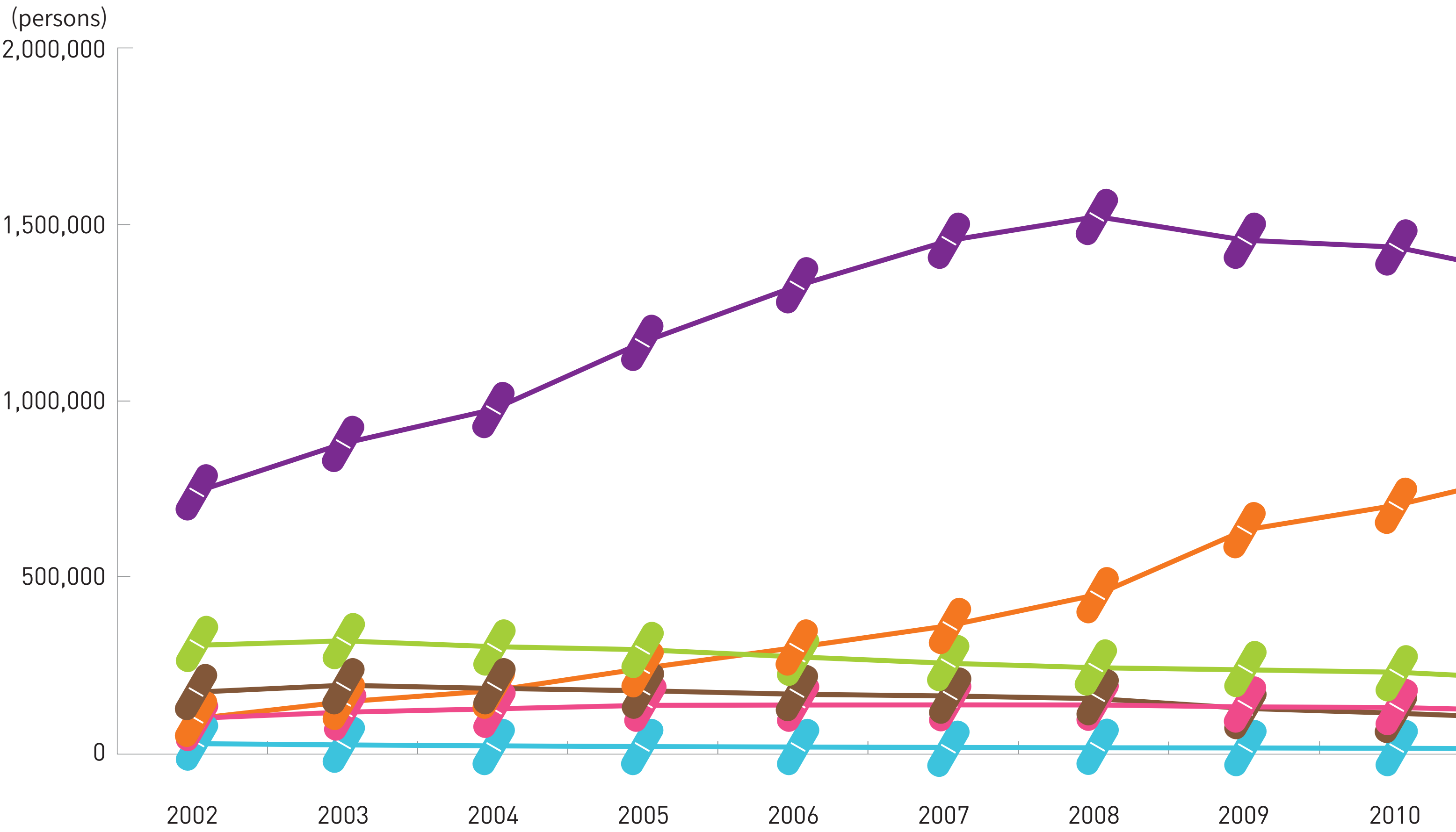

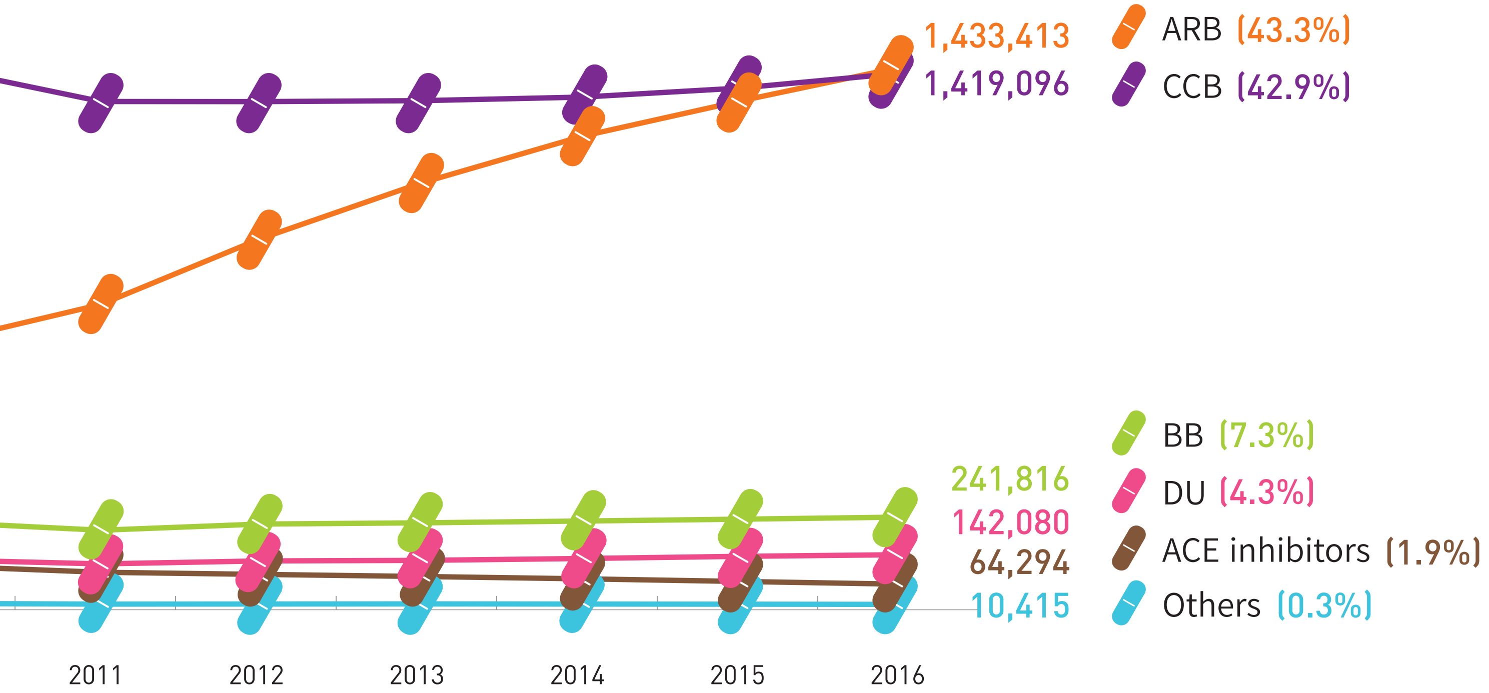

Data source: NHIBD (2002-2016)

Changes in monotherapy composition

(among people with persistent treatment)

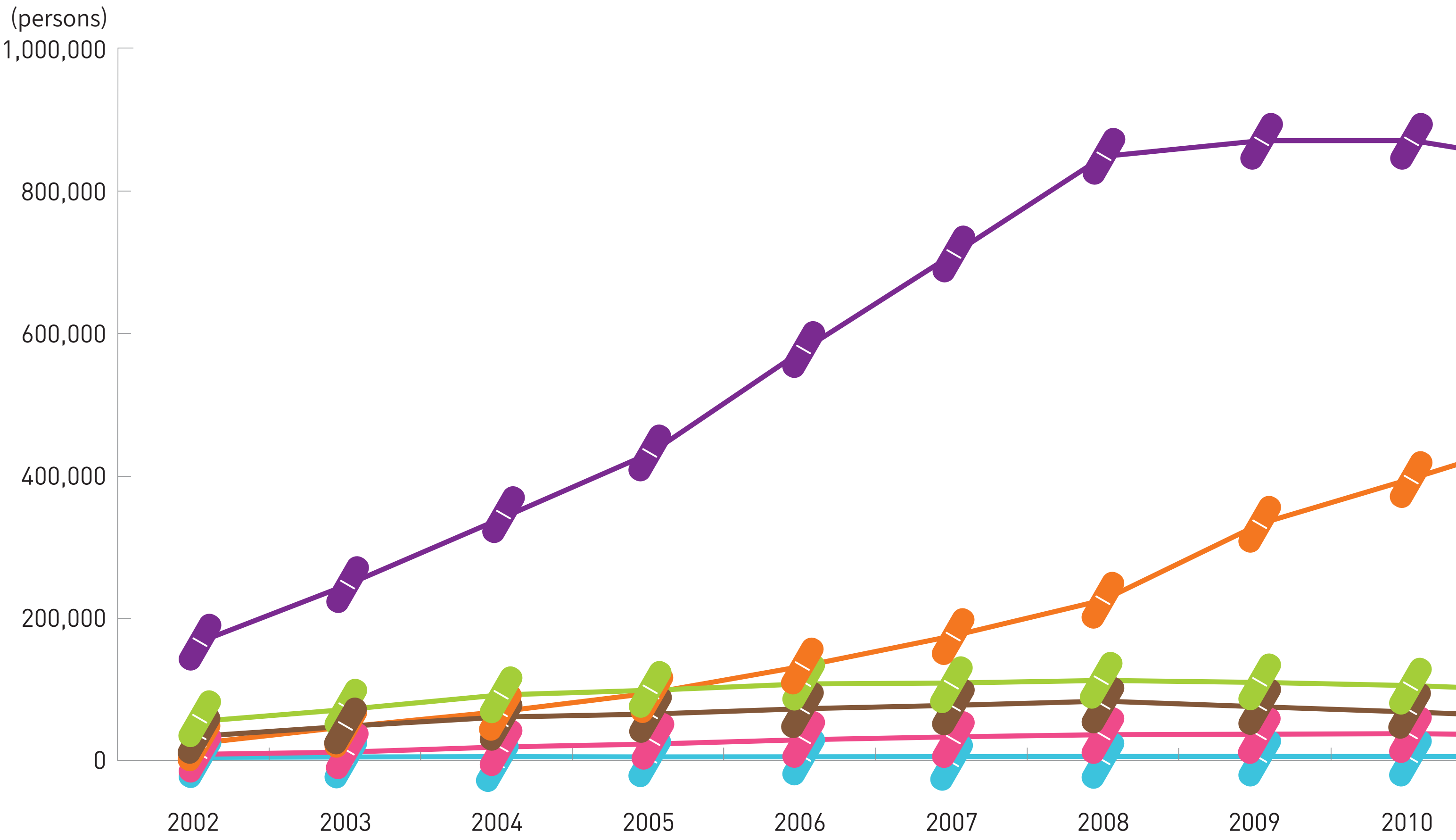

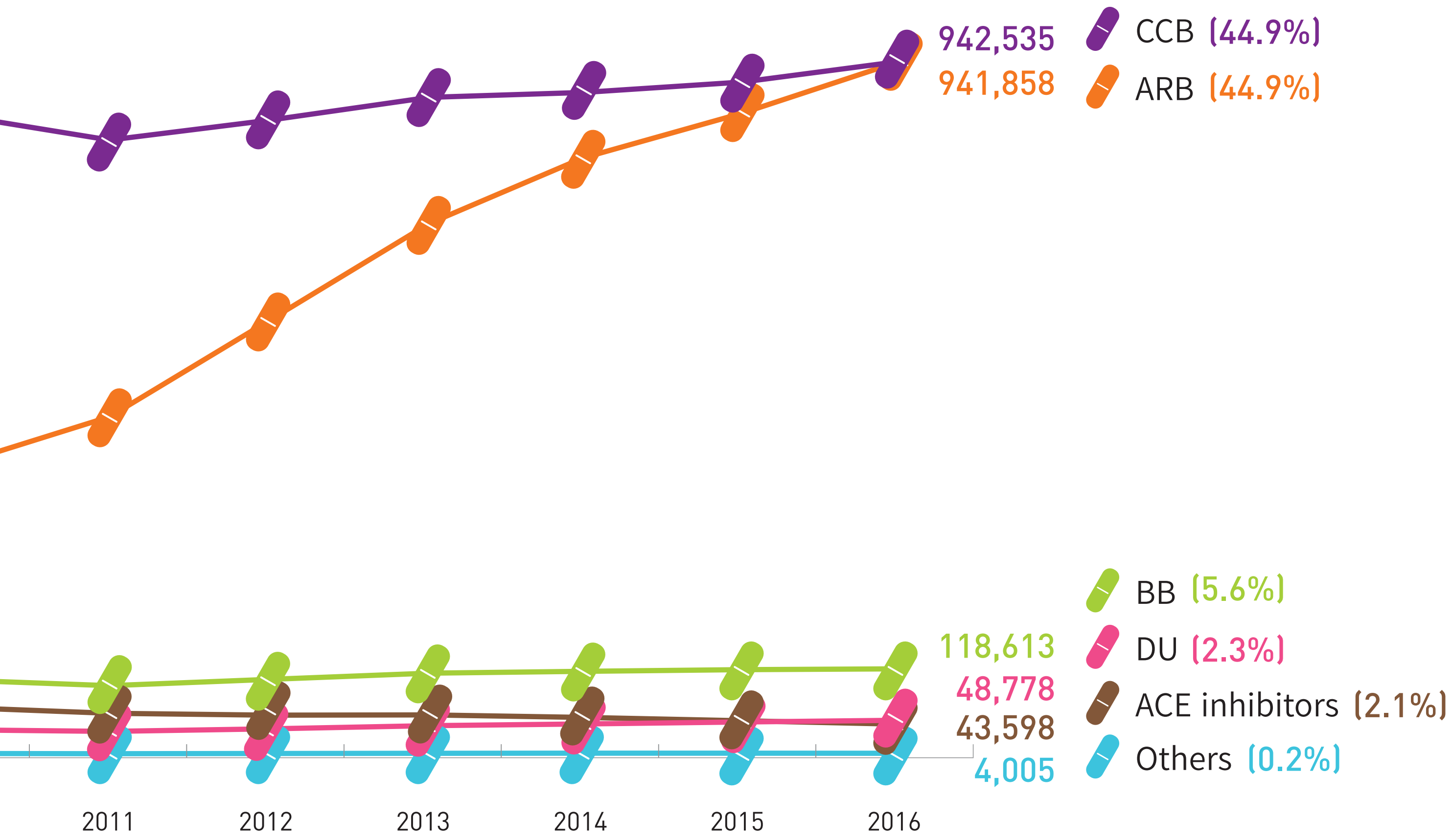

Data source: NHIBD (2002-2016)

Changes in dual therapy composition

(among all people with treatment)

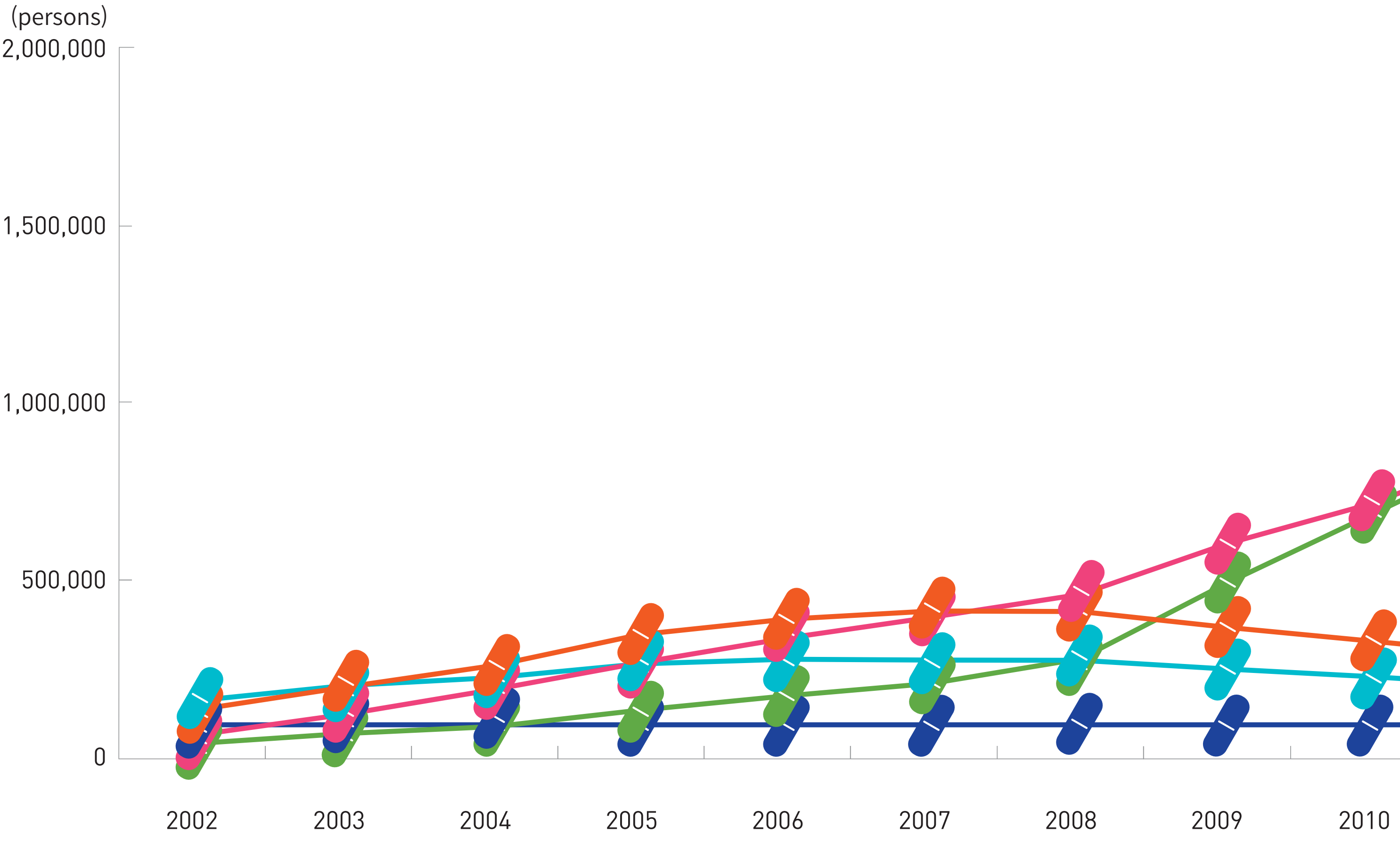

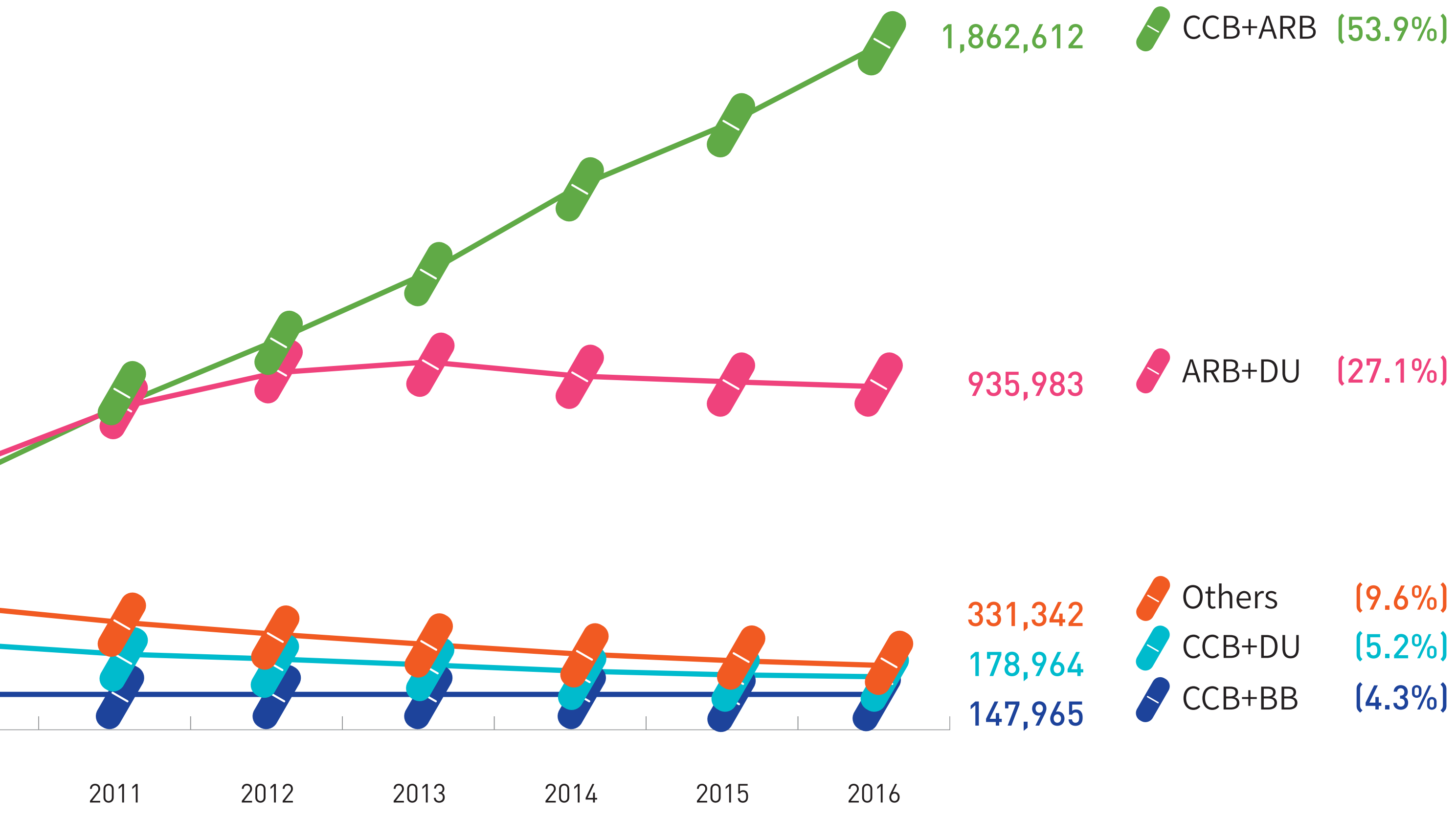

Data source: NHIBD (2002-2016)

Changes in dual therapy composition

(among people with persistent treatment)

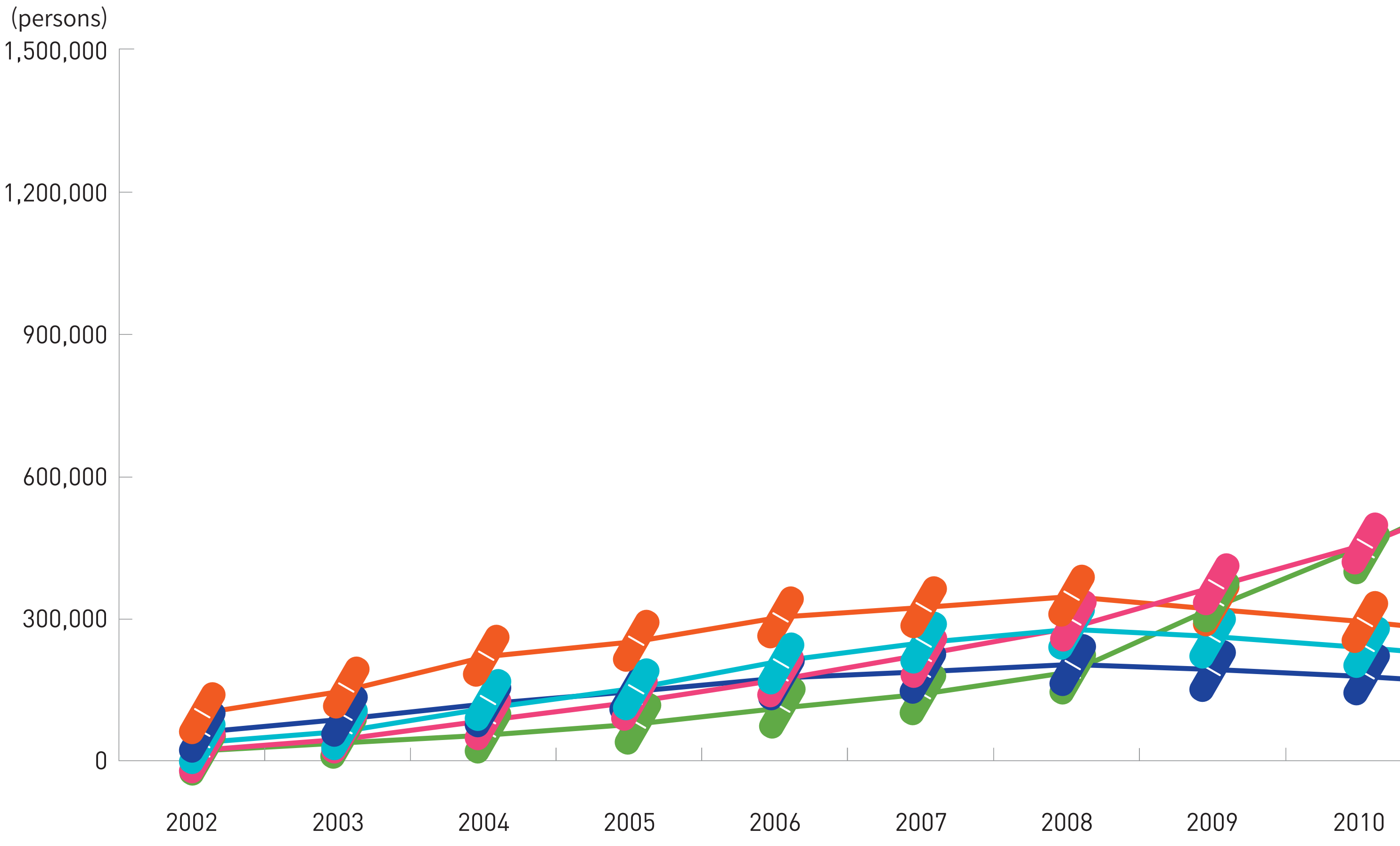

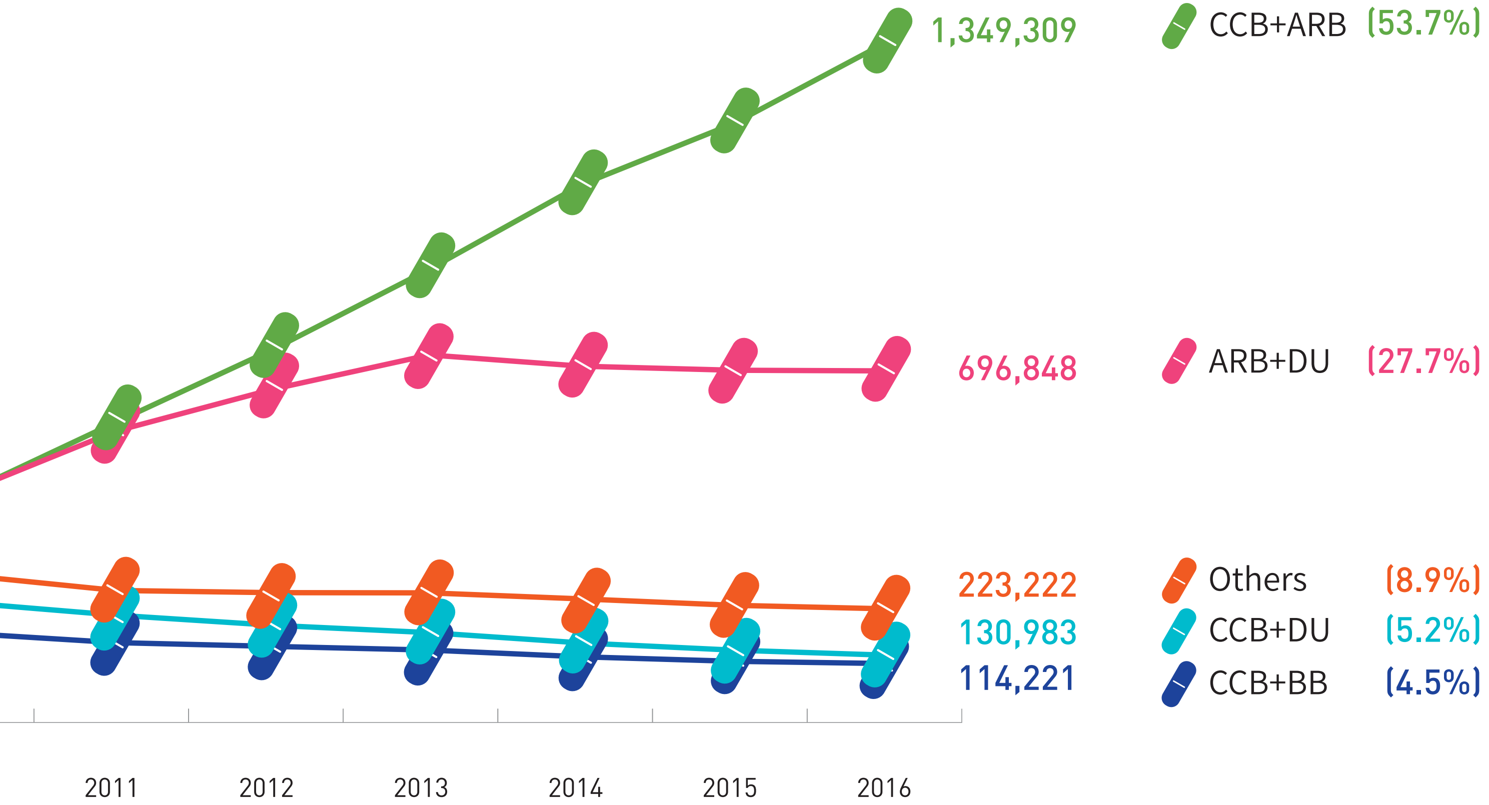

Data source: NHIBD (2002–2016)

Please cite this fact sheet as: The Korean Society Hypertension (KSH) – Hypertension Epidemiology Research Working Group.
Korea Hypertension Fact Sheet 2018. Clinical Hypertension 2018;24:** https://doi.org/10.1186/*******
