# Supplementary material for: Korea hypertension fact sheet 2018
Source: Clin Hypertens. 2018 Oct 1;24:13. doi: 10.1186/s40885-018-0098-0 (PMC6166277; doi:10.1186/s40885-018-0098-0)
Supplement: Supplementary file 2 — Korea hypertension fact sheet 2018 - Extended Korean version. (PDF 1208 kb) [file 40885_2018_98_MOESM2_ESM.pdf]

KOREA HYPERTENSION FACT SHEET 2018

대한고혈압학회

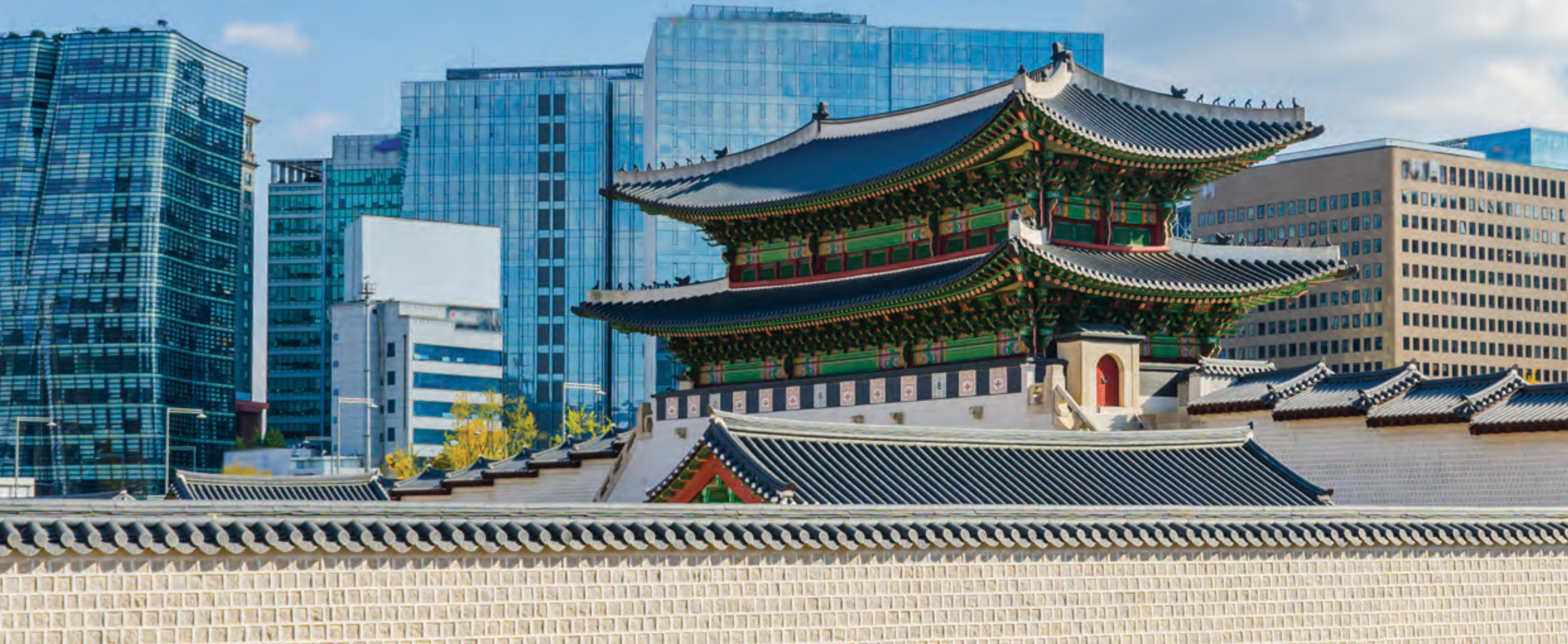

KOREA HYPERTENSION FACT SHEET 2018

발행인 조명찬
편집장 김현창
편집 대한고혈압학회 고혈압역학연구회
디자인 인권앤파트너스

대한고혈압학회

**회장**	정진원	원광의대
**차기회장**	신길자	이화의대
**부회장**	김동수	인제의대
	김근호	한양의대
**이사장**	조명찬	충북의대
**총무이사**	강석민	연세의대
**기획이사**	편욱범	이화의대
**학술이사**	이해영	서울의대
**간행이사**	정욱진	가천의대
**재무이사**	임상현	가톨릭의대
**연구이사**	김광일	서울의대
**교육이사**	조은주	가톨릭의대
**홍보이사**	손일석	경희의대
**국제교류이사**	박성하	연세의대
**보험이사**	신진호	한양의대
**정책이사**	성기철	성균관의대
**의료정보이사**	유승기	을지의대
**윤리이사**	성지동	성균관의대

**이사**	김대중	아주의대
	김현창	연세의대
	류동열	이화의대
	양동헌	경북의대
	유병수	연세원주의대
	이무용	동국의대
	이승훈	서울의대
	이은미	원광의대
	정중화	조선의대
	정진옥	충남의대
	한진	인제의대
	홍영미	이화의대
	황진용	경상의대
**감사**	박창규	고려의대
	임세중	연세의대

고혈압역학연구회

**회장**		김현창	연세의대
**부회장**		안성복	이화여대
**상임위원**	**연구**	지선하	연세보건대학원
	**학술**	박성하	연세의대
	**기획**	이해영	서울의대
	**교육**	신민호	전남의대
	**홍보**	임상현	가톨릭의대
**고문**		박종구	연세원주의대
		서일	연세의대
		이태용	충남의대
**간사**		이승원	연세의대

발간사

고혈압은 우리나라 성인의 4분의 1정도가 가진 '국민병'이 되었습니다. 또한 고혈압은 전세계 사망원인 1위인 심뇌혈관질환의 가장 주요한 위험요인입니다. 고혈압은 예방이 가능할 뿐만 아니라 발생하더라도 조기에 진단하고 체계적으로 관리하면 치명적인 합병증을 예방할 수 있는 질환입니다. 그 동안 대한고혈압학회의 노력과 정부의 고혈압 예방관리 사업 및 보건의료인들의 헌신에 힘입어 고혈압에 대한 국민들의 인식 수준이 높아지고 고혈압 관리수준도 크게 향상되었습니다.

대한고혈압학회는 1994년 창립한 이후 지속적인 학술연구와 고혈압 예방 및 관리사업으로 우리나라의 고혈압 인지율, 치료율, 조절률을 세계적 수준으로 끌어올리는데 크게 기여하였다고 자부합니다. 대한고혈압학회는 ‘고혈압 관리를 통한 국민 건강수준 향상’이라는 새로운 비전을 정립하였으며, 고혈압의 진단 · 치료 · 예방과 관리를 위한 과학적 근거를 확립, 대국민 홍보를 통한 고혈압 인지도를 향상, 고혈압 관련 정책수립의 주도적 역할, 고혈압 분야 의 진정한 글로벌 리더라는 미션을 수립하였습니다. 책임감, 참여, 리더십, 전문성이라는 핵심가치 아래 구체적이고 실행가능한 전략들을 세워 정부, 유관기관, 언론, 관련학회와 함께 추진하고 있습니다.

이에 대한고혈압학회는 우리나라 고혈압 현황에 대한 정확하고 지속적인 모니터링이 시급하다고 판단하여 전국민을 대표할 만한 주요 자료원을 활용하여 고혈압 팩트시트를 발간하게 되었습니다. 이는 우리나라의 고혈압 유병 및 관리 현황을 객관적 데이터로 보여주는 자료로, 정부기관과 보건의료 전문가들이 고혈압 예방 및 관리대책을 수립하고 효과를 평가하는 기초 자료로써 활용될 수 있을 것입니다. 또한 고혈압 역학연구와 임상연구의 기초자료로 활용되어 우리나라 국민건강증진에 보탬이 될 수 있기를 바랍니다. 고혈압 팩트시트 작성을 위하여 방대한 데이터를 분석하고 해석한 고혈압역학연구회의 노력을 치하하며, 팩트시트의 근간이 되는 소중한 자료를 제공해주신 질병관리본부 건강영양조사과와 국민건강보험공단 빅데이터운영실에 깊이 감사 드립니다.

대한고혈압학회
회장 **정진원**

대한고혈압학회
이사장 **조명찬**

자료원 및 분석방법 - 국민건강영양조사

**대상자**	**분석대상**	30세 이상
	**분석연도**	1998년부터 2016년까지
**혈압 분류**	**고혈압**	① 수축기혈압이 140mmHg 이상이거나, ② 이완기혈압이 90mmHg 이상이거나, ③ 고혈압약물을 복용하는 경우
	**고혈압전단계**	① 고혈압 기준에 해당하지 않으면서, ② 수축기혈압이 120-139mmHg이거나, ③ 이완기혈압이 80-89mmHg인 경우
	**정상혈압**	위 기준에 해당하지 않는 경우
**지표 정의**	**인지율**	고혈압 유병자 중 의사로부터 고혈압 진단을 받은 사람의 분율
	**치료율**	고혈압 유병자 중 고혈압치료제를 한 달에 20일 이상 복용한 사람의 분율
	**조절률(유병자 중)**	고혈압 유병자 중 수축기혈압 <140mmHg이면서, 이완기혈압 <90mmHg인 사람의 분율
	**조절률(치료자 중)**	고혈압 치료자 중 수축기혈압 <140mmHg이면서, 이완기혈압 <90mmHg인 사람의 분율

자료원 및 분석방법 - 국민건강보험 빅데이터

**대상자**	**분석대상**	전 연령
	**분석연도**	2002년부터 2016년까지
**지표 정의**	**고혈압 의료 이용**	매 해 고혈압을 진단명으로 1회 이상 진료한 경우
	**고혈압 치료**	매 해 고혈압 치료제를 1회 이상 처방한 경우
	**고혈압 지속치료**	매 해 고혈압 치료제를 290일(80%) 이상 처방한 경우
**고혈압 치료제**	**치료제 분류**	이뇨제(Diuretics, DU), 베타차단제(Beta-blocker, BB)<br>칼슘채널차단제(Calcium Channel Blocker, CCB),<br>안지오텐신차단제(angiotensin receptor blocker, ARB), ACE 길항제(ACE inhibitor, ACE)<br>기타(알도스테론 길항제, 알파차단제, 혈관확장제 등)
	**대표 처방**	고혈압 치료제 처방이 변하는 경우, 매 해 가장 오래 동안 사용한 처방을 해당 환자의 대표 처방으로 정의하고 이에 따라 고혈압 치료제 사용을 분류

Contents

	06	Summary
	07	고혈압 인구 현황 요약
	08	고혈압 치료 현황 요약
1 평균 혈압 및 고혈압 규모의 변화	11	평균 혈압 변화 (30세이상, 연령표준화)
	12	고혈압 유병률 변화 (30세 이상, 연령표준화)
	13	성·연령별 고혈압 유병률 변화
	14	고혈압 유병인구 변화 (추정)
2 고혈압 관리지표의 변화	16	성별 유병률 및 관리지표 (30세 이상)
	17	고혈압 관리지표 변화 (30세 이상, 연령표준화)
	18	성별 고혈압 인지율 변화 (30세 이상, 연령표준화)
	19	성별 고혈압 치료율 변화 (30세 이상, 연령표준화)
	20	성별 고혈압 조절률(유병자 중) 변화 (30세 이상, 연령표준화)
	21	성별 고혈압 조절률(치료자 중) 변화 (30세 이상, 연령표준화)
	22	성·연령별 고혈압 인지율 변화
	23	성·연령별 고혈압 치료율 변화
	24	성·연령별 고혈압 조절률(유병자 중) 변화
	25	성·연령별 고혈압 조절률(치료자 중) 변화
3 고혈압 의료이용 현황	27	고혈압 의료이용, 치료, 지속치료 환자수 변화
	28	고혈압 치료 환자의 연령 분포
	30	고혈압과 이상지질혈증, 당뇨병 동반 치료자
	32	고혈압 약물 처방 변화 (전체치료자 중)
	34	고혈압 약물 처방 변화 (지속치료자 중)
	36	고혈압 약물 단독요법 구성 변화 (전체치료자 중)
	38	고혈압 약물 단독요법 구성 변화 (지속치료자 중)
	40	고혈압 2제요법 구성 변화 (전체치료자 중)
	42	고혈압 2제요법 구성 변화 (지속치료자 중)

Summary

- 우리나라 성인 인구 중 1,100만명 이상이 고혈압을 가진 것으로 추정됨.
- 30세 이상 인구의 고혈압 유병률은 29%이며, 고혈압 유병자 중 인지율은 65%, 치료율은 61%, 조절률은 44%임.
- 고혈압 인지율, 치료율, 조절률은 2007년까지 빠르게 향상되었으나, 이후 10년간 큰 변화가 없음.
- 고혈압 유병자중 30-49세의 젊은 연령층에서 인지율, 치료율, 조절률이 50% 미만으로 매우 낮음.
- 고혈압 치료를 위해 연 1회이상 의료기관을 방문하는 사람은 890만명이며, 꾸준히 고혈압 치료제를 처방받는 사람은 570만명임.
- 고혈압 치료자 중 65세 이상 고령자의 비중이 2002년 34%에서 2016년 46%로 빠르게 증가함.
- 고혈압 치료자 중 57%가 당뇨병 혹은 이상지질혈증 치료를 같이 받고 있음.
- 고혈압 치료자 중 60%가 2가지 이상의 고혈압치료제를 사용하고 있음.
- 단일요법으로 가장 많이 사용되는 고혈압치료제는 안지오텐신차단제(43%)와 칼슘채널차단제(43%)이며, 2제요법으로는 칼슘채널차단제와 안지오텐신차단제 병합요법(54%)이 가장 많이 사용됨.

고혈압 인구 현황 요약

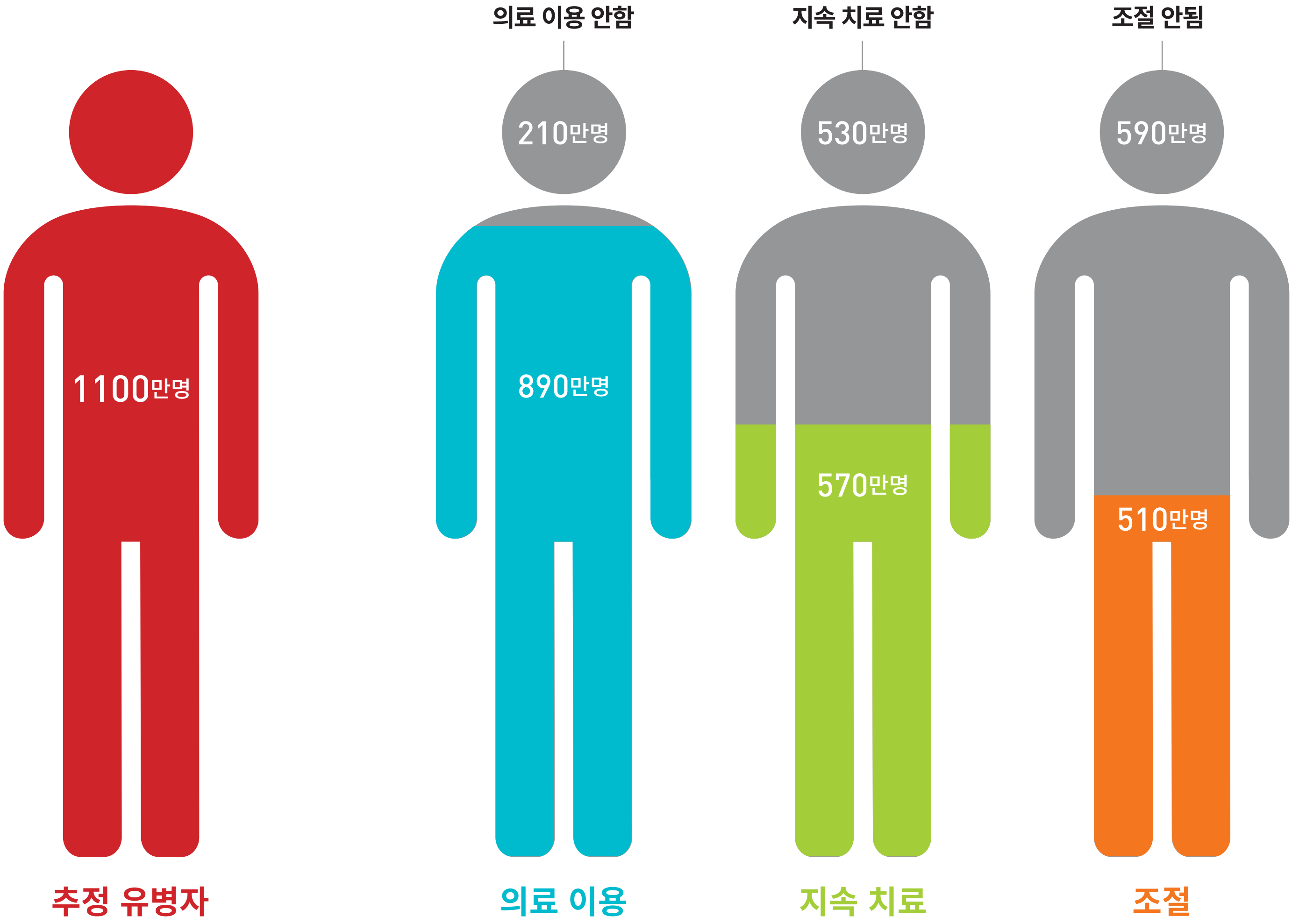

 자료원: 국민건강영양조사 (2016), 국민건강보험공단 빅데이터 (2016)

고혈압 치료 현황 요약

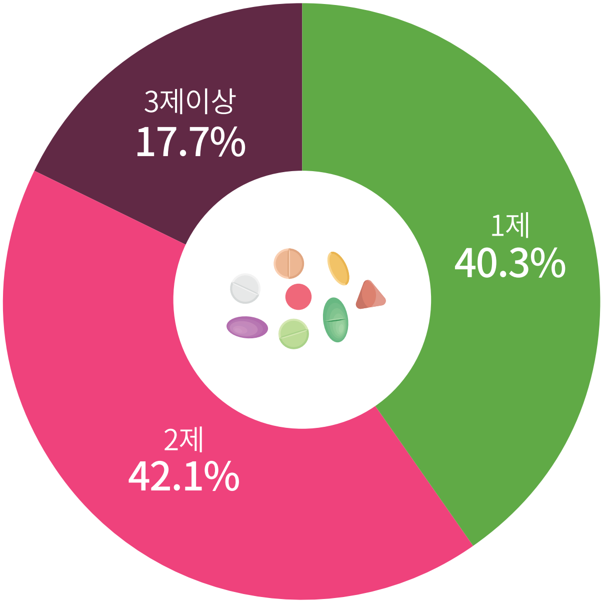

약물처방
(전체 치료자 8,219,104명 중)

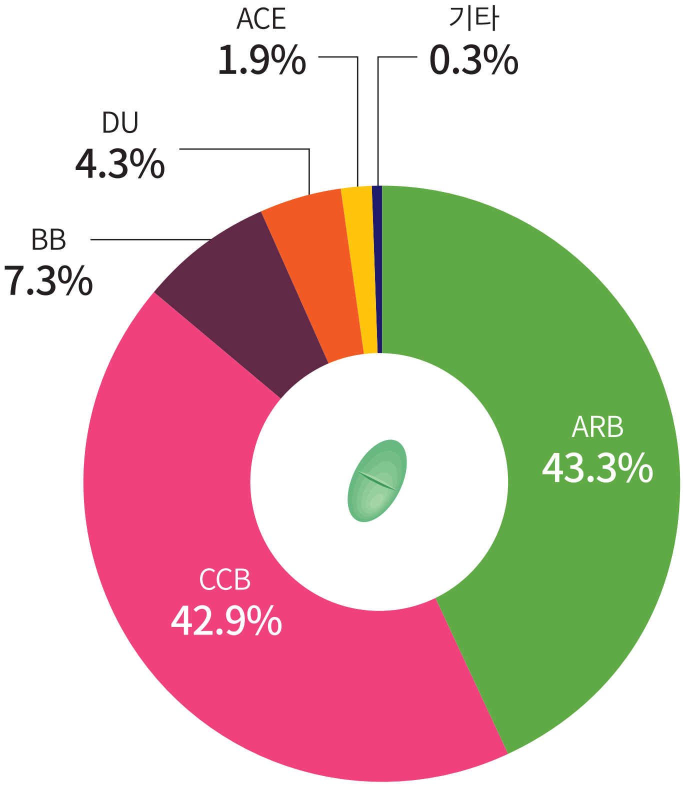

1제요법 구성
(1제요법 치료자 3,311,114명 중)

자료원: 국민건강보험 빅데이터 (2016)

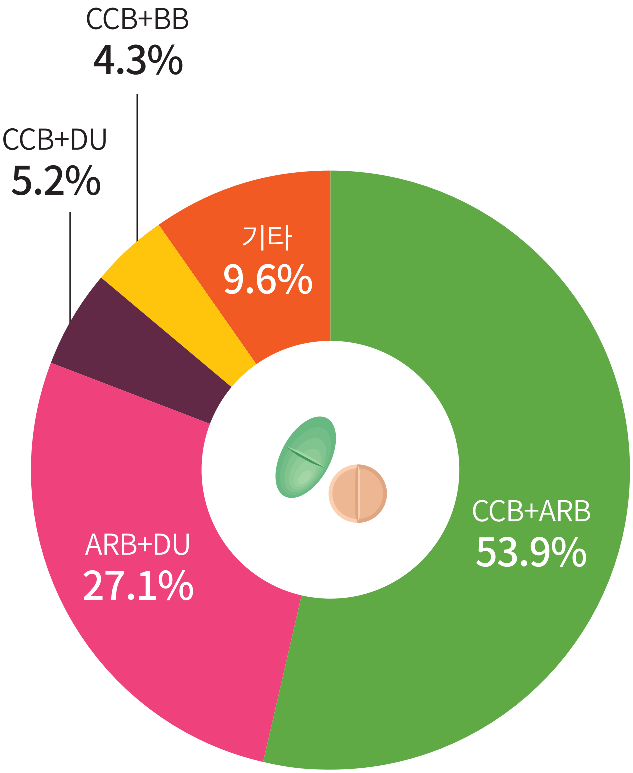

2제요법 구성

(2제요법 치료자 3,456,866명 중)

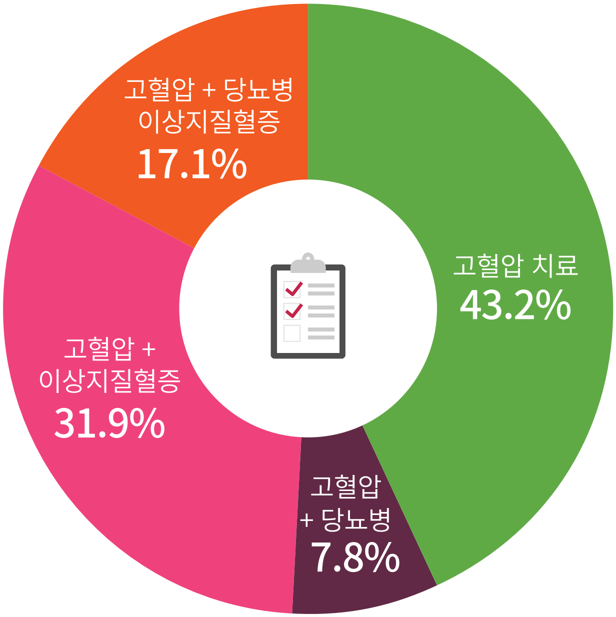

당뇨병, 이상지질혈증 동반치료

(전체 치료자 8,219,104명 중)

자료원: 국민건강보험 빅데이터 (2016)

1 평균 혈압 및 고혈압 규모의 변화

자료원: 국민건강영양조사

평균 혈압 변화

(30세이상, 연령표준화)

118/77 mmHg

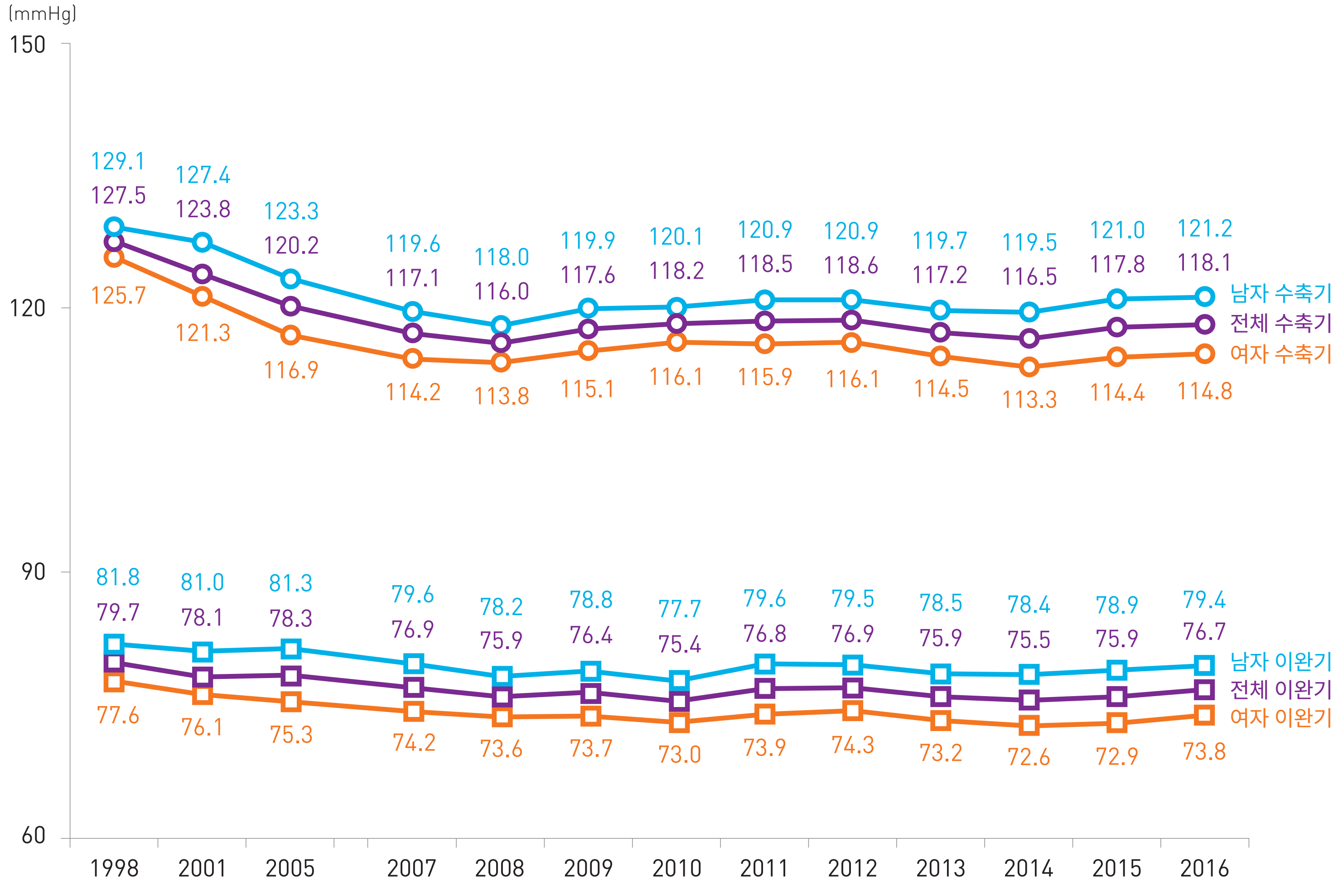

 자료원: 국민건강영양조사 (1998-2016)

고혈압 유병률 변화

(30세 이상, 연령표준화)

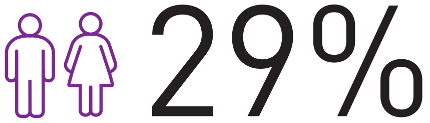

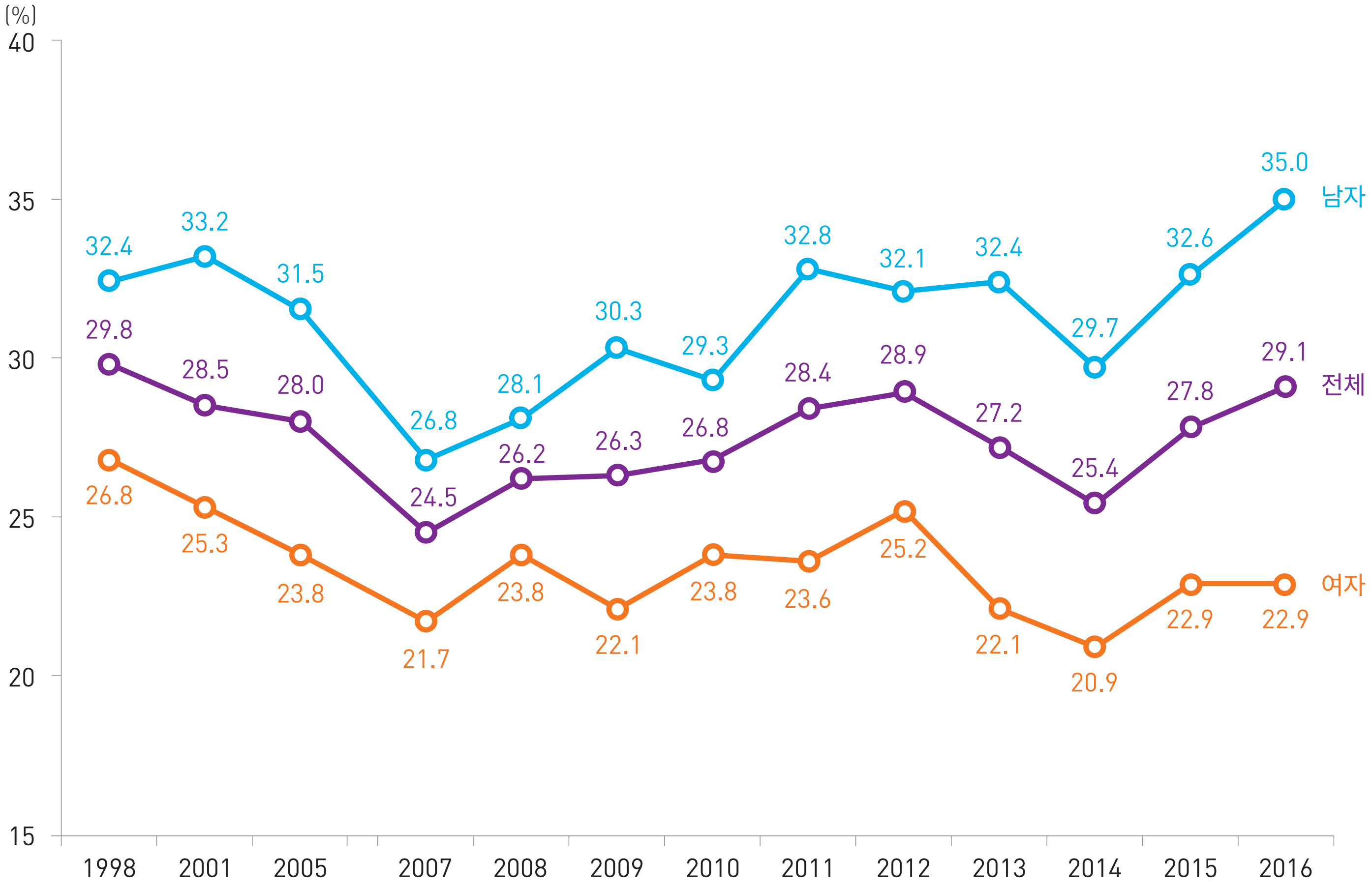

 자료원: 국민건강영양조사 (1998-2016)

성·연령별 고혈압 유병률 변화

60대까지 >
70대이후 <

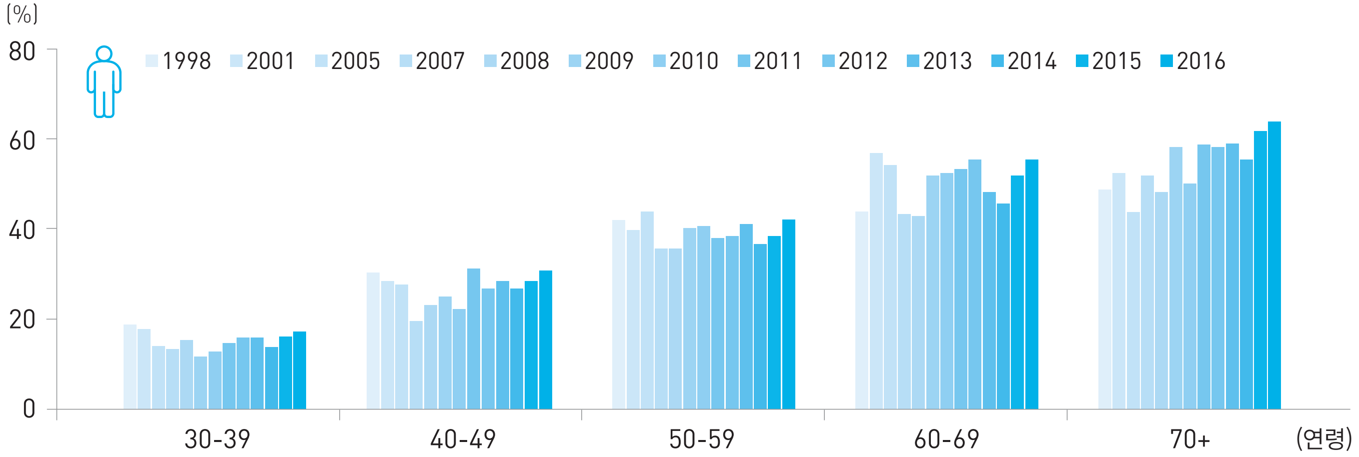

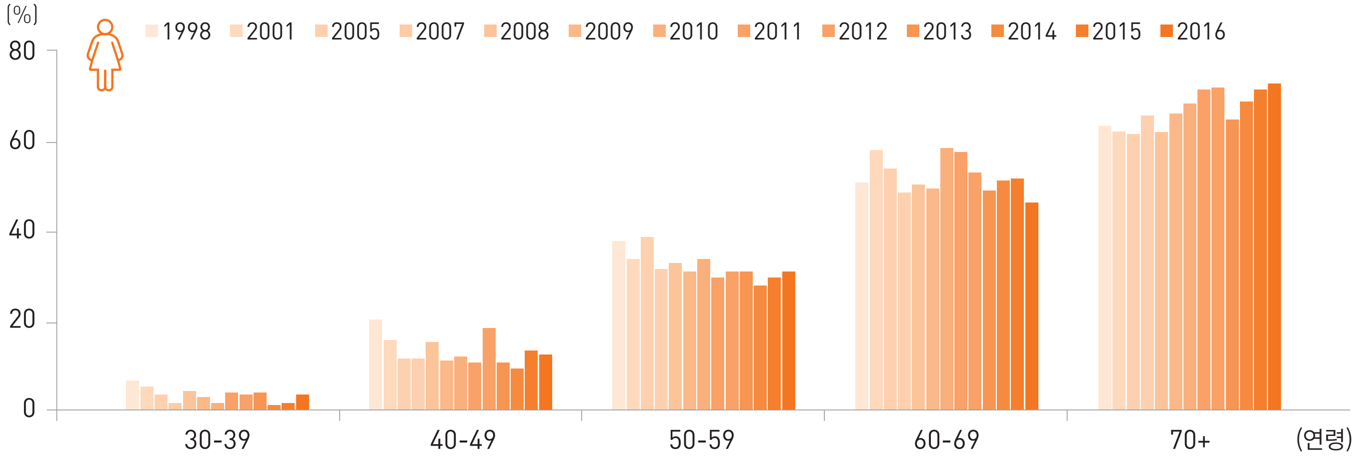

자료원: 국민건강영양조사 (1998-2016)

고혈압 유병인구 변화(추정) 1,100만명 돌파

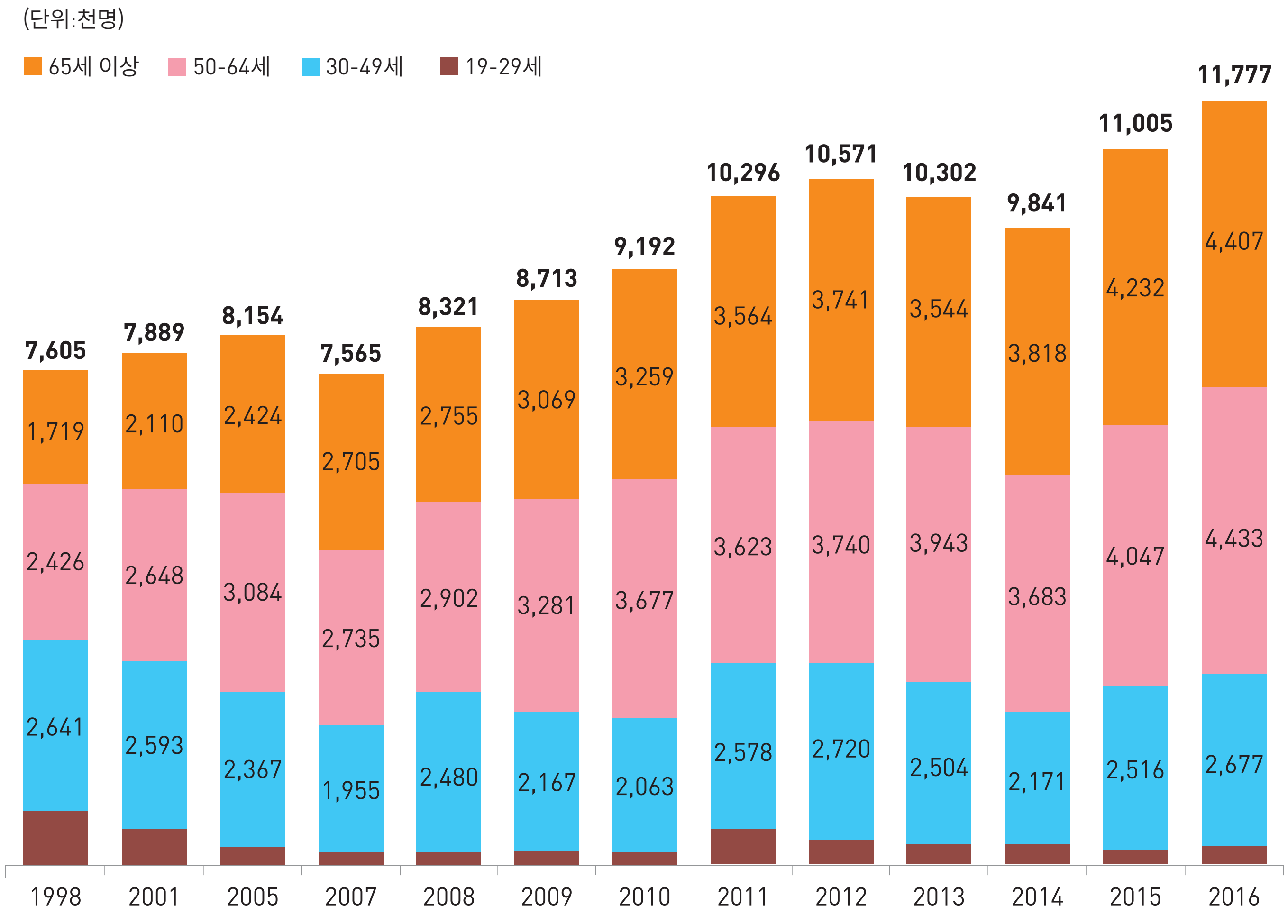

자료원: 국민건강영양조사 (1998-2016)

2 고혈압 관리지표의 변화

자료원: 국민건강영양조사

성별 유병률 및 관리지표
(30세 이상)

56%
미조절

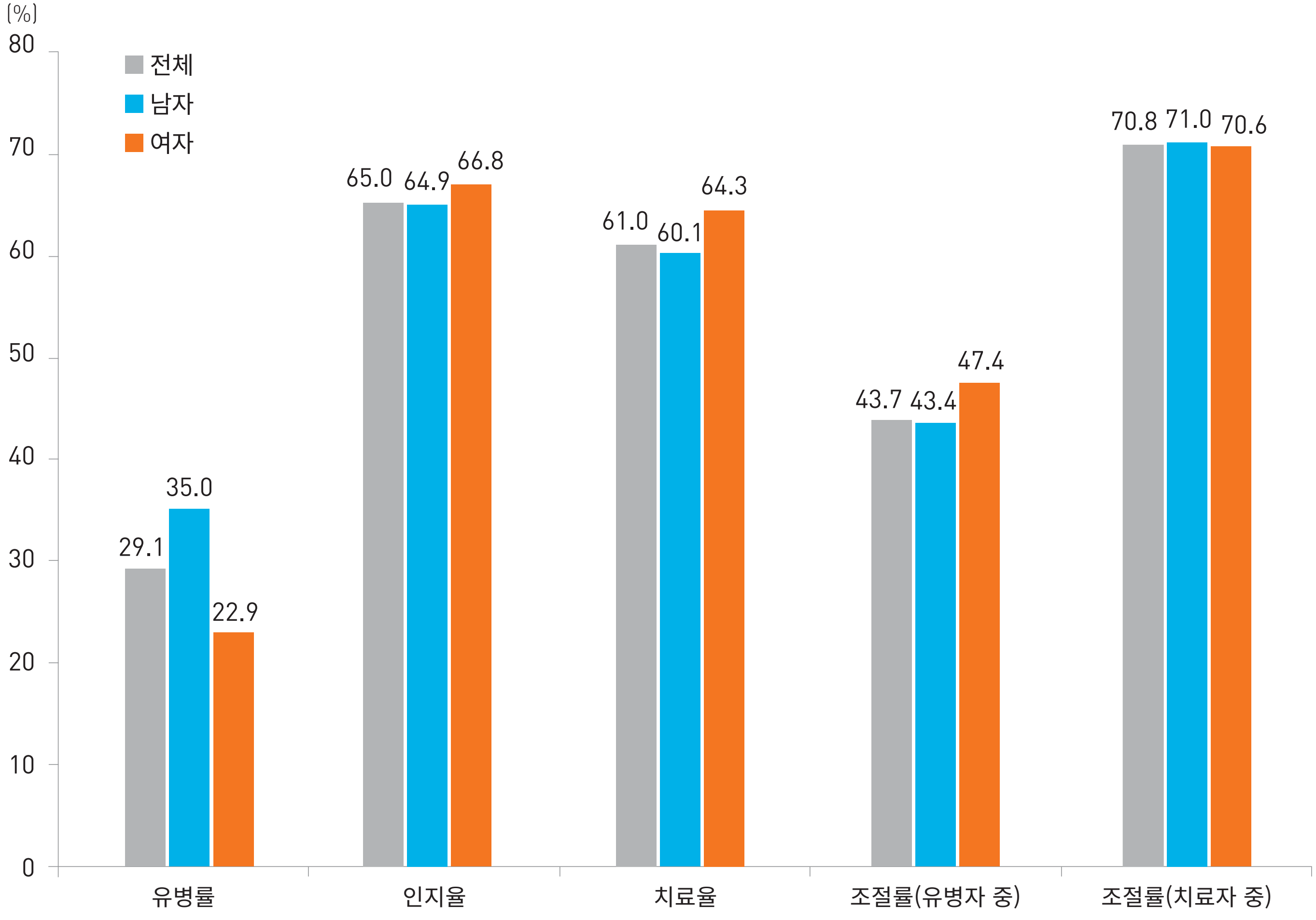

자료원: 국민건강영양조사 (2016)

고혈압 관리지표 변화

(30세 이상, 연령표준화)

'98 -'07년 빠르게 향상
최근 10년 정체

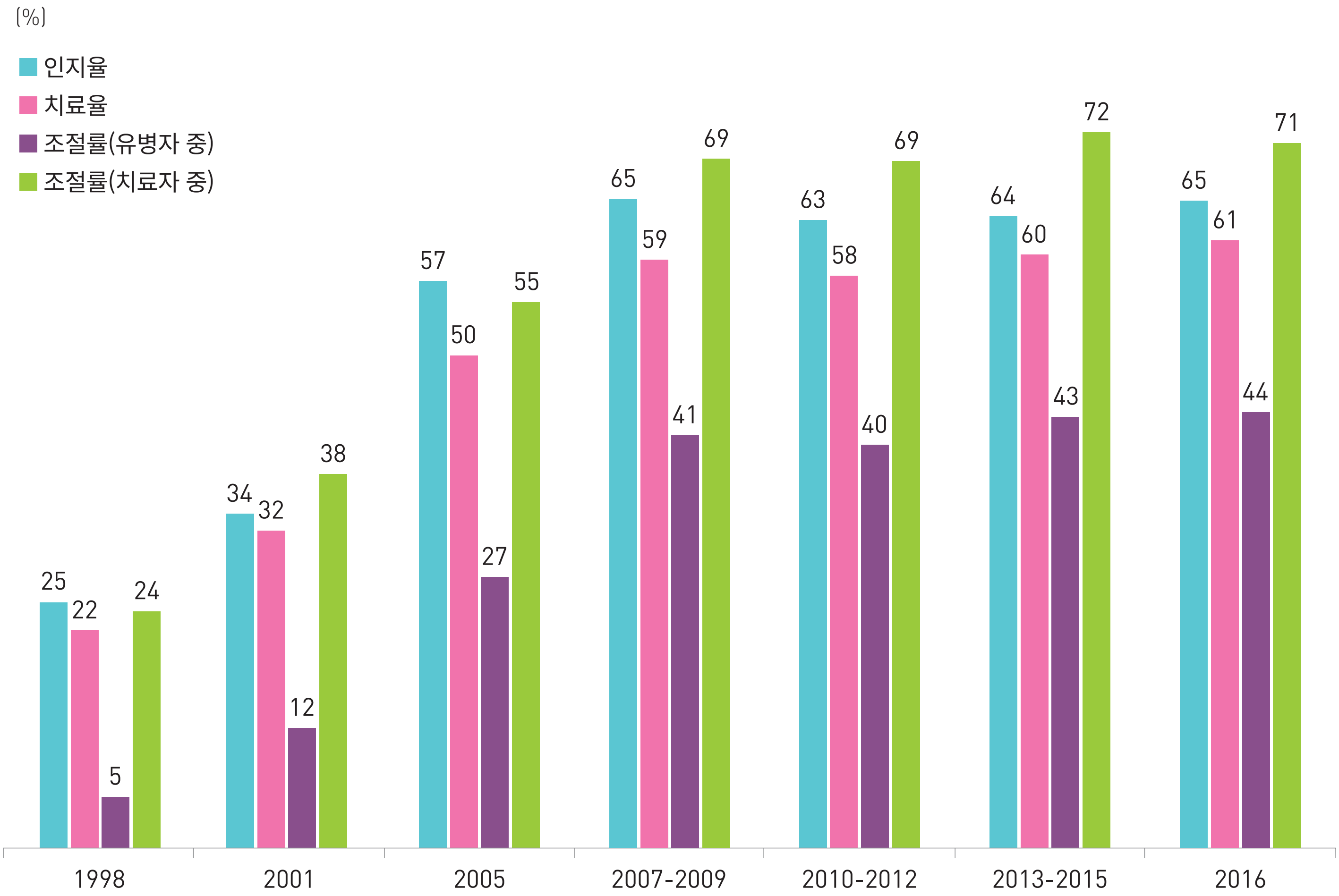

자료원: 국민건강영양조사 (1998-2016)

성별 고혈압 인지율 변화

(30세 이상, 연령표준화)

65% 67%

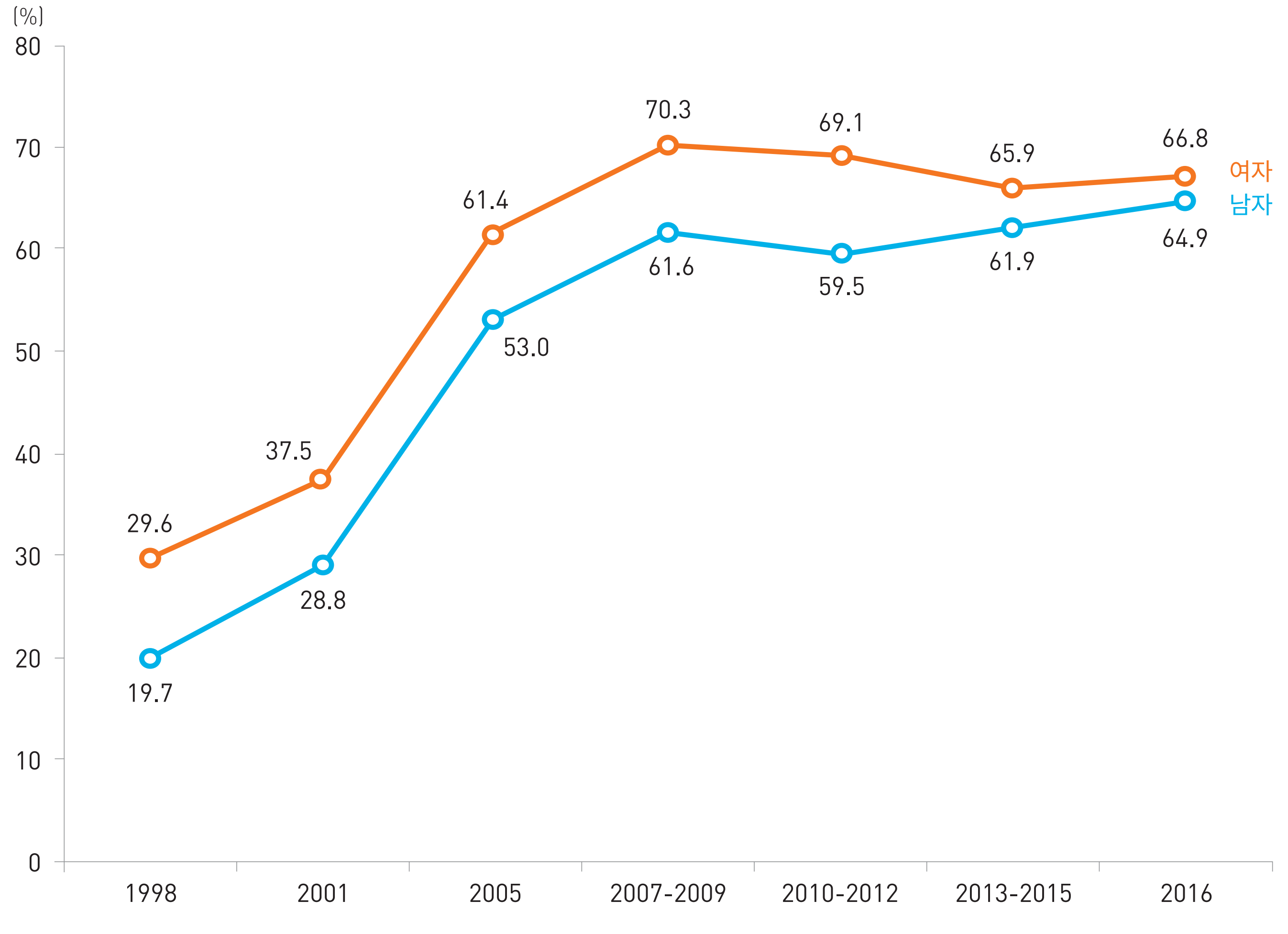

 자료원: 국민건강영양조사 (1998-2016)

성별 고혈압 치료율 변화

(30세 이상, 연령표준화)

남자 60% 여자 64%

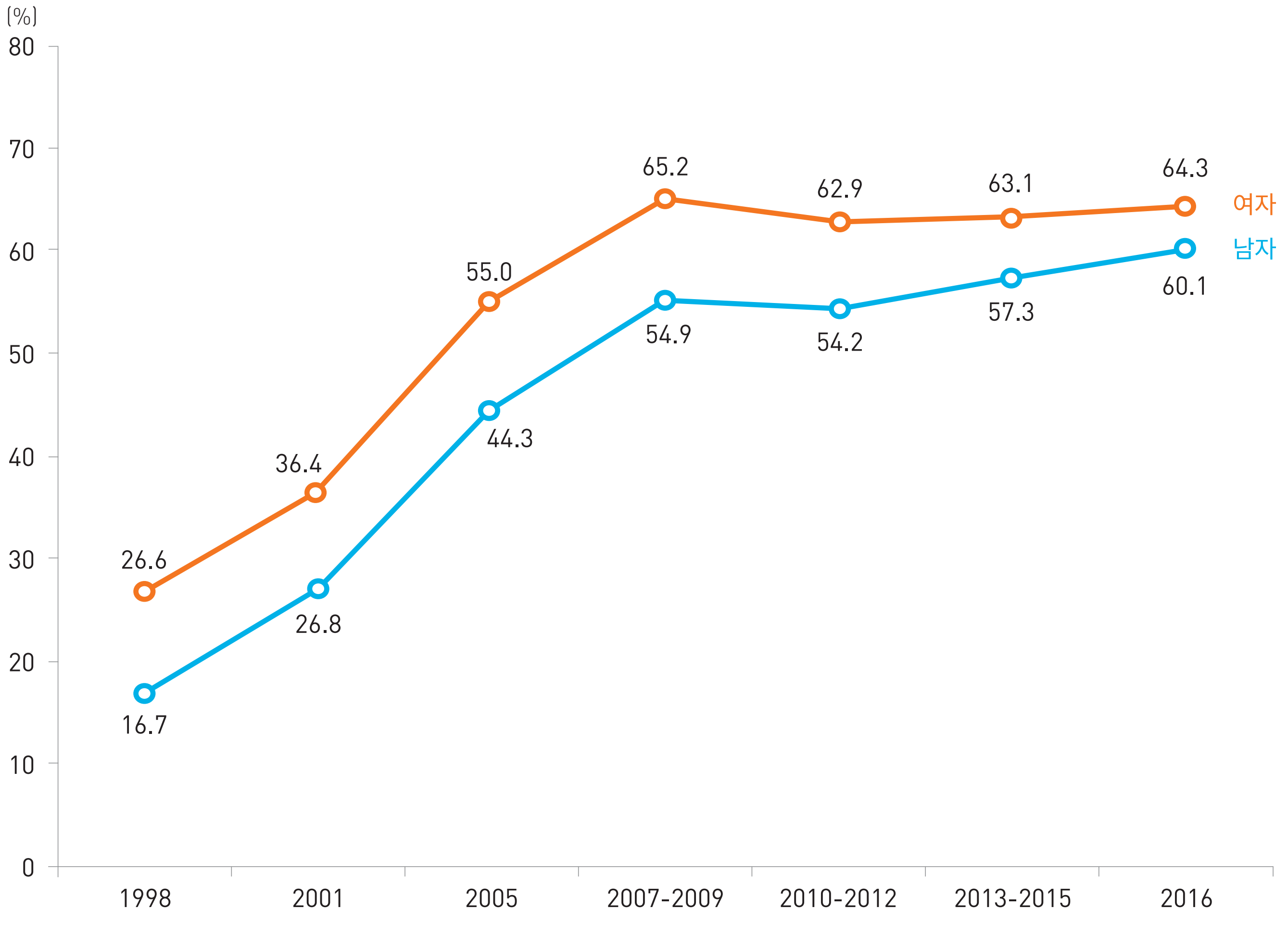

자료원: 국민건강영양조사 (1998-2016)

성별 고혈압 조절률(유병자 중) 변화

(30세 이상, 연령표준화)

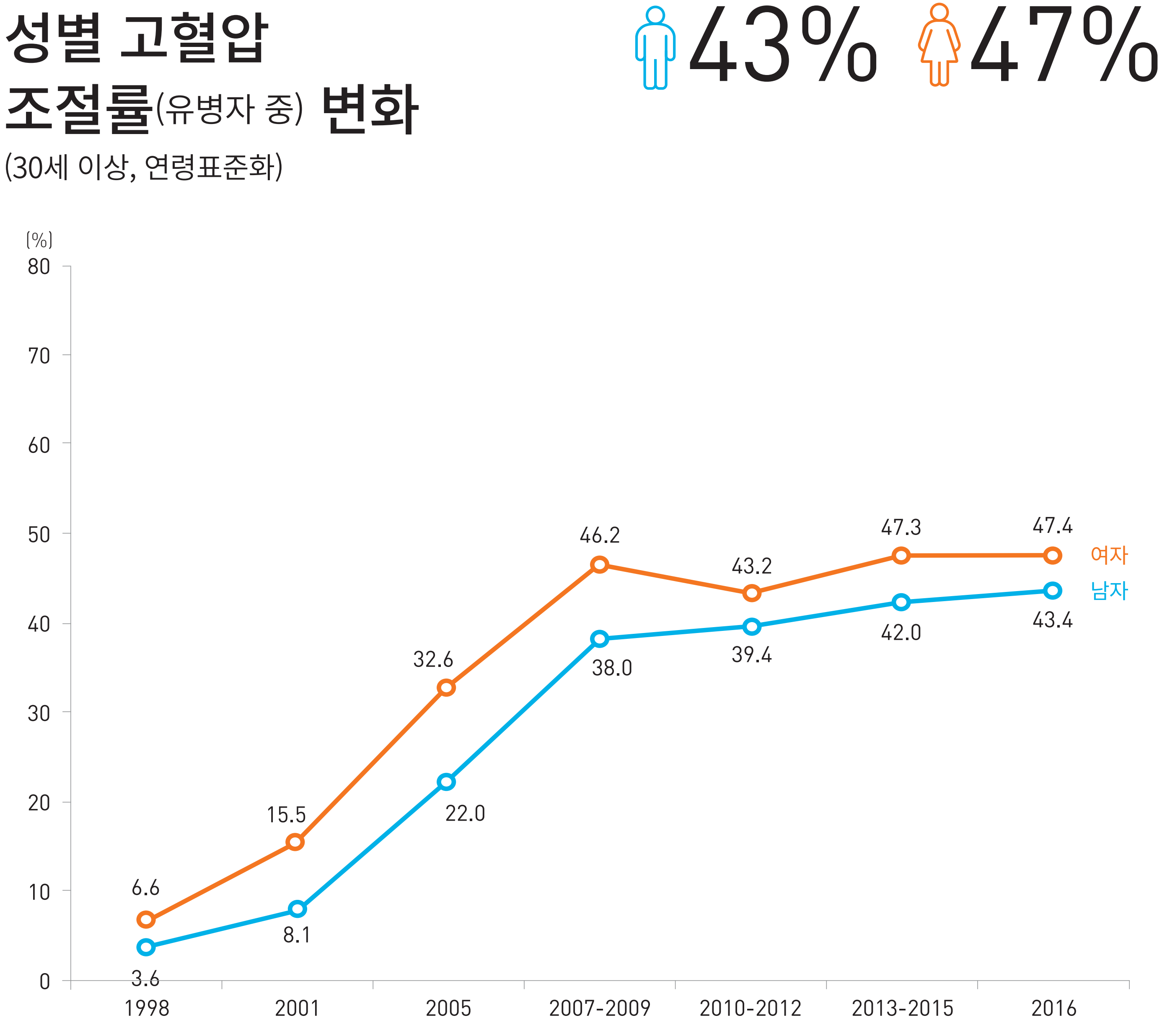

자료원: 국민건강영양조사 (1998-2016)

성별 고혈압 조절률(치료자 중) 변화

(30세 이상, 연령표준화)

남자 71% 여자 71%

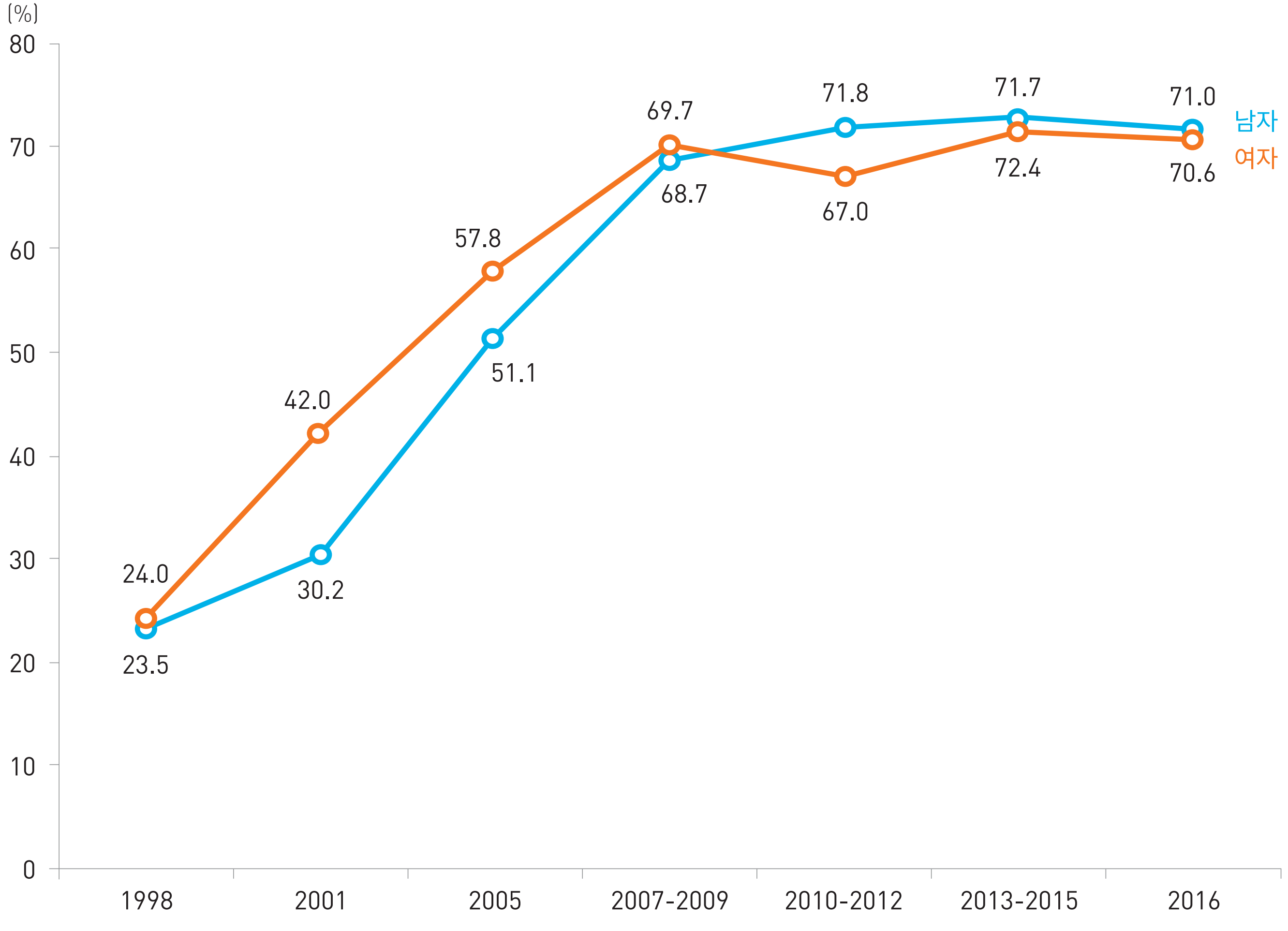

자료원: 국민건강영양조사 (1998-2016)

성·연령별 고혈압 인지율 변화

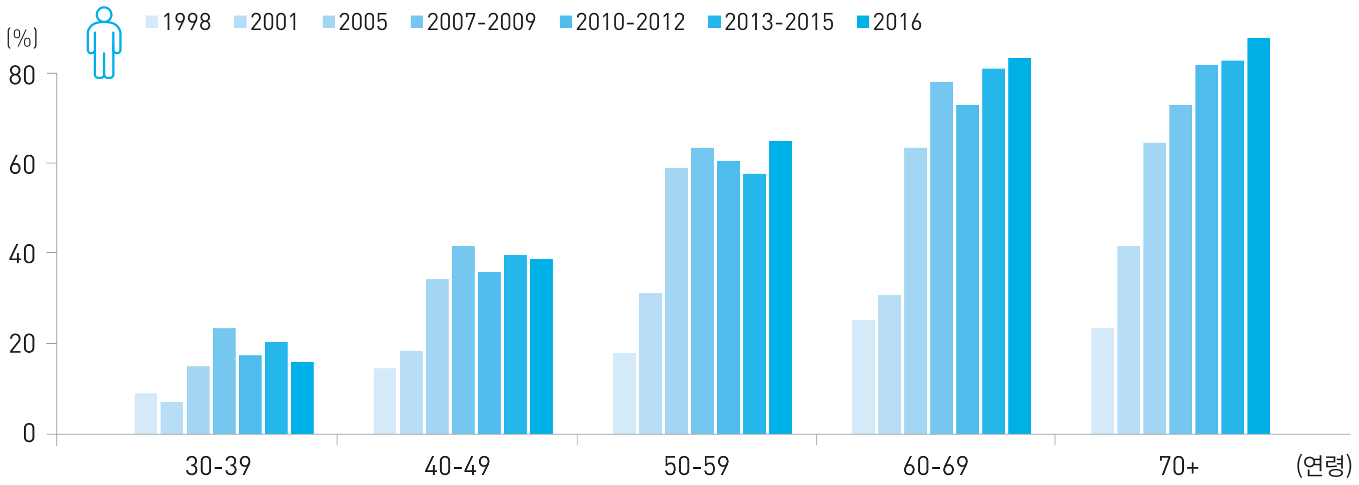

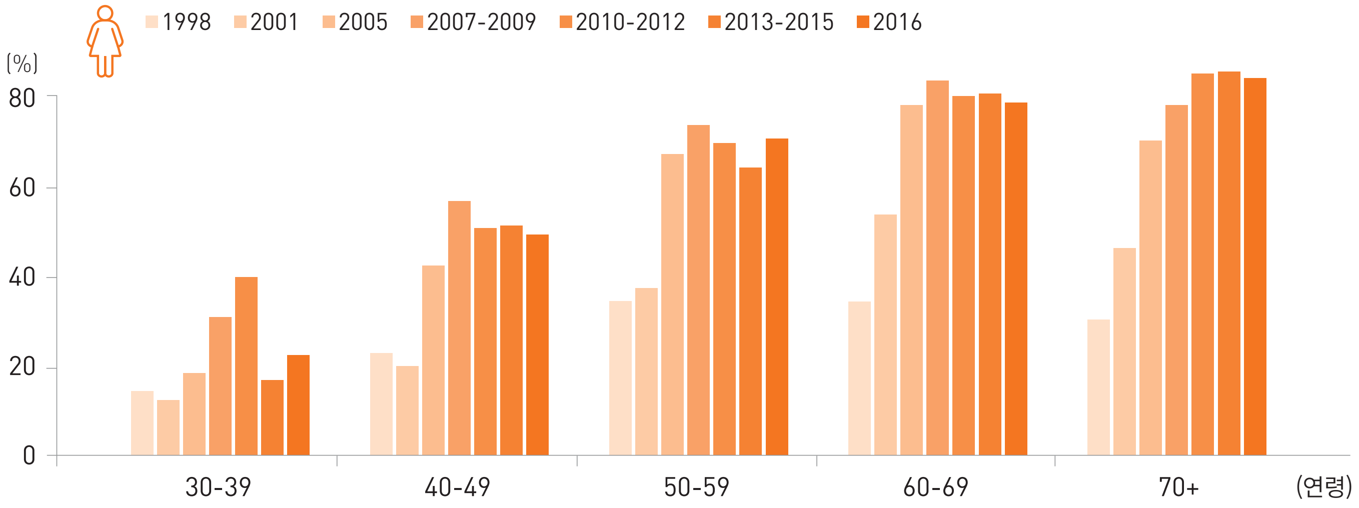

자료원: 국민건강영양조사 (1998-2016)

성·연령별 고혈압 치료율 변화

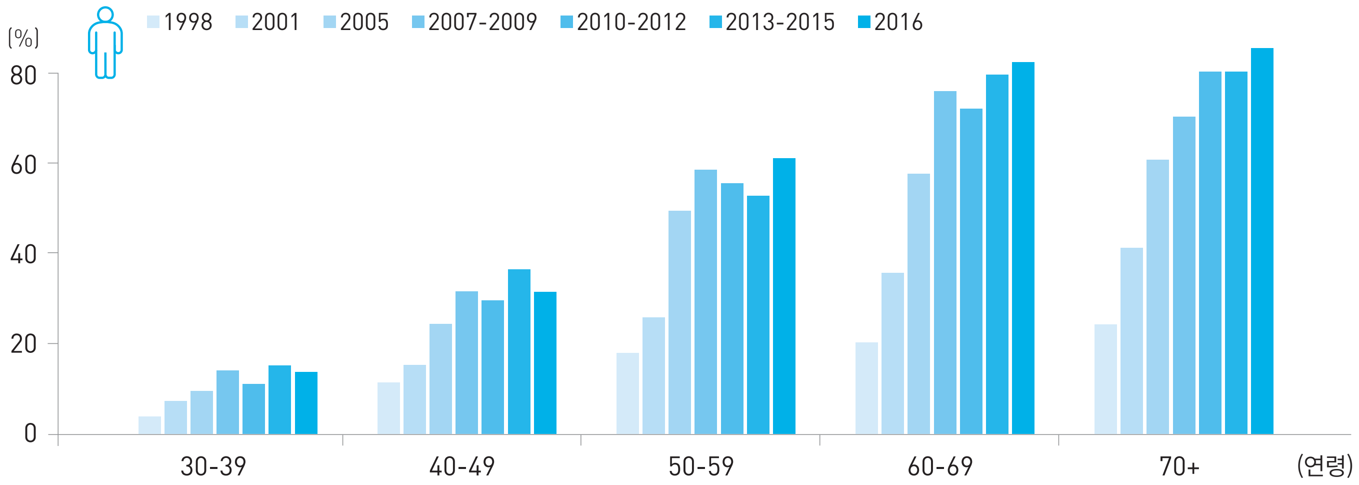

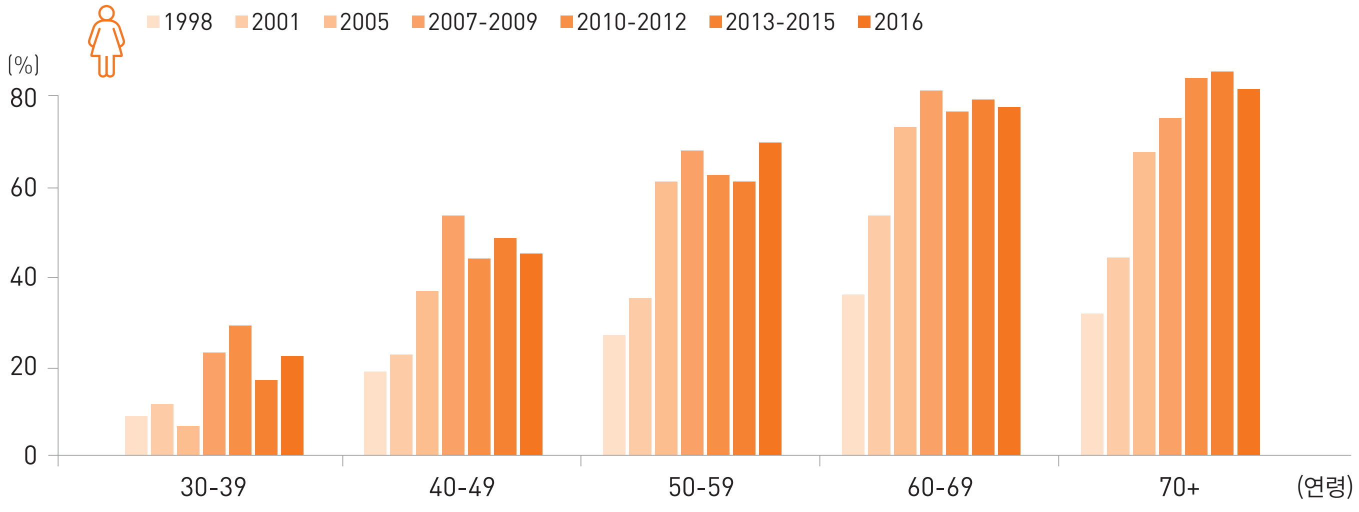

자료원: 국민건강영양조사 (1998-2016)

성·연령별 고혈압 조절률(유병자 중) 변화

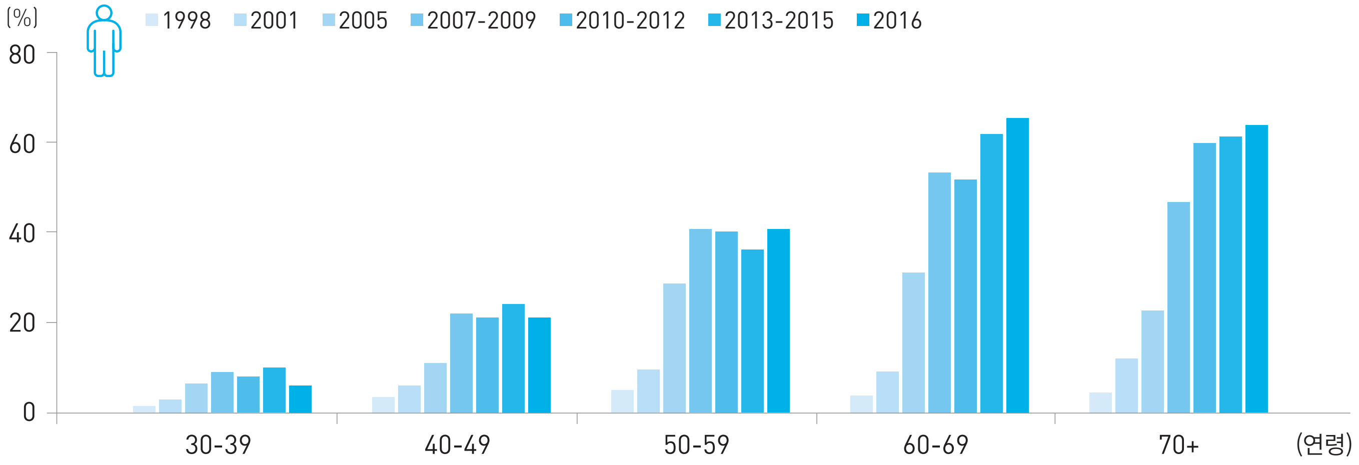

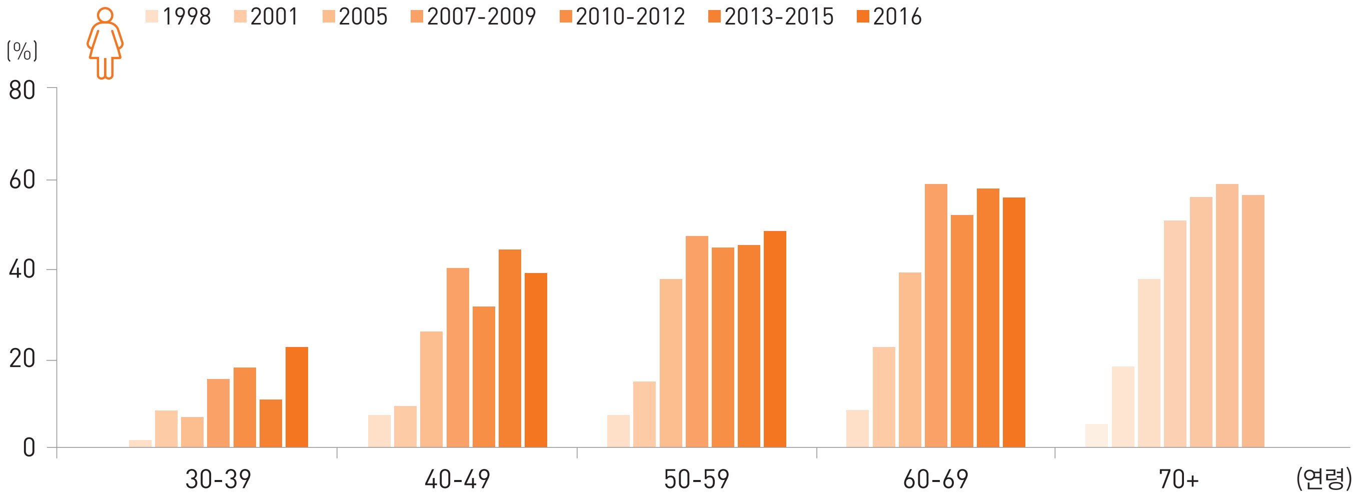

자료원: 국민건강영양조사 (1998-2016)

성·연령별 고혈압 조절률(치료자 중) 변화

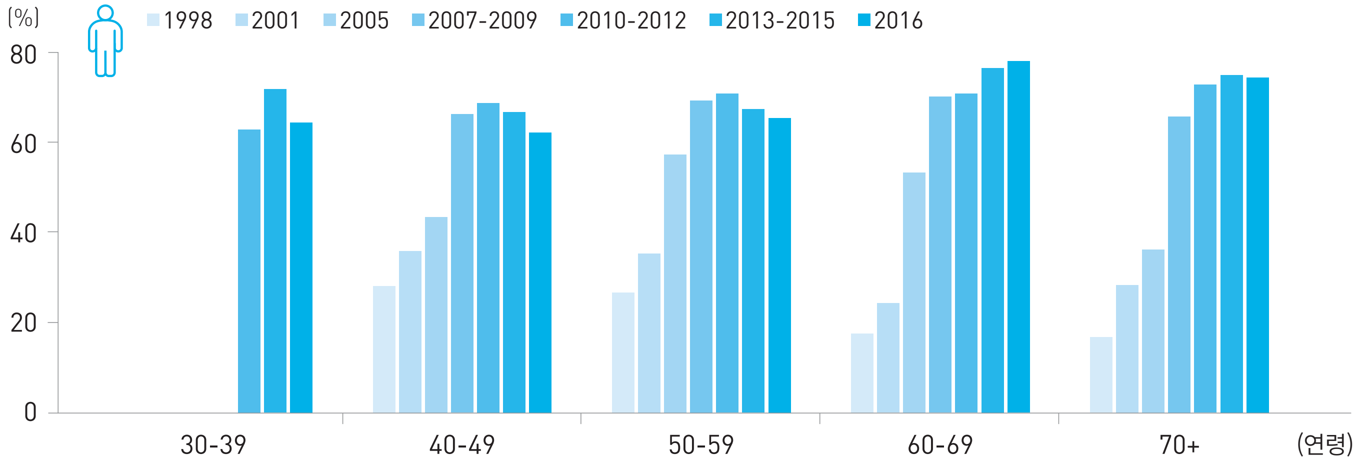

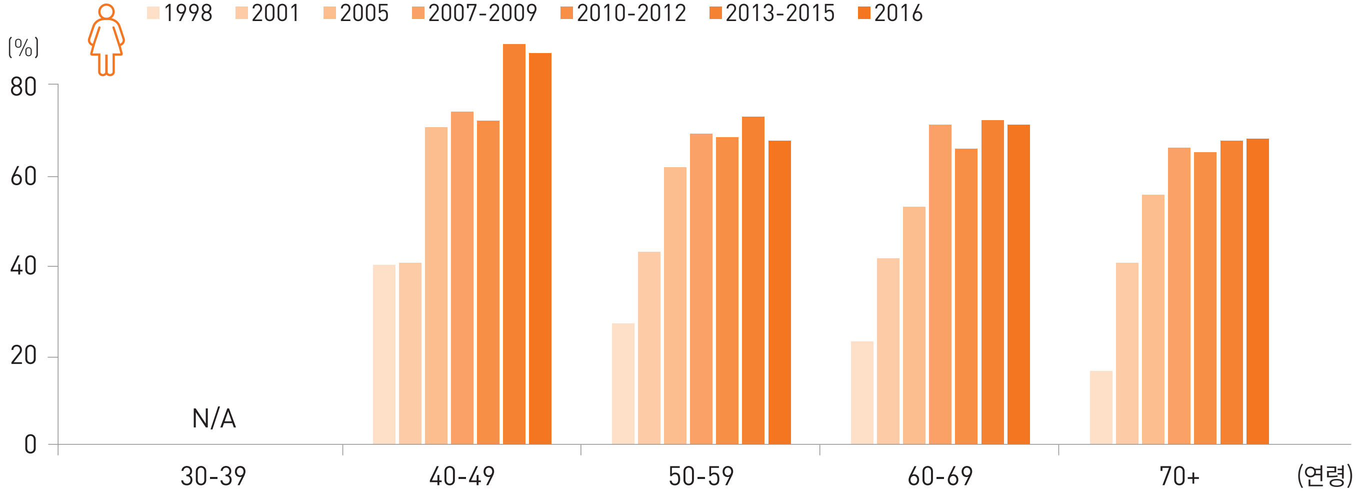

자료원: 국민건강영양조사 (1998-2016)

3 고혈압 의료이용 현황

자료원: 국민건강보험 빅데이터

고혈압 의료이용, 치료, 지속치료 환자수 변화

의료이용 8,921,829명
치료 8,219,104명
지속치료 5,734,238명

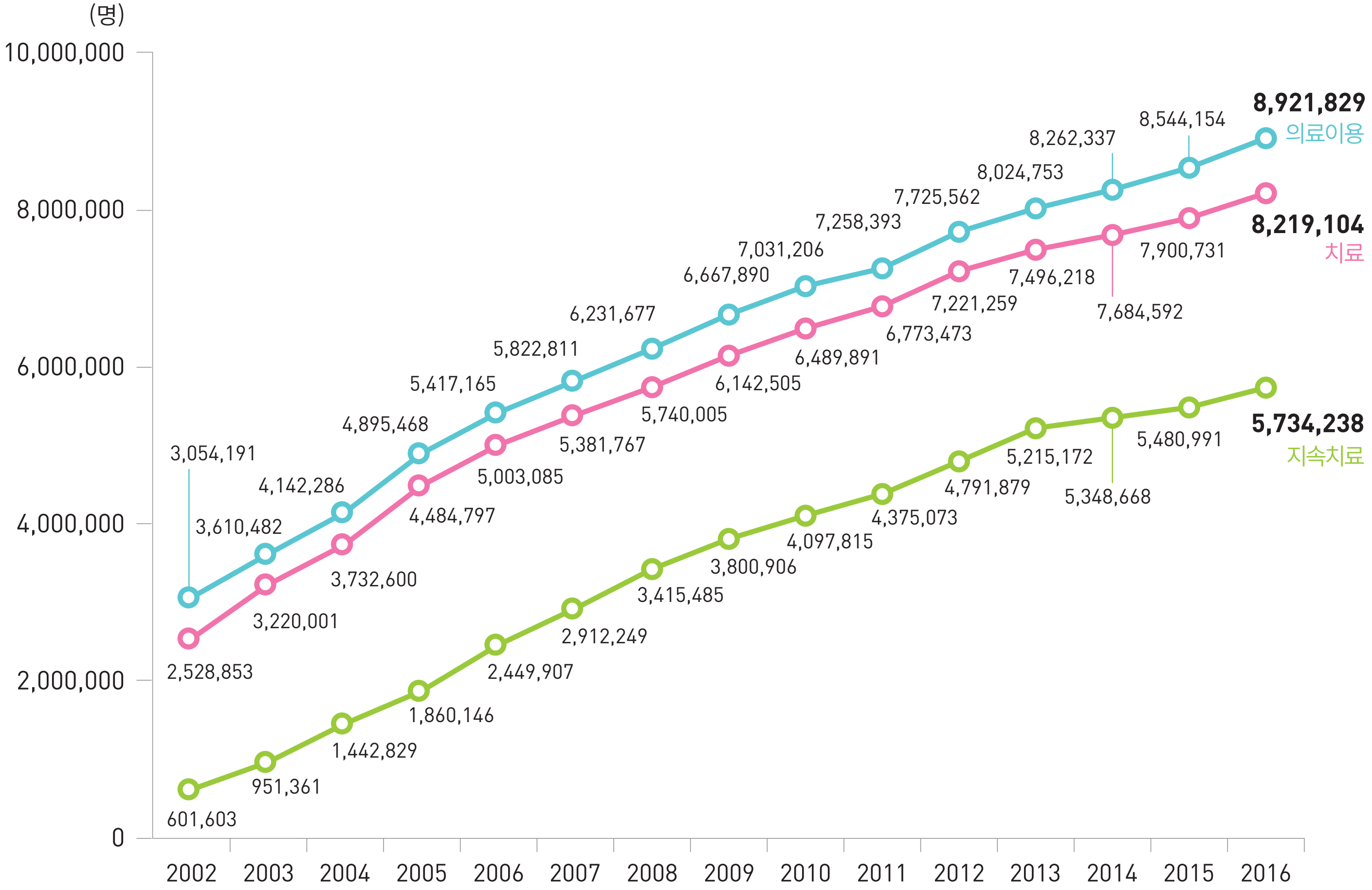

자료원: 국민건강보험공단 빅데이터 (2002-2016)

고혈압 치료 환자의 연령 분포

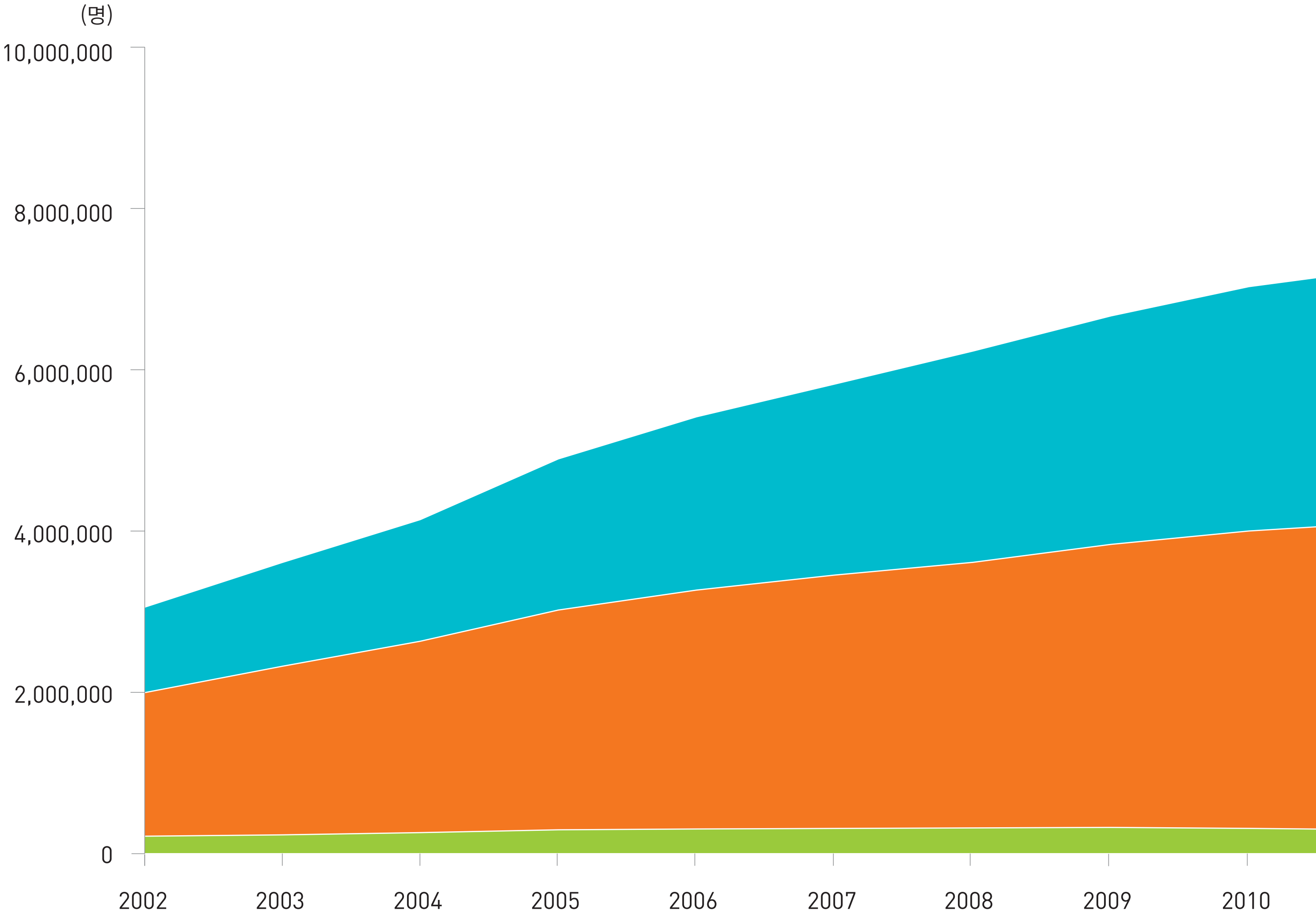

34%→46%

65세이상 고령 고혈압 환자 증가

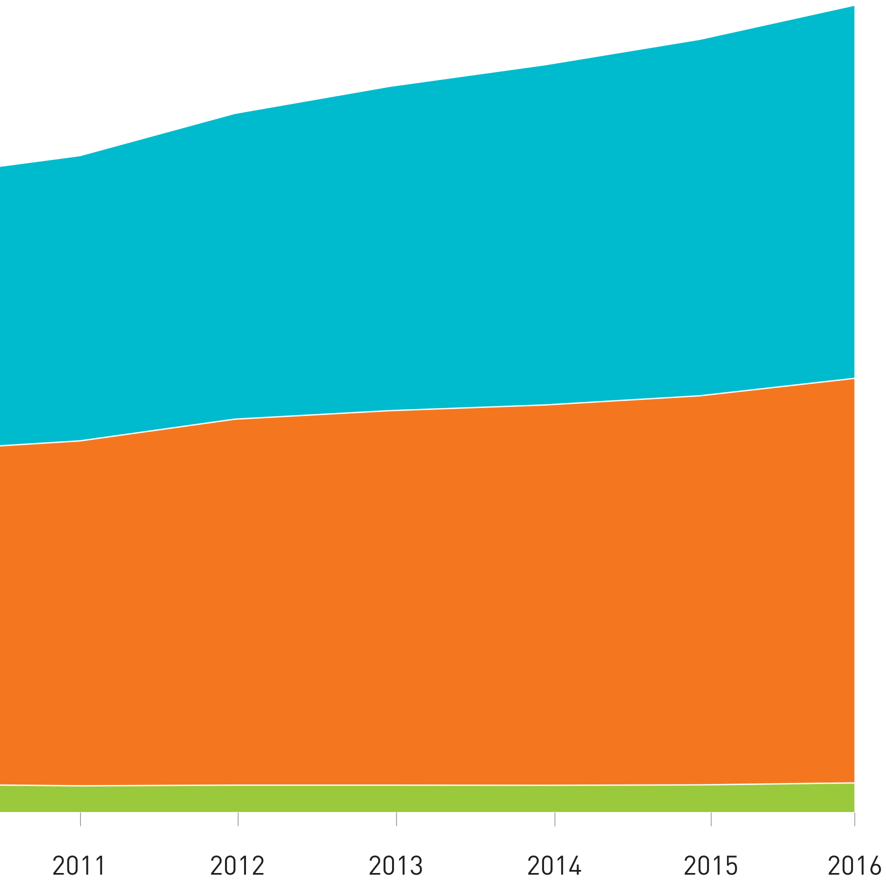

65세 이상

871,851(34.5%) → **3,777,743**(46.0%)

40-64세

1,499,322(59.3%) → **4,166,027**(50.7%)

39세 이하

157,680(6.2%) → **275,334**(3.3%)

자료원: 국민건강보험공단 빅데이터 (2002-2016)

고혈압과 이상지질혈증, 당뇨병 동반 치료자

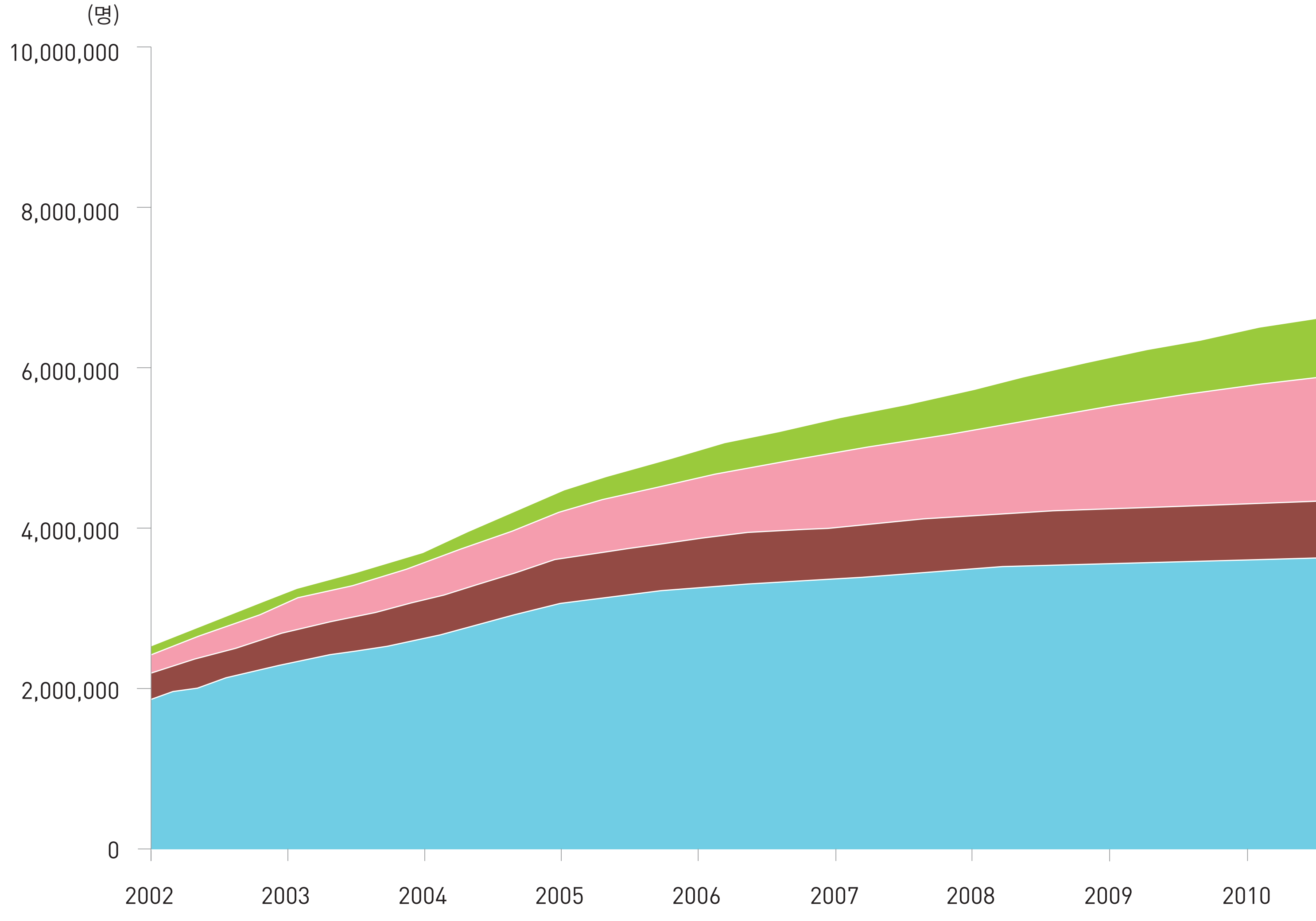

25%→57%

당뇨병 혹은 이상지질혈증
동반치료 증가

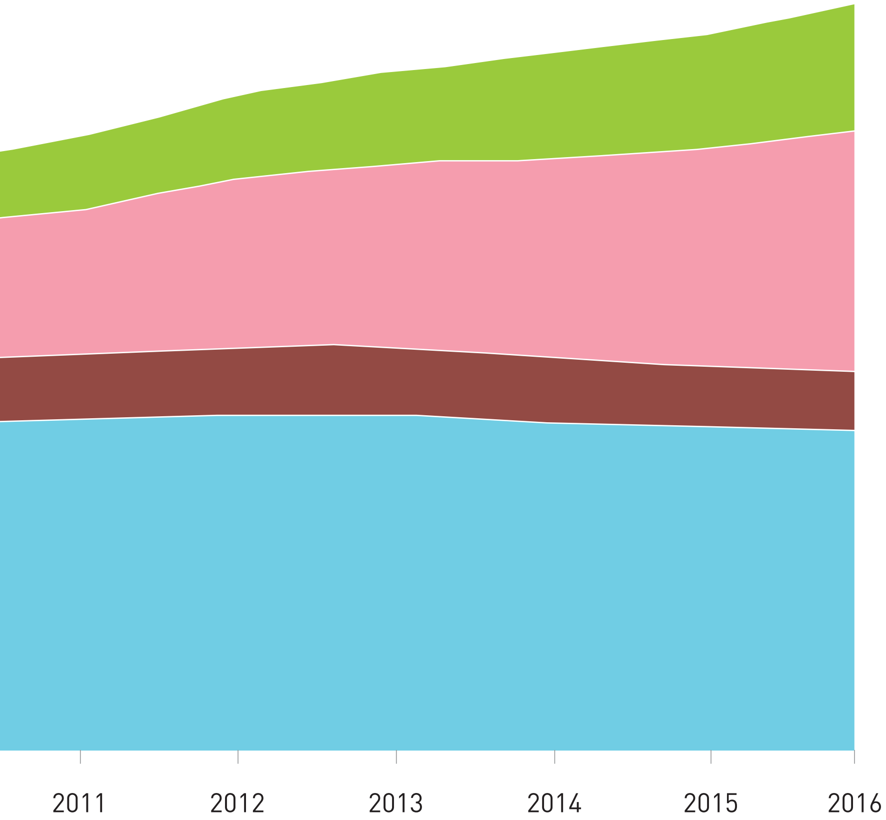

고혈압+이상지질혈증+당뇨병 치료
92,771(3.7%) → **1,407,011**(17.1%)

고혈압+이상지질혈증 치료
229,540(9.1%) → **2,621,509**(31.9%)

고혈압+당뇨병 치료
318,813(12.6%) → **638,706**(7.8%)

고혈압 치료
1,887,729(74.6%) → **3,551,878**(43.2%)

자료원: 국민건강보험공단 빅데이터 (2002-2016)

고혈압 약물 처방 변화 (전체치료자 중)

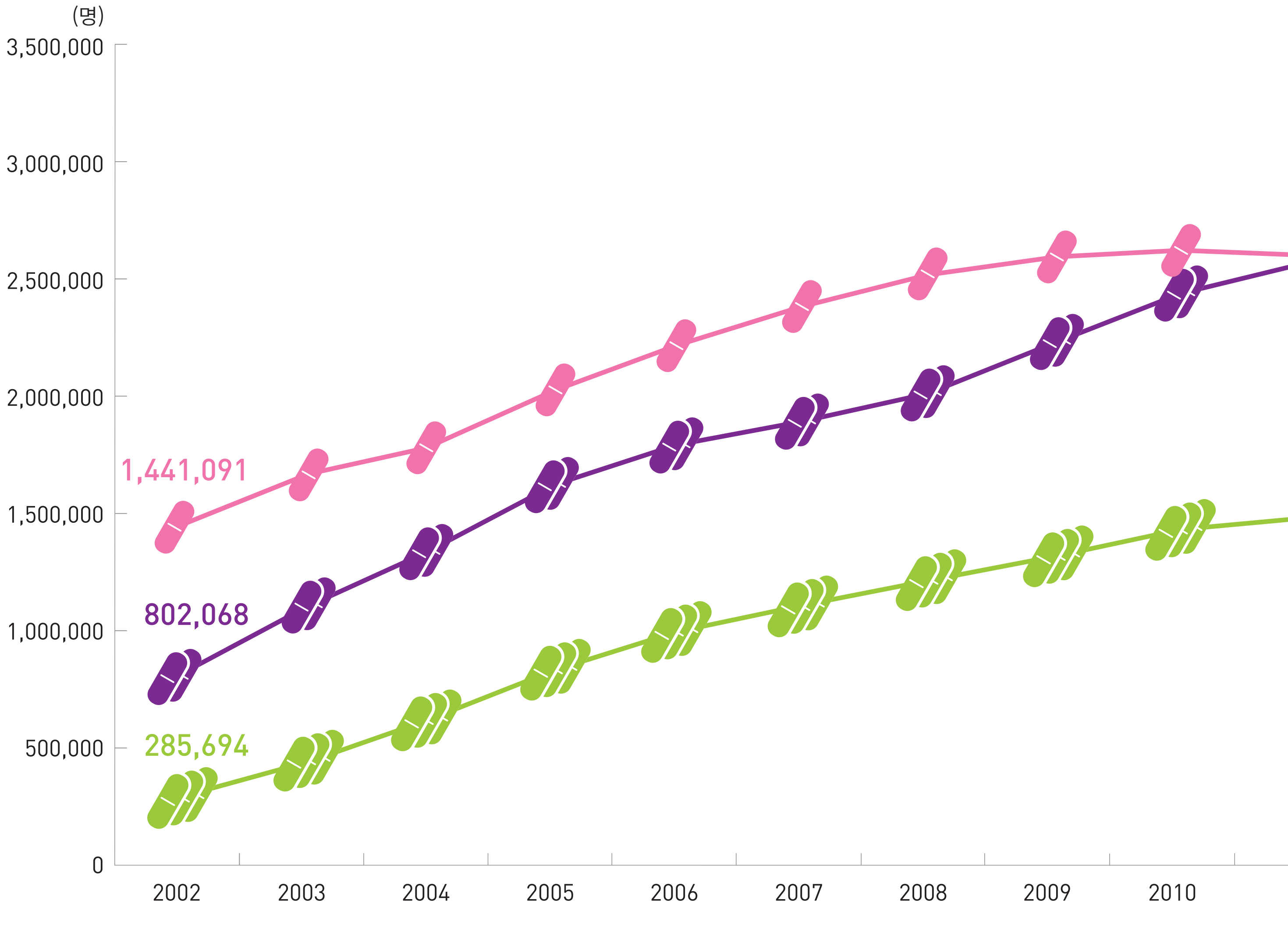

60%가 2제이상 복용

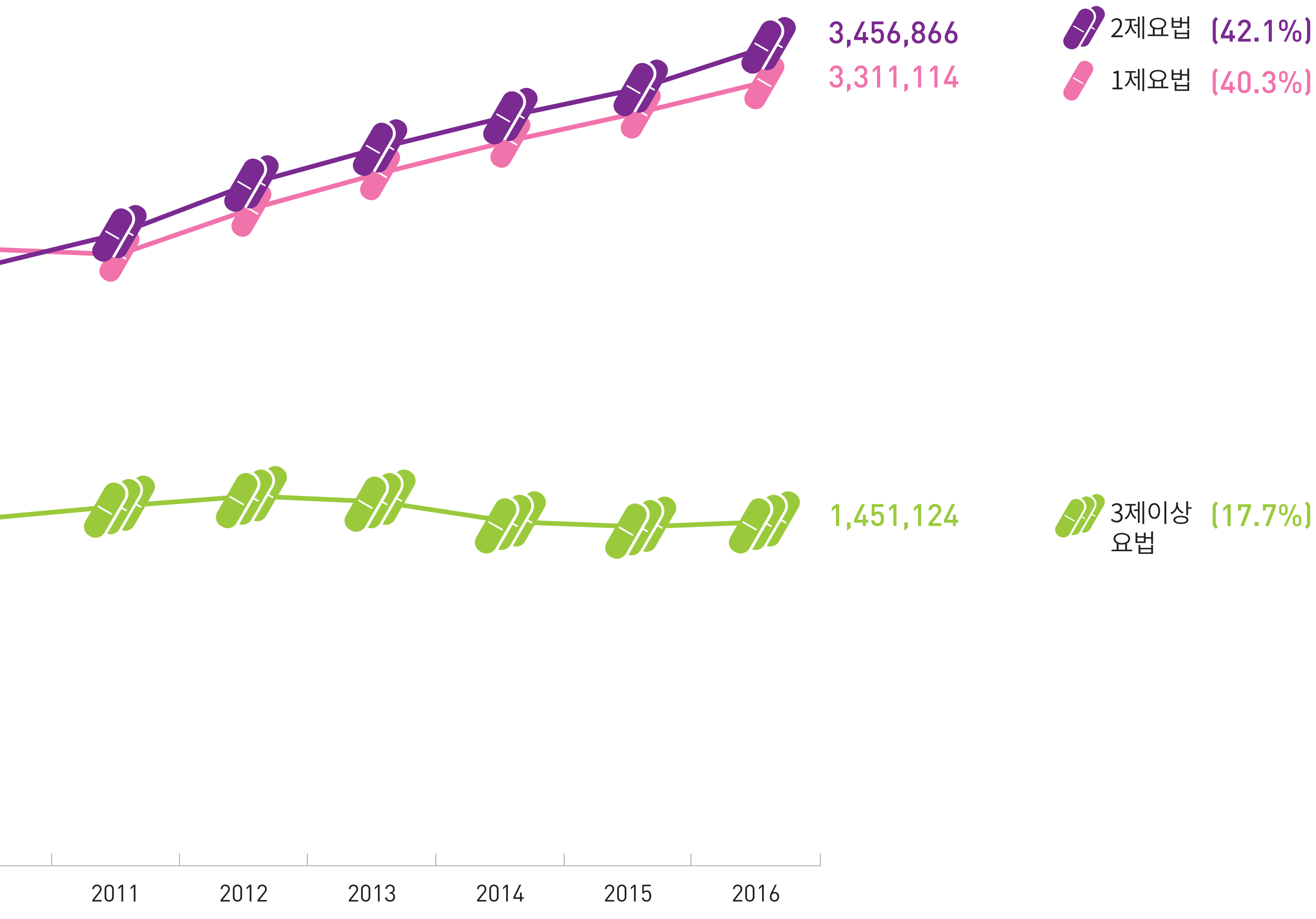

자료원: 국민건강보험공단 빅데이터 (2002-2016)

고혈압 약물 처방 변화 (지속치료자 중)

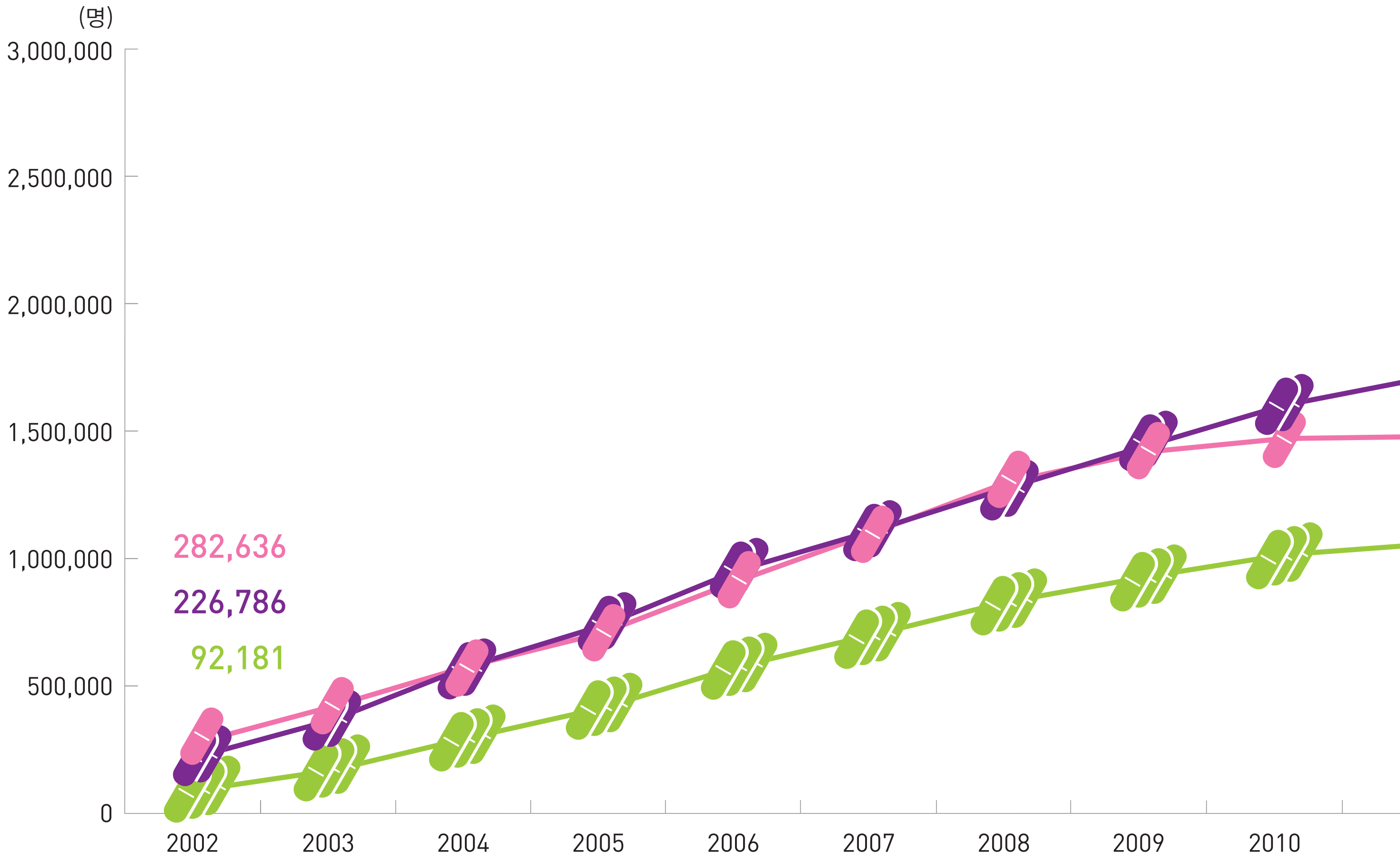

63%가 2제이상 복용

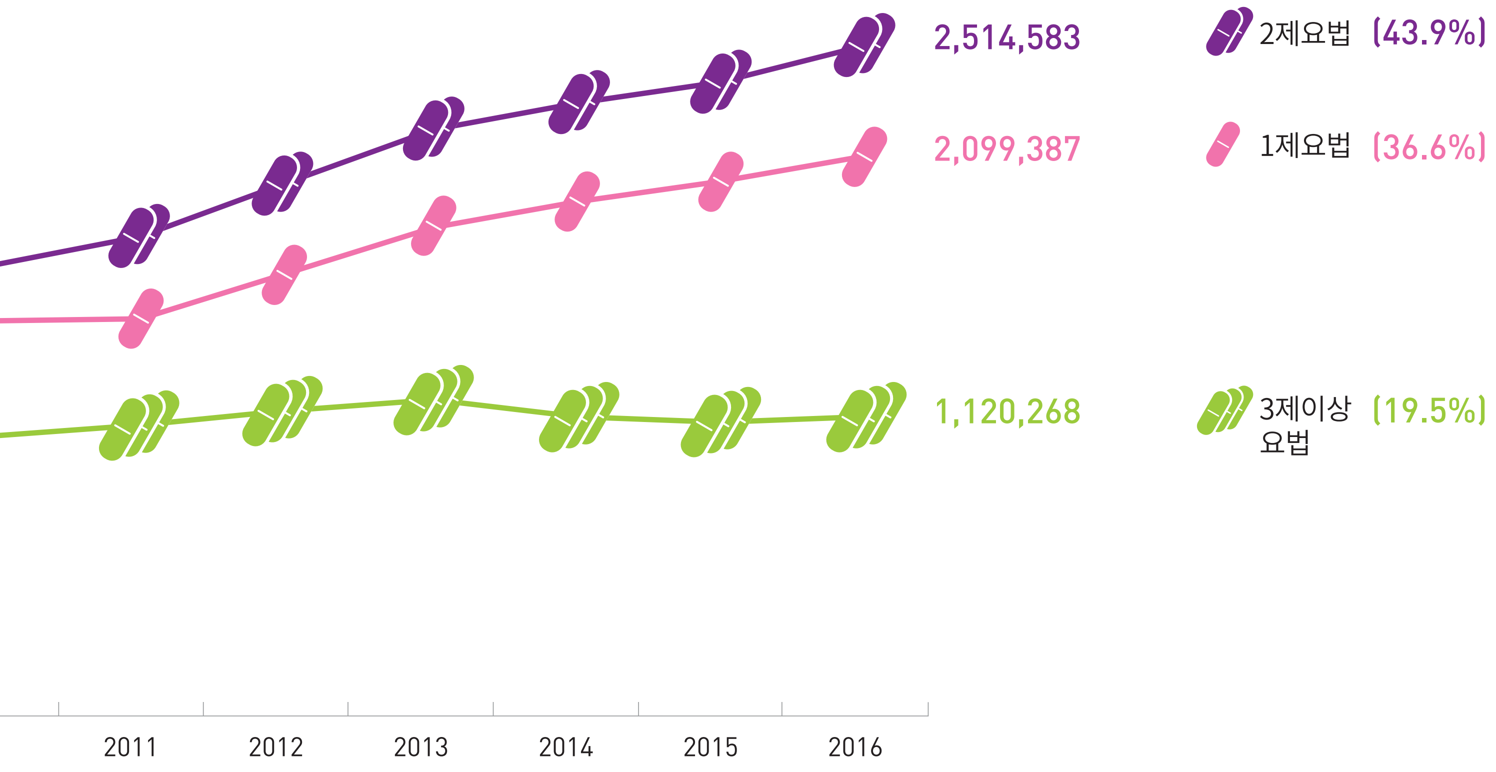

자료원: 국민건강보험공단 빅데이터 (2002-2016)

고혈압 약물 단독요법 구성 변화 (전체치료자 중)

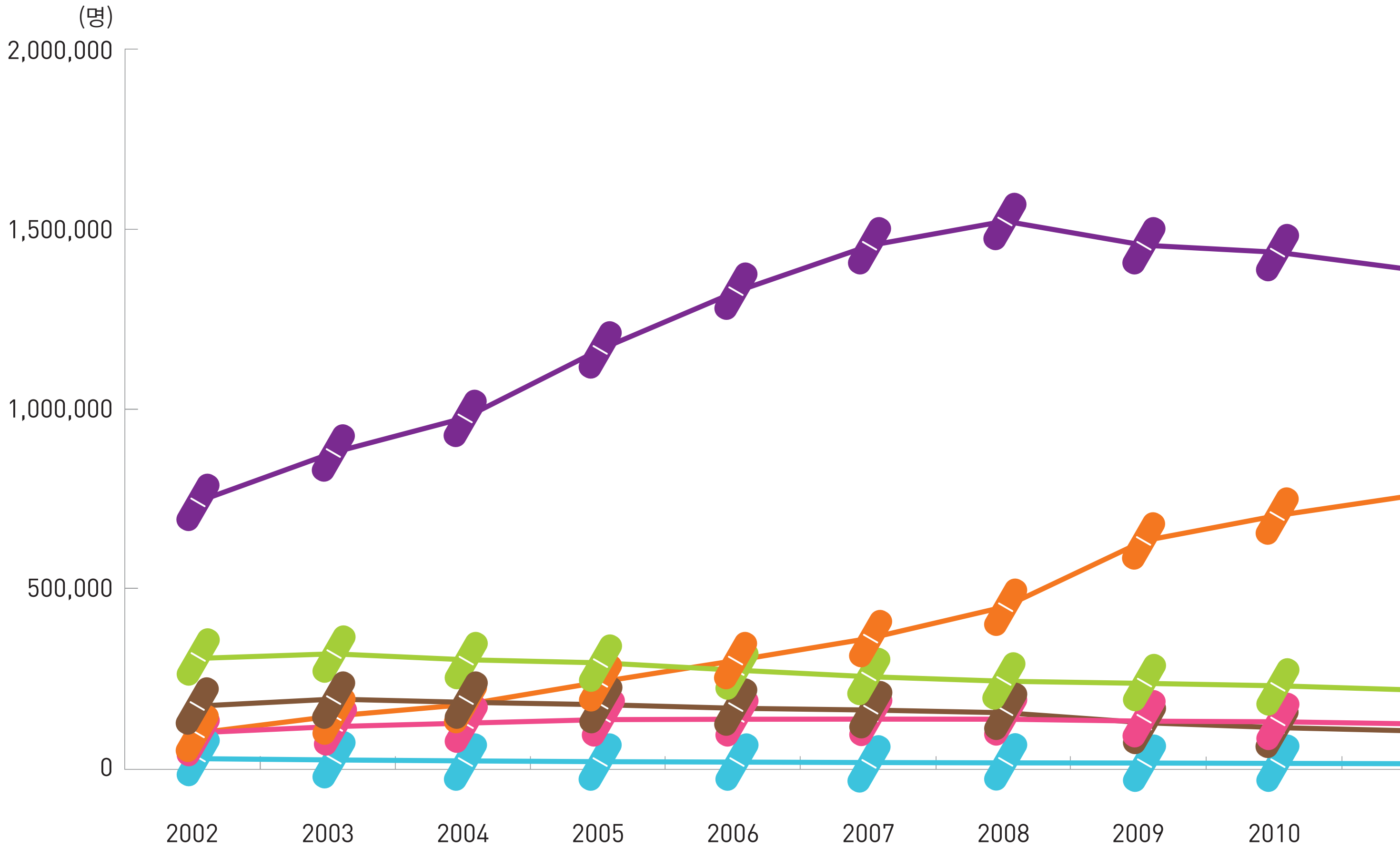

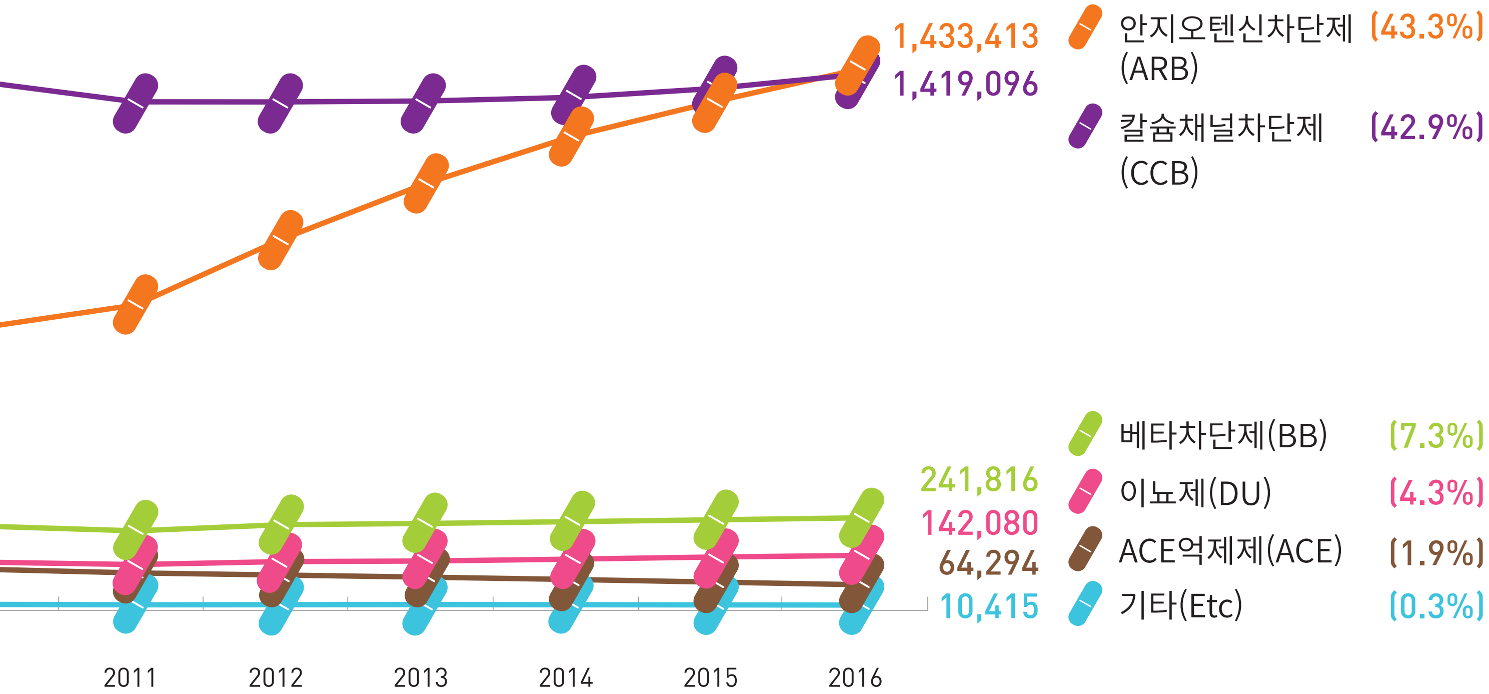

자료원: 국민건강보험공단 빅데이터 (2002-2016)

고혈압 약물 단독요법 구성 변화 (지속치료자 중)

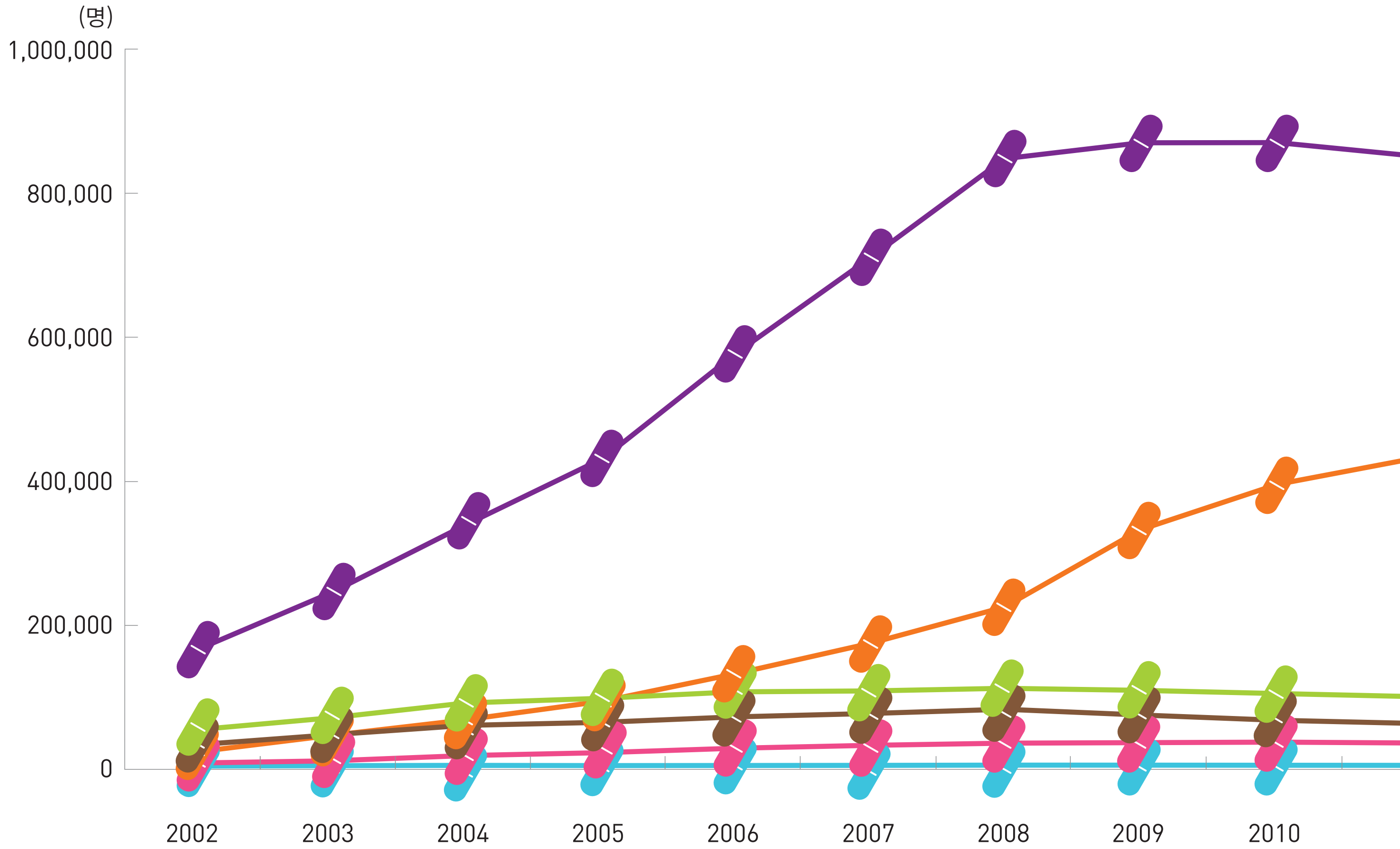

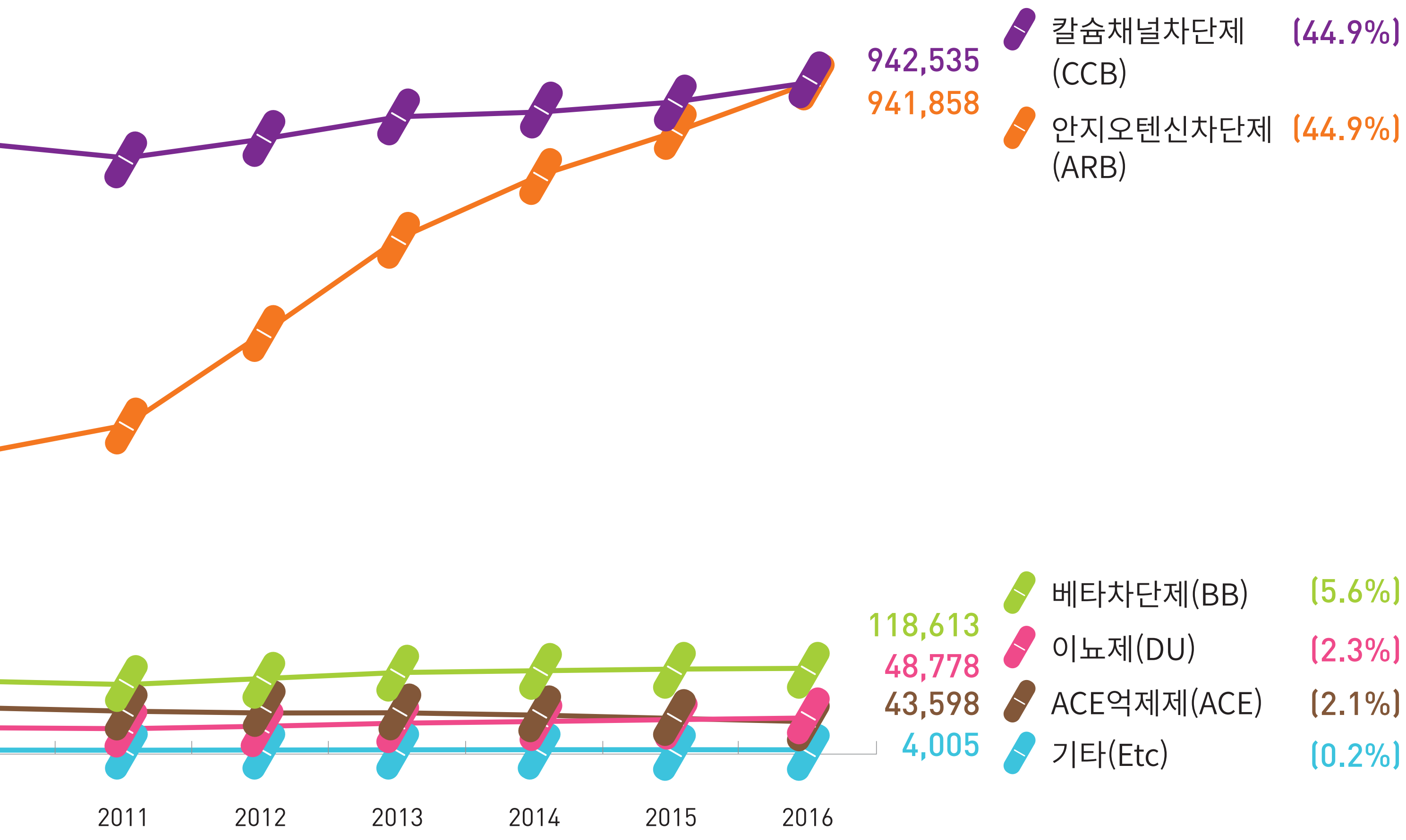

자료원: 국민건강보험공단 빅데이터 (2002-2016)

고혈압 2제요법 구성 변화 (전체치료자 중)

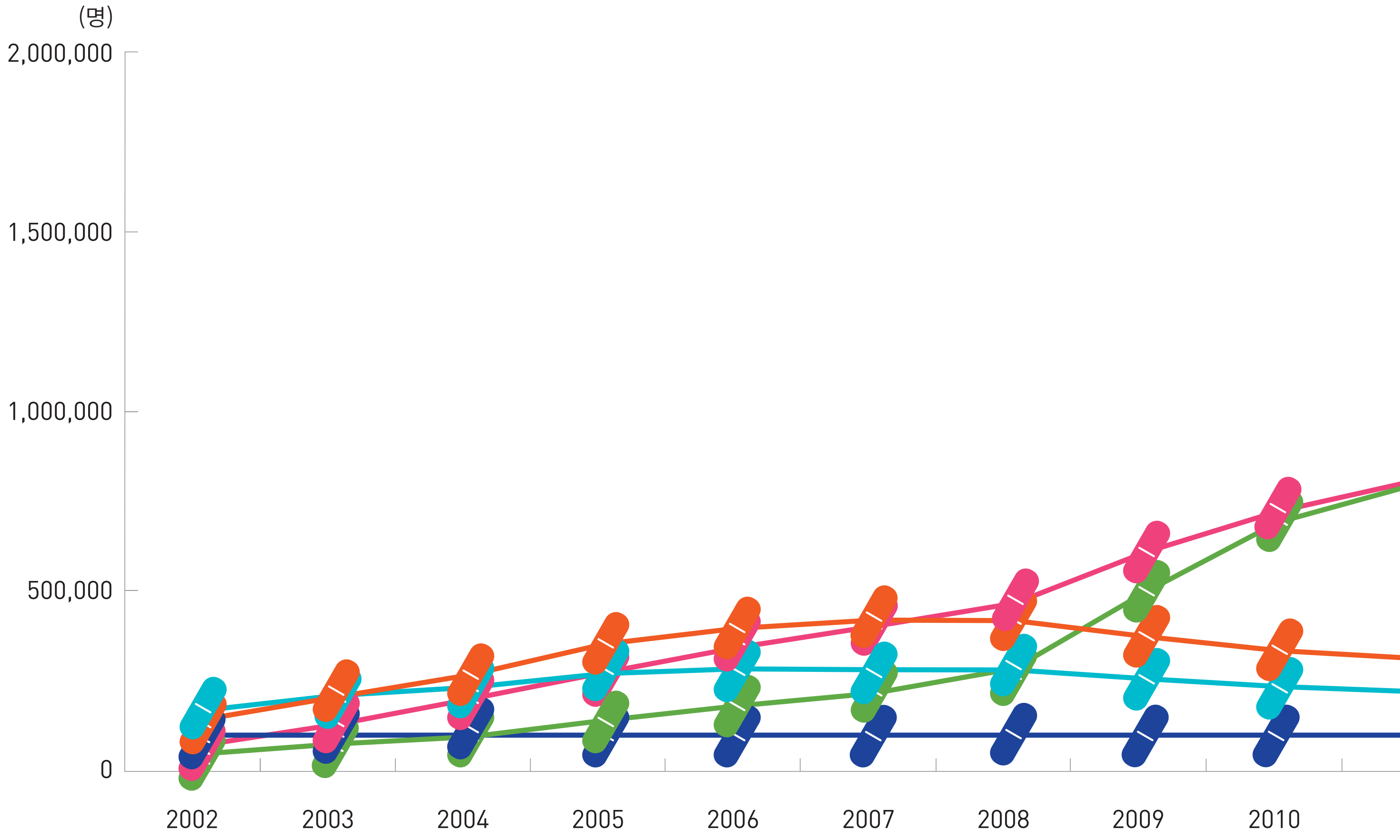

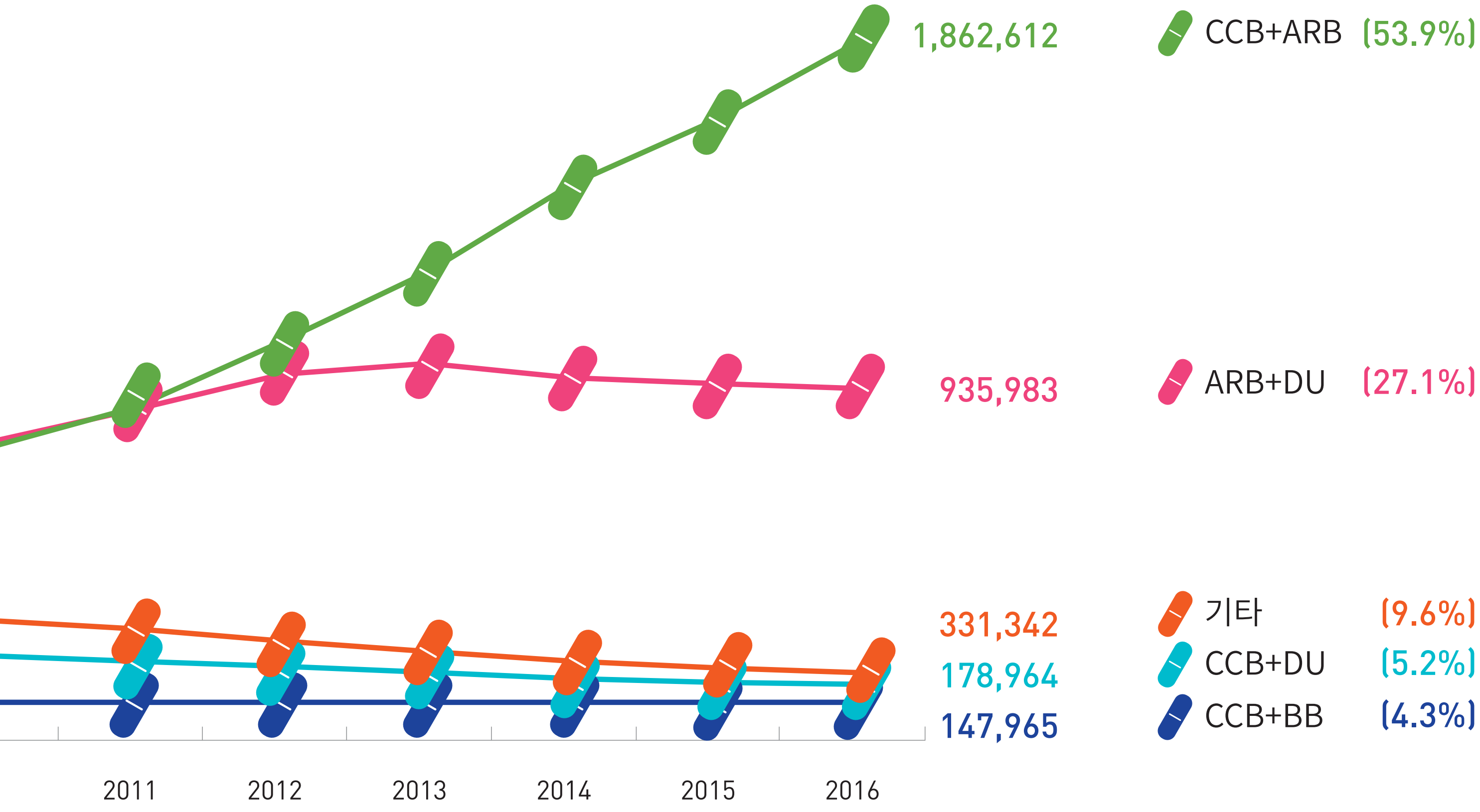

자료원: 국민건강보험공단 빅데이터 (2002-2016)

고혈압 2제요법 구성 변화 (지속치료자 중)

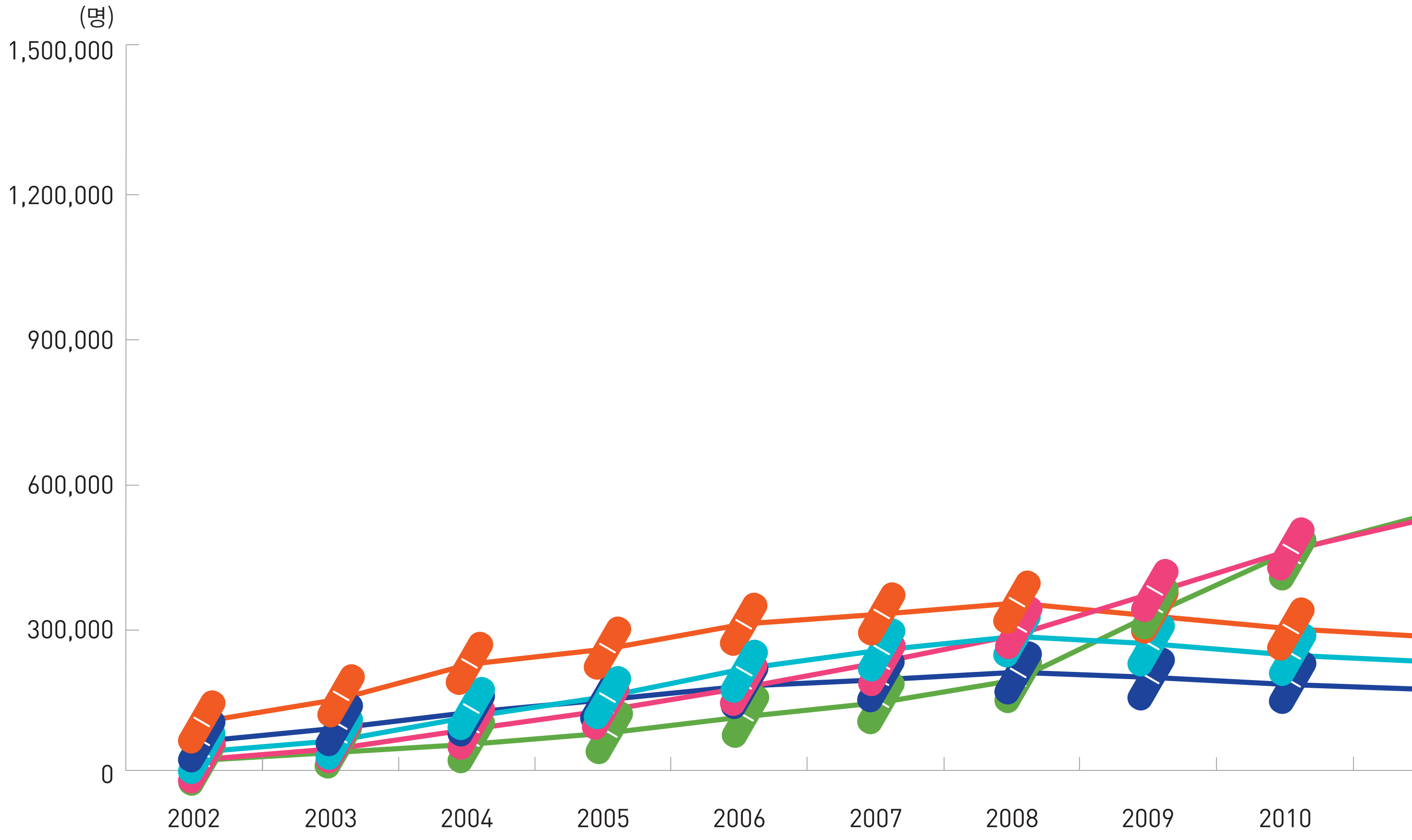

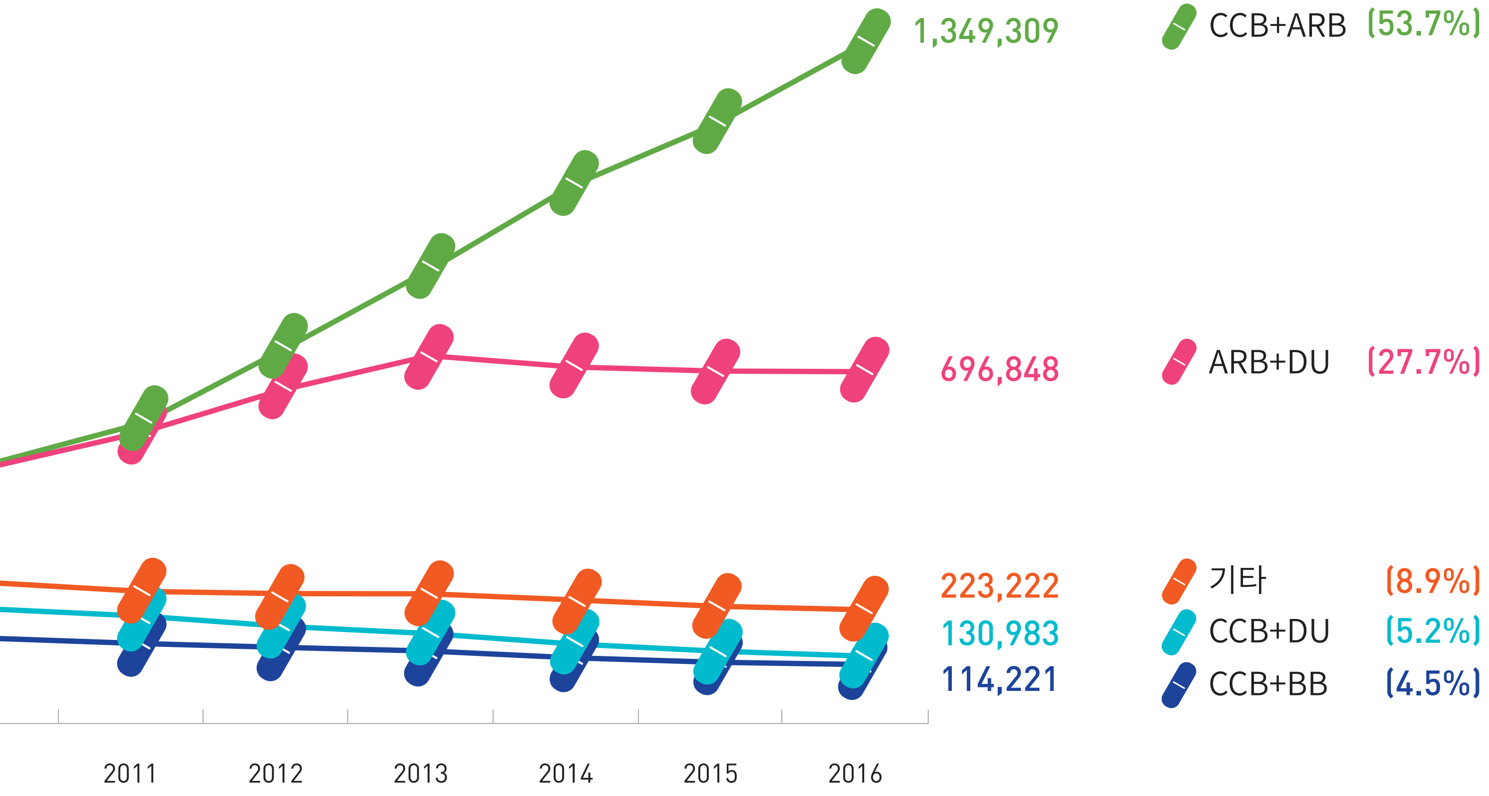

자료원: 국민건강보험공단 빅데이터 (2002-2016)

대한고혈압학회

THE KOREAN SOCIETY OF HYPERTENSION

www.koreanhypertension.org
